# Complete human day 14 post-implantation embryo models from naive ES cells

**DOI:** 10.1038/s41586-023-06604-5

**Published:** 2023-09-06

**Authors:** Bernardo Oldak, Emilie Wildschutz, Vladyslav Bondarenko, Mehmet-Yunus Comar, Cheng Zhao, Alejandro Aguilera-Castrejon, Shadi Tarazi, Sergey Viukov, Thi Xuan Ai Pham, Shahd Ashouokhi, Dmitry Lokshtanov, Francesco Roncato, Eitan Ariel, Max Rose, Nir Livnat, Tom Shani, Carine Joubran, Roni Cohen, Yoseph Addadi, Muriel Chemla, Merav Kedmi, Hadas Keren-Shaul, Vincent Pasque, Sophie Petropoulos, Fredrik Lanner, Noa Novershtern, Jacob H. Hanna

**Affiliations:** 1 Department of Molecular Genetics, Weizmann Institute of Science, Rehovot, Israel; 2 Department of Clinical Sciences, Intervention and Technology, Karolinska Institutet, Stockholm, Sweden; 3 Division of Obstetrics and Gynecology, Karolinska Universitetssjukhuset, Stockholm, Sweden; 4 Department of Development and Regeneration, Leuven Stem Cell Institute, Leuven Institute for Single-cell Omics (LISCO), KU Leuven-University of Leuven, Leuven, Belgium; 5 Department of Life Sciences Core Facilities, Weizmann Institute of Science, Rehovot, Israel; 6 Département de Médecine, Université de Montreal, Montreal, Quebec Canada; 7 Centre de Recherche du Centre, Hospitalier de l’Université de Montréal Axe Immunopathologie, Montreal, Quebec Canada; 8 Ming Wai Lau Center for Reparative Medicine, Stockholm Node, Karolinska Institutet, Stockholm, Sweden

**Keywords:** Induced pluripotent stem cells, Reproductive biology, Differentiation, Embryonic induction, Embryology

## Abstract

The ability to study human post-implantation development remains limited owing to ethical and technical challenges associated with intrauterine development after implantation^1^. Embryo-like models with spatially organized morphogenesis and structure of all defining embryonic and extra-embryonic tissues of the post-implantation human conceptus (that is, the embryonic disc, the bilaminar disc, the yolk sac, the chorionic sac and the surrounding trophoblast layer) remain lacking^1,2^. Mouse naive embryonic stem cells have recently been shown to give rise to embryonic and extra-embryonic stem cells capable of self-assembling into post-gastrulation structured stem-cell-based embryo models with spatially organized morphogenesis (called SEMs)^3^. Here we extend those findings to humans using only genetically unmodified human naive embryonic stem cells (cultured in human enhanced naive stem cell medium conditions)^4^. Such human fully integrated and complete SEMs recapitulate the organization of nearly all known lineages and compartments of post-implantation human embryos, including the epiblast, the hypoblast, the extra-embryonic mesoderm and the trophoblast layer surrounding the latter compartments. These human complete SEMs demonstrated developmental growth dynamics that resemble key hallmarks of post-implantation stage embryogenesis up to 13–14 days after fertilization (Carnegie stage 6a). These include embryonic disc and bilaminar disc formation, epiblast lumenogenesis, polarized amniogenesis, anterior–posterior symmetry breaking, primordial germ-cell specification, polarized yolk sac with visceral and parietal endoderm formation, extra-embryonic mesoderm expansion that defines a chorionic cavity and a connecting stalk, and a trophoblast-surrounding compartment demonstrating syncytium and lacunae formation. This SEM platform will probably enable the experimental investigation of previously inaccessible windows of human early post implantation up to peri-gastrulation development.

## Abstract (web summary)

The culture of genetically unmodified human naive embryonic stem cells in specific growth conditions gives rise to structures that recapitulate those of post-implantation human embryos up to 13–14 days after fertilization.

## Main

Implantation of the human embryo leads to a number of changes in organization that are essential for gastrulation and future development^1^. Much of this process relies on the morphogenesis of the extra-embryonic tissues and the effect this has on the organization of embryonic cells. Furthermore, this is a developmental stage with a high incidence of pregnancy loss and, for this reason, understanding the events associated with this period will benefit the understanding of fertility and developmental defects^5^. However, these studies have ethical and technical challenges. Although it is possible to culture structures derived from human blastocysts ex vivo, these cultures do not recapitulate the events and structural organization of the in vivo embryos^6^ (Supplementary Information).

Integrated stem-cell-derived embryo models of human post-implantation stages can provide a useful platform to understand these crucial stages of development^5^. Defining elementary hallmarks of human integrated post-implantation embryo models must include all of the following aspects: (1) the continued presence of equivalents of all key cell lineages of the developing early post-implantation embryo (for example, trophoblast-like, primitive endoderm (PrE)-like, extra-embryonic mesoderm (ExEM)-like and pluripotent epiblast-like cells); (2) clear self-organization of fundamental embryonic compartments with adequate morphological and structural organization and proper relative orientation between the latter structures (for example, embryonic disc-like, hypoblast-like, bilaminar-disc-like, amnion-like, polarized yolk sac (YS)-like, chorionic cavity (ChC)-like and trophoblast-like compartments); and (3) evidence of developmental dynamism relating to ability to progress, in a structurally organized manner, through morphologically characterized developmental milestones of the early post-implantation human embryo following initial aggregate formation^3^.

Recently, mouse naive embryonic stem (ES) cells were shown by our group to possess the ability to be coaxed ex utero into post-gastrulation-stage structured stem-cell-based embryo models with spatially organized morphology (previously called sEmbryos, synthetic embryos, stem-cell-derived synthetic whole embryo models or stembroids; called SEMs here)^3^. These structures result from the aggregation of non-transduced naive ES cells (which form the embryo proper) with naive ES cells transiently expressing the transcription factors CDX2 or GATA4 to promote their priming towards trophedctoderm (TE)-like lineages or PrE-like lineages, respectively. Mouse complete SEMs developed directly into egg-cylinder-shaped SEMs within complex extra-embryonic compartments and could dynamically advance beyond gastrulation and reach early organogenesis stages of development as late as embryonic day 8.5 (ref. ^3^). These findings established that mouse naive pluripotent cells can serve as the sole source of embryonic and extra-embryonic tissues in advanced complete ‘organ-filled’ embryo models^3^. Therefore, we reasoned that their counterpart may enable the generation of integrated SEMs from other mammalian species from which naive or naive-like pluripotent stem cells (PS cells, which refer to either ES cells or induced pluripotent stem cells (iPS cells)) have been stabilized, including humans^4,7^. Motivated by this achievement in mice^3^ and following developments in naive human PS cell culture conditions, we tested whether human naive (or naive-like) cells^4,7^ could be coaxed to form complex peri-implantation and post-implantation embryo-like structures that are able to dynamically advance to pre-grastulation and peri-gastrulation stages ex utero.

## ES cell priming towards extra-embryonic fates

**Fig. 1:**
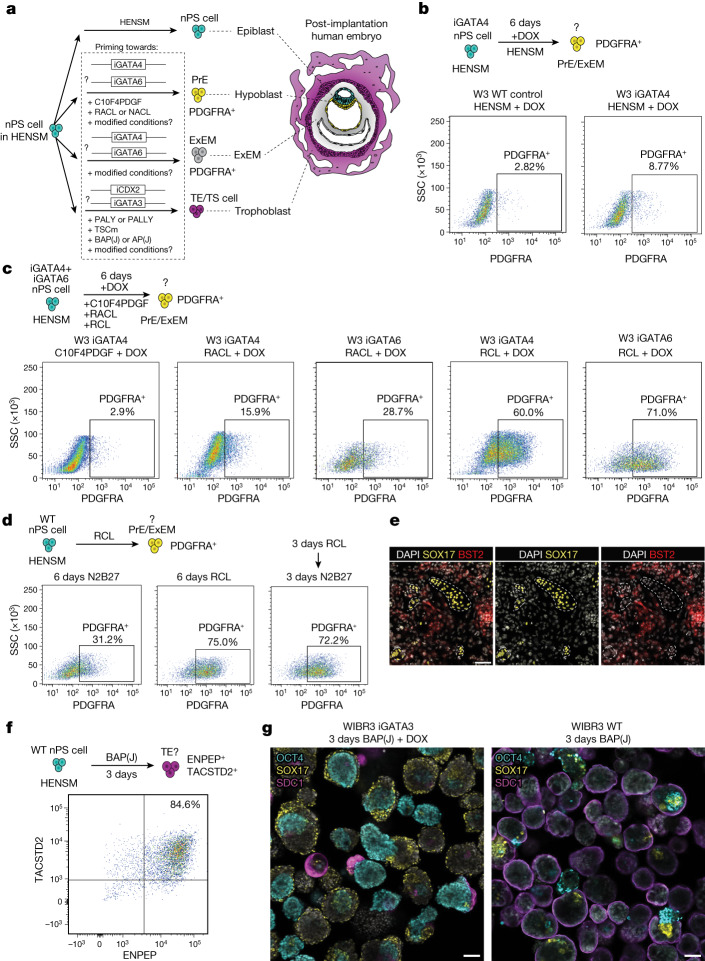
Optimizing human naive ES cell differentiation towards extra-embryonic lineages competent for early post-implantation SEM generation. **a**, Scheme of the tested induction (iGATA4, iGATA6, iCDX2 and iGATA3) and media conditions for generating the three different extra-embryonic lineages constituting the post-implantation human embryo (right) from HENSM naive PS cells (nPS cells). Epiblast (cyan), hypoblast (yellow), ExEM (grey) and trophoblast (magenta) compartments. **b**, Schematic (top) and FACS plots (bottom) of PDGFRA versus SSC for PrE-like and ExEM-like (PrE/ExEM) cell induction using iGATA4 with doxycycline (DOX) in HENSM after 6 days (right) and the control condition of naive cells (WT cells without iGATA4, left). **c**, Scheme (top) and FACS plots (bottom) of PDGFRA versus SSC for PrE/ExEM-like cell induction using iGATA4 and iGATA6 with DOX for 6 days in different media conditions as indicated (C10F4PDGF, RACL or RCL). **d**, Scheme (top) and FACS plots (bottom) of PDGFRA for PrE/ExEM-like cells using WT nES cells induced for 6 days in different media conditions (N2B27, RCL or RCL for 3 days followed by 3 days of N2B27). **e**, Immunofluorescence images of WT nES cells (WIBR3 line) induced for 6 days in RCL medium for SOX17, BST2 and nuclei (DAPI). Outline indicates mutually exclusive expression pattern of SOX17 and BST2. **f**, Scheme (top) and FACS plots (bottom) of ENPEP against TACSTD2 for TE-like lineage induction of HENSM nES cells (WIBR3 line) using the BAP(J) regimen for 3 days. **g**, Immunofluorescence images of day 6 SEM aggregates stained for OCT4, SOX17 and SDC1. The TE regimens used for SEM are indicated. Scale bars, 100 µm.

**Extended Data Fig. 1:**
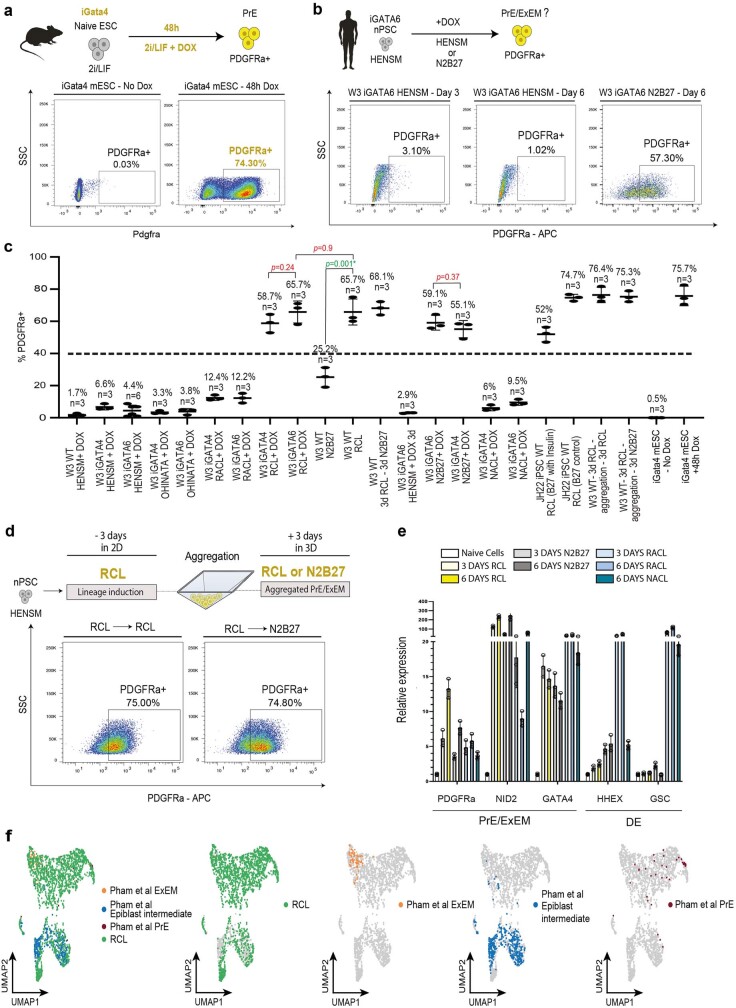
Optimization of extra embryonic lineage induction using transient overexpression of GATA4 and GATA6. **a**, representative Flow Cytometry (FACS) plots of Pdgfra-PE/Cy7 marking primitive endoderm (PrE)-like cells priming from mouse embryonic stem cells (ESC) using iGata4 with DOX for 48 h **(right)** versus the control condition without DOX **(left)**. **b**, representative FACS plots of PDGFRa-APC for putative PrE/ExEM-like priming from human naïve ESCs (nESCs) using iGATA6 with DOX in different media (N2B27 and HENSM) as indicated. **c**, quantification of the PDGFRa+ population by FACS analysis among the PrE/ExEM-like cell optimization conditions presented in this study (number of biological replicates is indicated for each condition). Average values and s.d. error bars are shown. Two-sided Student t-test *p* values are indicated where relevant. >40% PDGFRa+ set as a threshold for follow-up, followed by extended characterization and validation. **d**, representative FACS plots of PDGFRa-APC for putative PrE/ExEM-like priming/induction from human nESCs after three days in RCL medium (conventional 2D conditions) followed by three days of RCL **(left)** or basal N2B27 **(right)** in aggregation setting. **e**, representative RT-qPCR gene expression (normalized by GAPDH and ACTIN) of the endodermal marker genes, in RCL (yellow), RACL (blue), NACL (dark blue), and basal N2B27 (grey) media conditions versus naïve PCSs used as a control (white). PrE/ExEM and definitive endoderm (DE)-specific genes are separately underlined. Bar plot based panel showing the average value of each sample (which represents average value of 3 technical replicates), error bars indicate s.d. A single representative experiment out of N = 3 biological replicates performed is shown. **f**, integration of day 3 RCL starting cell population with Pham et al. 2022 ExEM conversion dataset^10^. UMAPs of RCL starting cell population integrated with the published reference dataset of day 6 RACL conversion protocol from naïve ESCs containing PrE cells, ExEM, and intermediate epiblast. Selected cell type annotations are shown. Source Data

In mice, deriving SEMs that contain all embryonic and extra-embryonic compartments requires optimal culture conditions and high-quality rapid priming towards PrE lineages and TE lineages from naive PS cells, which was achieved through the ectopic expression of Gata4 and Cdx2, respectively^3^. Hence, we first set out to establish a similar platform to rapidly and efficiently obtain extra-embryonic lineages through the transient expression of these transgenes in human PS cells (Fig. 1a). Note that the early post-implantation pre-gastrulation human, but not mouse, embryo contains an ExEM compartment^8^. We generated doxycycline-inducible human ES cells for GATA4 or GATA6, regulators of PrE and ExEM lineages in humans^9^ (Supplementary Fig. 1a,b). We used fluorescence-activated cell sorting (FACS) to analyze PDGFRA expression, which marks both PrE lineages and ExEM lineages^9^, to identify optimal conditions for rapidly and efficiently inducing naive ES cells grown in human enhanced naive stem cell medium (HENSM) and priming them towards PrE and/or ExEM-like lineages (Supplementary Fig. 1c–e). GATA4 induction (iGATA4) in mouse naive 2i/LIF conditions produced substantial upregulation of the PDGFRA^+^ cell fraction after 48 h of doxycycline treatment (Extended Data Fig. 1a). However, induction of GATA4 and GATA6 expression in human naive ES cells cultured in HENSM resulted in <10% PDGFRA^+^ cells after 6 days (Fig. 1b and Extended Data Fig. 1b). WNT stimulation by CHIR99021 can be a stimulant for mouse and human PrE induction^10^, and given that CHIR99021 is included in mouse but not in more recent and enhanced versions of human naive culture conditions^4^, we considered that HENSM conditions during the induction phase might not be suitable for human cells. Hence, we screened for other conditions to facilitate the induction of PDGFRA^+^ cells from naive human ES cells (Fig. 1a). The mouse PrE-derivation conditions (called C10F4PDGF)^11^ resulted in a very low level of PDGFRA induction (Fig. 1c and Extended Data Fig. 1c). RACL induction medium (RPMI-based medium supplemented with activin A, CHIR99021 and LIF), which has been used to prime human naive ES cells towards the PrE and ExEM states^10^, or NACL medium (based on DMEM/F12, neurobasal and N2B27 media) that stabilizes naive endoderm cells generated in RACL conditions^10^, also led to low levels of the PDGFRA^+^ fraction (Fig. 1c and Extended Data Fig. 1c).

Because activin A inhibits the in vitro differentiation of human naive ES cells into ExEM cells^9^, we omitted it from RACL medium (called RCL here). RCL medium resulted in PDGFRA induction in the majority (>50%) of cells in iGATA4 and iGATA6 cells (Fig. 1c). However, high efficiencies of PDGFRA^+^ cell formation was evident in RCL conditions from isogenic wild-type (WT) cells without exogenous expression of GATA4 or GATA6 (Fig. 1d), which indicated that transient transgene expression is not required for efficient PDGFRA^+^ induction in human naive HENSM ES cells. Further optimization showed that 3 days of induction in the RCL condition followed by 3 days of incubation in basal N2B27 conditions produced comparable results (Fig. 1d and Extended Data Fig. 1d). Notably, incubating naive ES cells in N2B27 medium also produced PDGFRA^+^ cells, but at significantly 2.5-fold lower levels than RCL medium (Fig. 1d and Extended Data Fig. 1c). As we preferred to use genetically unmodified cells, we focused on using RCL conditions on WT non-transgenic cells for further characterization.

**Extended Data Fig. 2:**
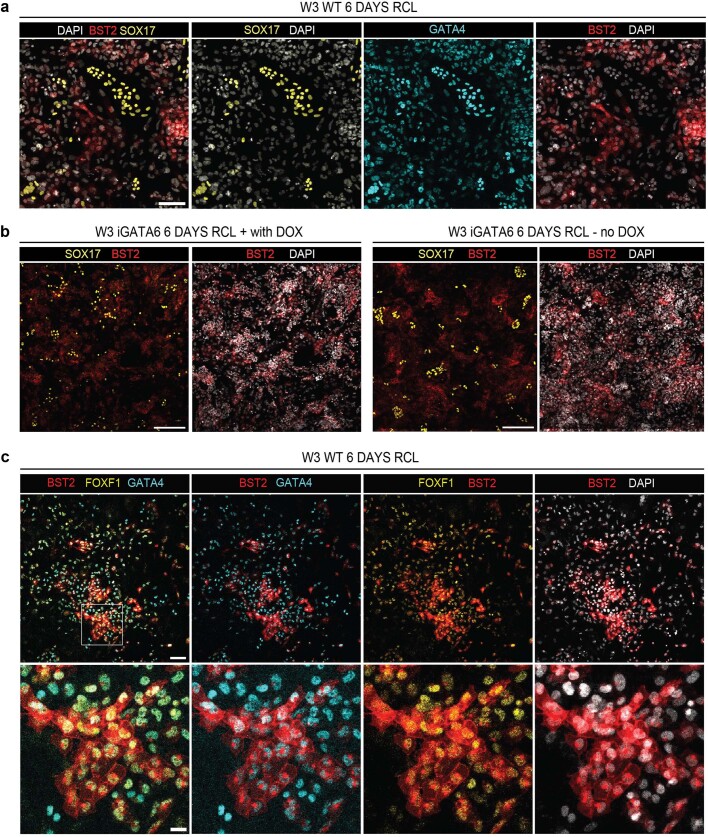
Optimization of PrE/ExEM-like cells priming protocol from human HENSM naïve ESCs. **a**, representative immunofluorescence images of WT WIBR3 (W3) nESCs induced in RCL media for 6 days, showing expression of GATA4 (cyan), SOX17 (yellow), and BST2 (red); nuclei (DAPI, white). GATA4 marks both SOX17+ PrE-like and BST2+ ExEM-like populations (see also Fig. 1e). This panel is an extended version of Fig. 1e but showing staining patterns of more markers. Scale bar, 100 µm. **b**, representative immunofluorescence images of iGATA6 nESCs induced in RCL media for 6 days (with or without DOX), showing expression of SOX17 (yellow) and BST2 (red). In both set-ups, BST2^+^ (ExEM-like) and SOX17^+^ (PrE-like) cell populations have a mutually exclusive expression pattern. Scale bar, 200 µm. **c**, representative immunofluorescence images of WT nESCs in RCL media for 6 days. FOXF1 (yellow), BST2 (red), GATA4 (cyan), nuclei (DAPI, white). BST2^+^ cells are also GATA4^+^/FOXF1^+^, excluding the possibility that they represent residual pluripotent cells in the RCL induced cultures from HENSM ESCs. Scale bar, 100 µm. Bottom, 4x zoom, scale bar, 25 µm.

We tested for the existence of PDGFRA^+^ PrE-like and/or ExEM-like cells in RCL induction conditions and aimed to distinguish between them. Both immunostaining and PCR results validated the endogenous expression of PrE markers in RCL conditions, including SOX17, which marks only the PrE fraction, alongside GATA4, GATA6 and NID2, markers that are common between PrE and ExEM cells^9^ (Extended Data Fig. 1e and Supplementary Fig. 2). Markers of definitive endoderm (DE)-like GSC or HHEX^10^ were not meaningfully induced from naive ES cells in RCL conditions (Extended Data Fig. 1e), which therefore excluded DE identity. Applying RCL medium on human isogenic primed ES cells produced higher DE marker expression (GSC or HHEX), as expected^10^ (Supplementary Fig. 1f). Immunostaining results for SOX17 and BST2 markers that distinguish between PrE and ExEM lineages^9^, respectively, confirmed the emergence of both PrE-like and ExEM-like cells from naive ES cells under the same RCL induction protocol using MEFs (with or without GATA6 overexpression) (Fig. 1e and Extended Data Fig. 2a,b). GATA4 positively marked both SOX17^+^ PrE-like and BST2^+^ ExEM-like populations as expected^9^ (Extended Data Fig. 2a). BST2^+^ ExEM-like cell identity was validated by the upregulation of FOXF1 (Extended Data Fig. 2c), which marks ExEM cells, but not PrE cells or residual ES cells. To examine the identity of the starting day 3 RCL cells, we applied single-cell RNA sequencing (scRNA-seq) and integrated our data with a reference dataset of naive-to-PrE and ExEM cell differentiation^9^. Day 3 RCL cells derived from HENSM naive ES cells aligned to previously described PrE cells, ExEM cells and some residual PS cells (intermediate epiblast cells) (Extended Data Fig. 1f and Supplementary Figs. 1g and 3). Thus, we adopted RCL pretreatment of WT HENSM naive PS cells for aggregation experiments with other lineages.

**Extended Data Fig. 3:**
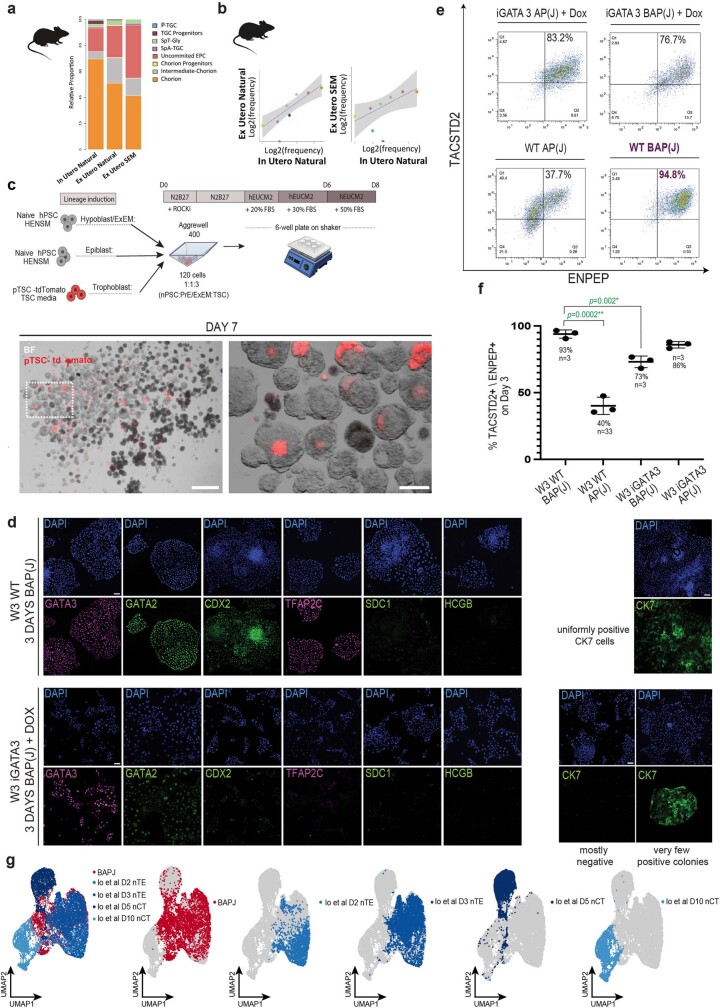
Testing SEM aggregation conditions with human conventional trophoblast stem cell lines and evaluating trophoblast induction with transient overexpression of GATA3 in human naïve ESCs. **a**, proportion of the indicated cell types among extra-embryonic cells of mouse natural embryos grown *in utero* or *ex utero* and in day 8 mouse SEMs generated from iCdx2 mouse naïve ESCs (and not embryo derived mouse TSC lines)^3^. Three pooled samples are presented: *in utero* natural embryos (n = 2401 cells), *ex utero* natural embryos (n = 1382), and mouse iCdx2 day 8 SEMs (n = 6249). The cell types: Chorion, Intermediate-Chorion, Chorion Progenitors, Uncommitted Ectoplacental-Cone Cells (EPC), Trophoblast Giant Cells (TGC) progenitors, parietal trophoblast giant cells (pTGC), spiral artery associated trophoblast giant cells (SpA-TGC), and junctional zone spongiotrophoblast cells (SpT-Gly) based on previously published similar analysis and annotations^25^. **b**, frequencies of the cell types presented in **(a)**, showing a significant reduction of TGC-progenitors and pTGCs in *ex utero* embryos (natural and SEM), compared to *in utero* embryos. Blue line represents the linear function f(x)=x; the Shaded area represents 95% confidence interval. Contrary to previous conclusions^25^, this analysis confirmed that mouse naïve ESCs derived TSC lineage following Cdx2 overexpression under optimized conditions developed in^3^ can contribute to both the chorionic and ectoplacental cone-like (EPC) lineages in mouse SEMs generated exclusively from mouse naïve ESCs (and not only to the chorionic compartment as claimed by Lau and colleagues^25^). It is likely that failure to detect PGCs and EPC in Lau et al.^25^ mouse SEM results from their different and suboptimal TSC (and extra embryonic ectoderm - EXE) induction condition used, in comparison to the one devised in Tarazi et al.^3^. **c (top)**, scheme of the aggregation protocol for epiblast (naïve hESCs in HENSM media) and PrE/ExEM-like cells with the validated human TSC line derived from human primed ESCs (termed pTSC) and expressing tdTomato. **c (bottom)**, representative brightfield images and live fluorescence of tdTomato (red) in aggregates with labeled TSCs; scale bar, 200 µm. Right, zoom into the several SEMs with tdTomato signal; scale bar, 50 µm. **d**, representative immunofluorescence images showing different patterns of expression of trophoblast marker genes in the wild type (WT) nESCs incubated in BAP(J) media for three days **(top)** versus iGATA3 cells, induced by DOX in BAP(J) media **(bottom)**. GATA3 (magenta), TFAP2C (magenta), GATA2, CDX2, SDC1, CK7, and HCGB (all in green), nuclei (DAPI, blue). Scale bars, 100 µm. **e**, representative FACS plots of ENPEP versus TACSTD2 for trophoblast (Tb)-like cell priming using iGATA3 induction in different media (AP(J) with DOX, BAP(J) with DOX), and using WT nESC priming to trophectoderm in AP(J) and BAP(J) regimens. Percentage of double positive population is indicated. **f**, quantification of the ENPEP-positive/TACSTD2-positive population (%) across conditions in **(e)**, n = 3 biological replicates; average value and s.d. error bars are indicated per condition. Two-sided Student’s t-test *p* values are indicated where relevant. **g**, integration of BAP(J) starting population with previously published in vitro differentiation derived (Io et al. 2021)^14^ naive trophectoderm conversion data. UMAP of Day 3 BAP(J) starting cell population derived from HENSM were integrated with the previously published reference dataset^14^ of naive trophectoderm (nTE) and naive cytotrophoblast (nCT) following BAP(J) regimen. Selected cell type annotations are shown. Source Data

We set out to find optimal conditions for priming naive ES cells towards TE lineage cells that can adequately aggregate with the other lineages and develop into post-implantation-stage human SEMs. CDX2 overexpression in mouse naive ES cells can efficiently and rapidly generate trophoblast stem (TS) cells that remain viable and correctly integrated after aggregation with mouse naive ES cells and iGATA4 cells, and can generate both chorionic and ectoplacental cone placental lineages in mouse SEMs^3 ^(Extended Data Fig. 3a,b). We generated doxycycline-inducible CDX2 (iCDX2) human ES cell lines with a constitutively expressed tdTomato marker to track cell viability and integration (Supplementary Fig. 4a–c). Human iCDX2 cells pretreated with doxycycline in either HENSM or different validated human TE and TS cell induction conditions did not meaningfully expand within the aggregates generated with induced PrE-like and ExEM-like cells and ES cells. This result is probably due to the substantially reduced viability of iCDX2 cells following doxycycline treatment (Supplementary Fig. 4d–f). We also tested tdTomato-labelled TS cell lines derived from both naive and primed ES cells^12^ (Supplementary Fig. 5a). In all tested aggregation conditions with either primed (Extended Data Fig. 3c and Supplementary Fig. 5) or naive ES-cell-derived TS cell lines (Supplementary Fig. 6a), the TS cells did not generate an outer layer surrounding the aggregate but instead formed focal clumps (Extended Data Fig. 3c and Supplementary Figs. 5 and 6b–d). The inability of human TS cells to integrate adequately within putative SEMs, as opposed to mouse TS cells, might stem from the fact that mouse TS cell lines are CDX2^+^ and correspond to equivalent earlier stages of trophoblast development than those TS cells isolated from human ES cells, which are CDX2^−^ (refs. ^13,14^).

We also tested induced GATA3 (iGATA3) ES cells. PCR analysis showed higher expression levels of CDX2 and TACSTD2 in the non-transduced group under BAP(J) culture conditions (DMEM/F12-based medium with the ALK4, ALK5 and ALK7 inhibitor A83-01, the ERK and MEK inhibitor PD0325901, and BMP4 for 24 h and substituted with a JAK inhibitor for the next 48 h)^14^ (Supplementary Fig. 7). Although GATA3 overexpression induced the endogenous expression of the TE marker GATA2, the cells did not uniformly express TFAP2C, CDX2 or cytokeratin 7 (CK7). By contrast, in the absence of transgene overexpression, we observed high and uniform expression of TFAP2C and CDX2 and a high occurrence of CK7^+^ cells under BAP(J) conditions (Extended Data Fig. 3d). Flow cytometry analysis for TACSTD2 (a marker of early and late TE cells) and ENPEP (expressed only in the late TE cells)^14^ showed the highest percentage of a double-positive population in WT cells under BAP(J) conditions, and their TE-like identity was validated by scRNA-seq^14^ (Fig. 1f and Extended Data Fig. 3e–g).

**Extended Data Fig. 4:**
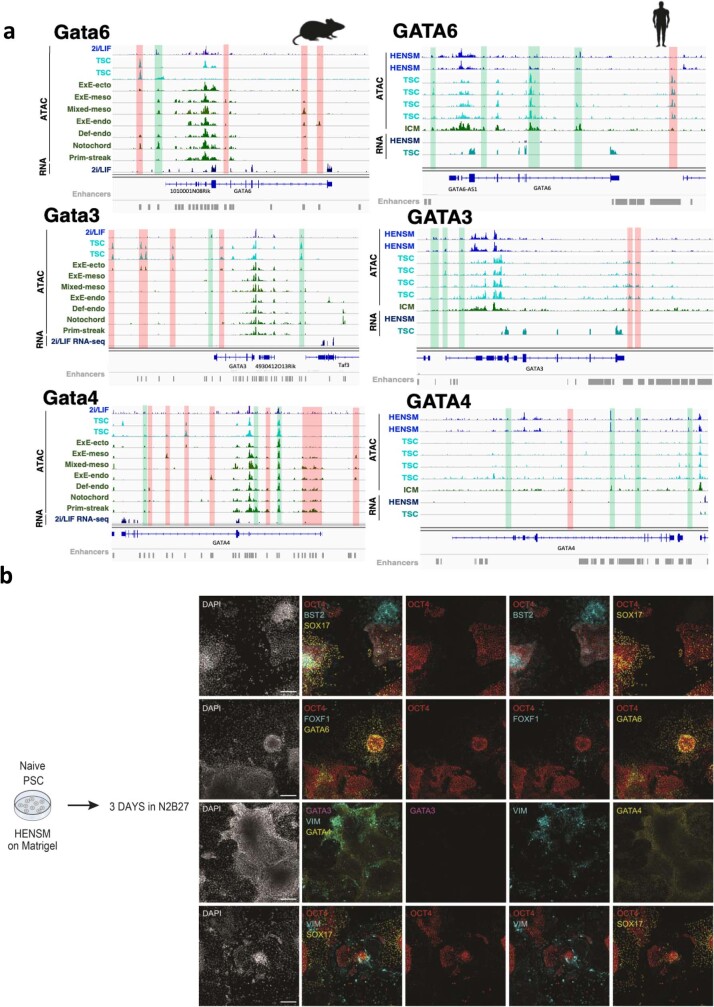
Enhancer accessibility of extra-embryonic lineage master regulators GATA3, GATA4 and GATA6 in human but not mouse naïve ESCs. **a**, ATAC-seq and RNA-seq of GATA3, GATA4 and GATA6 genes in human vs. mouse, as measured in naïve ESCs and in multiple differentiated cell types (as indicated). HENSM conditions were used for human naïve ESCs^4^. Known enhancers are marked in grey bars at the bottom. Putative enhancers are marked in red or green: (1) green indicates potential enhancers that are already open in naïve stem cells, (2) red indicates enhancers that are closed in naïve pluripotent stem cells, but open in at least one differentiated cell state. Putative enhancers in the approximate regions of the genes were manually selected. Open regions that overlap with promoter or exon were excluded from the analysis. RNA-seq of the indicated genes in naïve ESCs are shown. **b**, immunofluorescence images of HENSM ESCs cultured for 3 days in N2B27 basal medium on Matrigel-coated palates. OCT4 marks pluripotent cells (red); VIM, FOXF1, and BST2 mark the ExEM-like cells (all in cyan); SOX17 and GATA6 mark PrE- and PrE/ExEm-like cells, respectively (yellow). GATA3 marks trophoblast-like cells (magenta), nuclei (DAPI, white). Scale bar, 200 µm.

Notably, following induction and aggregation with naive ES cells and PrE-like and ExEM-like cells, iGATA3 cells remained viable but did not surround the aggregates (Fig. 1g). By contrast, TE-like cells derived from genetically unmodified naive ES cells under the same BAP(J) protocol uniformly surrounded the aggregates (Fig. 1g). This finding is a decisive criterion that is expected to be fulfilled in integrated SEMs, as the crosstalk of the TE lineage with the rest of the embryo and its role in proper morphogenesis continues to be an open question in human development. The ability to derive relevant extra-embryonic lineages from genetically unmodified WT human naive ES cells without the need for transgene overexpression is in line with recent studies demonstrating that human naive pluripotent cells can be more easily coaxed to give rise to early progenitors of PrE-like, amnion-like, ExEM-like and TE-like cells when compared to mouse naive ES cells, which require the overexpression of ectopic transcription factors^4,9,14,15^. This is consistent with our observation that enhancers of key TE and PrE regulators (GATA3, GATA6 and GATA4) are accessible in human but not in mouse naive ES cells while being transcriptionally inactive in both (Extended Data Fig. 4a). The latter might render human naive ES cells to be relatively more responsive to the addition or omission of signalling cues to activate endogenous *GATA3*, *GATA6* and *GATA4* without the additional obligatory need for their ectopic expression to induce naive ES cell fates towards extra-embryonic lineages.

**Extended Data Fig. 5:**
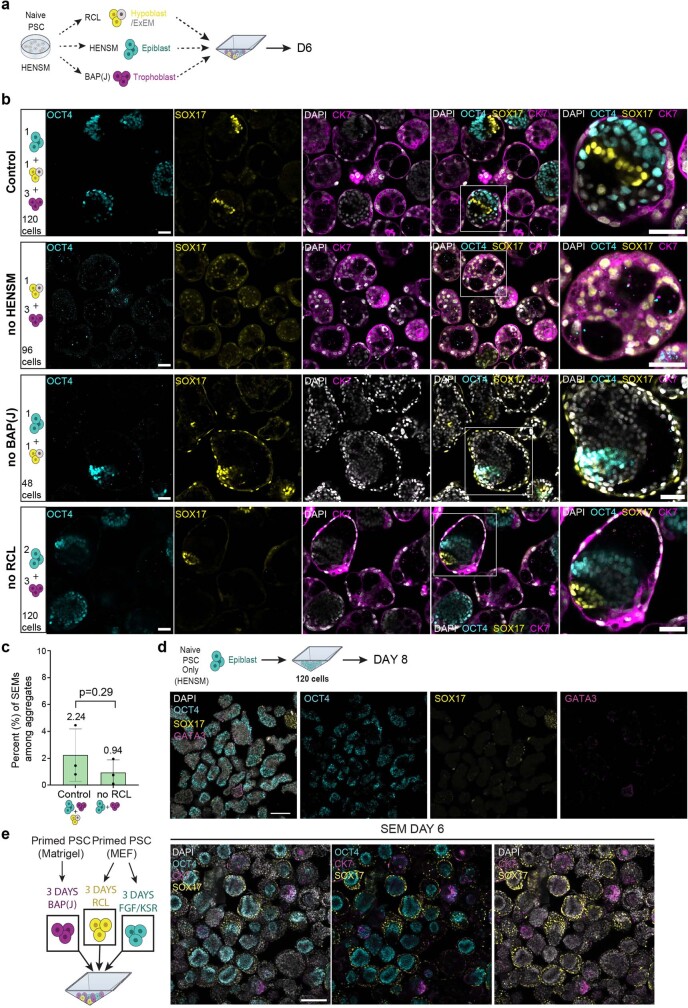
Evaluating SEM aggregation by omitting one of the lineages. **a**, scheme of the aggregation experiment (see Methods) where one of the lineages was omitted to evaluate its contribution to the resulting SEM morphology. **b (from top to bottom)**, representative immunofluorescence images of day 6 SEM aggregates from the three lineages (control, 120 cells), no HENSM (1:3 RCL: BAP(J), 96 cells), no BAP(J) (1:1 HENSM: RCL, 48 cells), and no RCL cells (2:3 HENSM: BAP(J), 120 cells). **b (from left to right)**, representative immunofluorescence images of day 6 SEM aggregates showing OCT4 (cyan), SOX17 (yellow), and CK7 (magenta); nuclei (DAPI, white). Right, zooms into the SEMs from different aggregation conditions. Scale bars, 50 µm. **c**, quantification of the SEM efficiency from the control aggregation and the aggregation without RCL cells (Pre/ExEM-like cells), as judged by the formation of bilaminar Epi/Hb-like structure surrounded by the trophoblast-like layer. N = 3 (across 344, 157, and 1244 aggregates) and N = 3 (across 1063, 205, 929 aggregates) for control and no RCL conditions, respectively. Bars show mean values, whiskers mark s.d. p-value = 0.29; two-sided unpaired student t-test. **d**, representative immunofluorescence images of day 6 aggregates made solely from naïve ESC from HENSM media showing OCT4 (cyan), low SOX17 (yellow), and GATA3 (magenta); nuclei (DAPI, white). Scale bar, 200 µm. **e (left)**, scheme of the experiment testing the capacity of cells, differentiated from human WIBR3 primed ESCs, to form equivalent SEMs to those obtained from isogenic naïve ESCs expanded in HENSM conditions. **e (right)**, representative immunofluorescence images of day 6 SEMs showing OCT4 (cyan), SOX17 (yellow), CK7 (magenta), and nuclei (DAPI, white). When starting with isogenic WIBR3 primed ESCs, the resulting aggregates did not present organization and maturation of the key embryonic and extra-embryonic compartments as seen when starting with HENSM naïve ESCs (e.g., Fig. 2). Scale bar, 200 µm. Source Data

To evaluate the projected contribution of the three induced populations aggregated together at day 0, we interchangeably omitted each of the input cell fractions (HENSM cells, BAP(J)-induced and RCL-induced cells) (Extended Data Fig. 5a). Omitting HENSM ES cells abolished OCT4^+^ epiblast-like formation, whereas CK7^+^ trophoblast-like cells formed and surrounded the aggregates that still contained disorganized SOX17^+^ cells (Extended Data Fig. 5b), which suggests that only HENSM-induced cells can give rise to the epiblast-like compartment. Omitting day 3 BAP(J)-induced TE-like cells abolished the formation of the trophoblast-like compartment (Extended Data Fig. 5b), which demonstrated that the trophoblast-like compartment is derived only from the induced BAP(J) cells. In addition to the FACS analysis that showed that the naive HENSM ES cells give rise to around 25% PDGFRA^+^ cells when plated in N2B27 basal conditions (compared with about 65% from RCL-induced cells), we validated by immunostaining the emergence of SOX17^+^ PrE-like and BST2^+^FOXF1^+^ ExEM-like cells in N2B27 basal conditions (Extended Data Fig. 4b). Consistent with the outcome of FACS and immunofluorescence analyses, we could still observe the proper formation of SEMs with the YS and epiblast after omitting the RCL-induced cell fraction, albeit with reduced YS-like morphological quality and a trend towards reduced efficiency (Extended Data Fig. 5c). The latter is consistent with the significantly lower yield (2.5-fold decrease) of PDGFRA^+^ cells from WT naive ES cells when placed in N2B27 compared with RCL pretreatment conditions (Extended Data Fig. 1c). Attempting to generate SEMs by aggregating only HENSM-induced naive ES cells did not produce any organized SEM structures. Instead, we observed disorganized embryonic body (EB)-like structures with dispersed OCT4^+^ ES cells, GATA6^+^ PrE-like and EXEM-like cells and a nearly undetectable GATA3^+^ TE-like fraction (Extended Data Fig. 5d). This result indicates the insufficiency of naive ES cells induced in basal N2B27 conditions to give rise to extra-embryonic-like cells in an optimal frequency for self-organizing into complete embryo-like structures. As we were seeking the highest efficiency possible, we continued with including RCL-primed cells in our SEM aggregation regimen.

## Up to day 14 human SEMs from HENSM ES cells

**Fig. 2:**
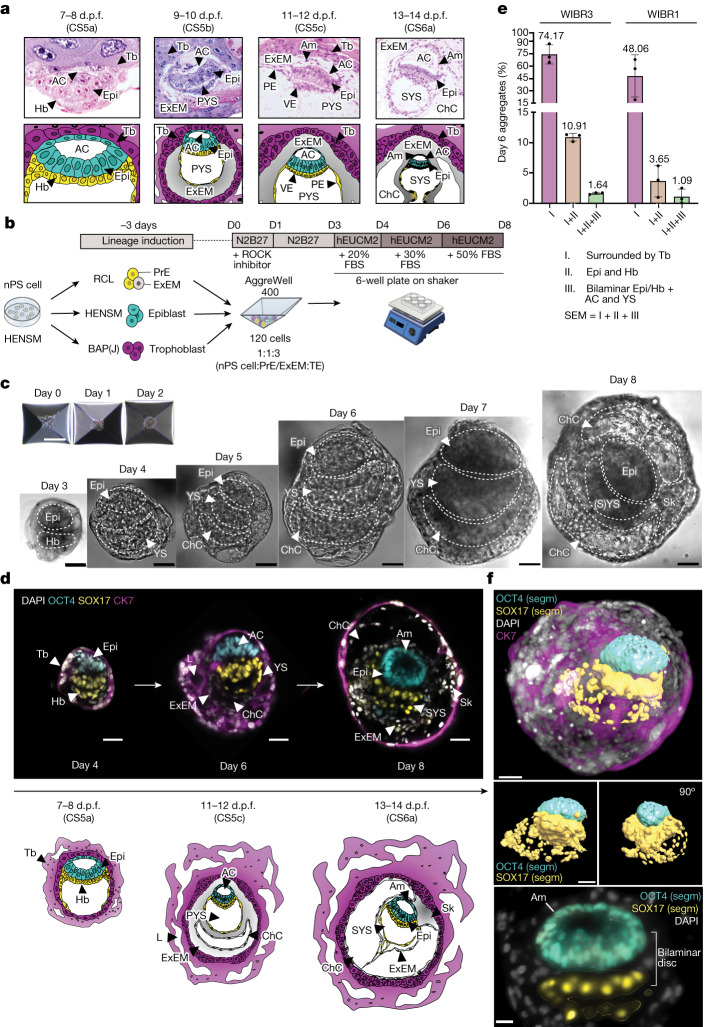
Self-assembly of human post-implantation SEM exclusively from non-transgenic naive ES cells. **a**, Left to right, CS images reproduced from ref. ^20^ (courtesy of the Virtual Human Embryo) and schemes of early post-implantation human embryos at CS5a (7–8 d.p.f.), CS5b (9–10 d.p.f.), CS5c (11–12 d.p.f.) and CS6a (13–14 d.p.f.). **b**, Scheme of the human SEM protocol (Methods). PrE/ExEM-like (yellow/grey), epiblast-like (cyan) and trophoblast-like (magenta) lineage priming for 3 days from nPS cells in HENSM is followed by aggregation (day 0 (D0)) in N2B27. From day 3, SEMs are cultured in non-adherent 6-well plates on an orbital shaker in hEUCM2. **c**, Representative bright-field (BF) images of day 0–8 SEMs showing growth and formation of the embryonic structures, defined by lineage-specific immunofluorescence (Extended Data Fig. 6b). **d**, Right to left, representative immunofluorescence images (top) and schematics (bottom) of SEMs from days 4, 6 and 8 showing OCT4, SOX17, CK7 and nuclei (DAPI). d.p.f. values are approximate. **e**, Quantification of the protocol efficiency for WIBR3 (left) and WIBR1 (right) ES cell lines according to the morphological criteria (Methods). For WIBR3, *N* = 3 across 232, 344 and 344 aggregates; for WIBR1, *N* = 3 across 866, 1,222 and 960 aggregates. Bars show mean values, whiskers mark the s.d. **f**, Top, 3D reconstruction of the day 8 SEM shown in **d** (right) with segmented (segm) epiblast-like and hypoblast/YS-like compartments. Middle, segmentation of the epiblast-like and hypoblast/YS-like compartments shown in 0 and 90° degrees of rotation. Bottom, image section of the day 8 SEM shown in **d** (right). AC, amniotic cavity; Am, amnion; Epi, epiblast; Hb, hypoblast; L, lacunae; Sk, stalk; Tb, trophoblast. Scale bars, 30 µm (**f**, bottom), 50 µm (**c** (days 3–8),**d**,**f** (top and middle)) or 200 µm (**c**, days 0–2). Source Data

**Extended Data Fig. 6:**
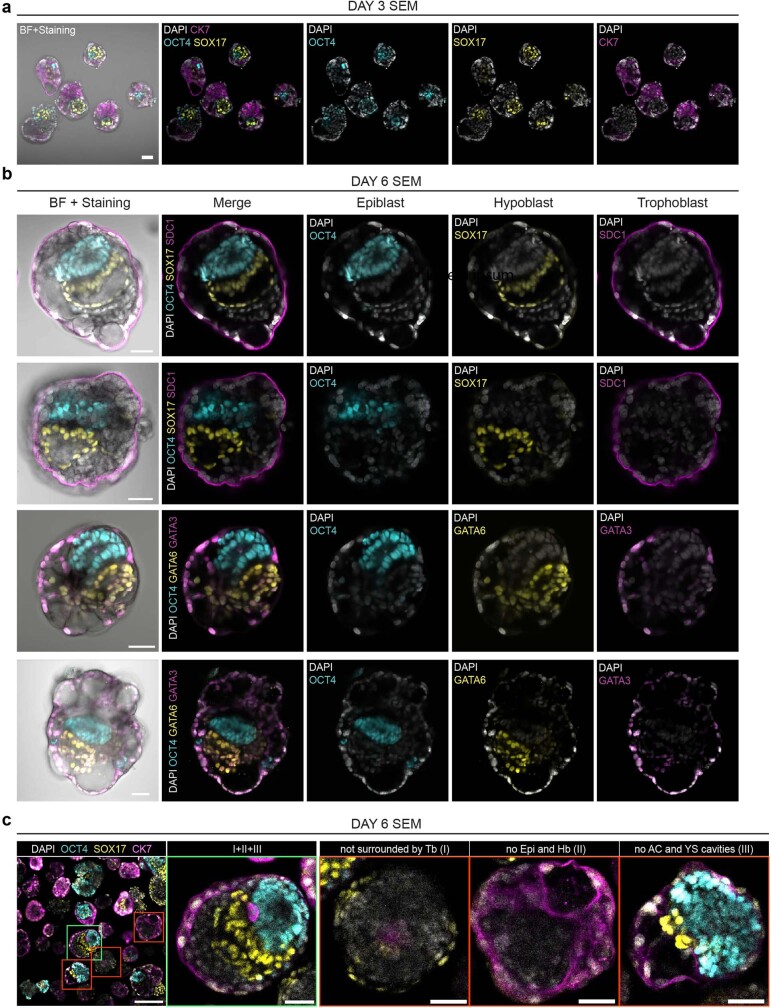
Characterization of epiblast-, hypoblast-, and trophoblast-like lineages in SEMs. **a (left to right)**, representative merged brightfield (BF) and immunofluorescence images of day 3 SEMs showing three lineages, epiblast-like (OCT4, cyan), hypoblast-like (SOX17, yellow), and trophoblast-like lineage (CK7, magenta) merged with a nuclei channel (DAPI, white). **b**, representative merged brightfield and immunofluorescence images of multiple day 6 SEMs, showing epiblast-like (OCT4, cyan), hypoblast-like or hypoblast/ExEM-like (SOX17 or GATA6, respectively, in yellow), and trophoblast-like (SDC1 and GATA3, magenta) compartments merged with a nuclei channel (DAPI, white). **c (left)**, representative immunofluorescence images of day 6 SEMs showing epiblast-like (OCT4, cyan), hypoblast-like (SOX17, yellow), and trophoblast-like (CK7, magenta) compartments; nuclei (DAPI, white). Left panel represents a wide field image with adequate SEMs (outlined in green) and mis-developed structures (outlined in red); scale bar, 200 µm. Right panels zoom into the examples. **c (from left to right**), representative immunofluorescence image of the SEM developed according to the three success rate criteria (outlined in green, see Methods). Representative mis-developed day 6 SEMs (outlined in red) which do not fall within the success rate criteria: not surrounded by trophoblast (Tb)-like cells (I), without epiblast (Epi)- and hypoblast (Hb)- like cells (II), without the amniotic cavity (AC)-like structure and the yolk sac cavity (YS)-like structure and bilaminar Epi/Hb-like structure (III). Scale bars, 50 µm.

We proceeded to test the capacity to form embryo-like structures solely from naive PS cells as a starting population that could mimic different stages of natural human in utero development (Fig. 2a). We calibrated aggregation conditions such as cell numbers needed, ratios within cell mixtures and media compositions for different stages (Supplementary Figs. 8 and 9). The protocol starting with 120 cells per individual aggregate at the ratio 1:1:3 (naive PS cell: PrE/ExEM-like: TE-like) in basal N2B27 conditions supplemented with BSA (which was found to be crucial to reduce human aggregate stickiness) for 3 days (Supplementary Fig. 10a) resulted in optimal aggregation, as validated by the presence of epiblast-like and extra-embryonic-like lineages by immunostaining (Extended Data Fig. 6a). To support growth of the SEM and prevent TE-like cell attachment to the plate surface, which disrupts morphology after day 3, we continued our culture using orbital shaking conditions^3^ (Supplementary Fig. 10b–e). The composition of the human ex utero culture medium 2 (hEUCM2) was adapted from a mouse SEM protocol^3^. Increasing the FBS concentration gradually was optimal for human SEM structural organization (Fig. 2b, Extended Data Fig. 6a,b and Supplementary Figs. 10 and 11).

Throughout the study, we focused on and interchangeably generated SEMs from two human ES cell lines: WIBR3 (46XX) and WIBR1 (46XY). We analysed multiple aspects of SEM structure that produced equivalent SEM structure and morphogenesis results at slightly different efficiencies per cell line (Fig. 2c–e and Extended Data Fig. 6). During 8 days of ex utero culture, the aggregates extensively grew, forming a three-dimensional (3D) spherical structure with evident tissue compartments, self-organization and inner cavity formation (Fig. 2c and Supplementary Videos 1–3). Human SEMs not only expressed the respective lineage markers but also established structures that were morphologically characteristic of in utero-implanted embryos and were in the correct orientation (Fig. 2d,f). From the beginning of the ex utero culture (days 3–4), SEMs became enclosed by the trophoblast-like compartment, marked by GATA3, CK7 and SDC1 (a syncytiotrophoblast marker) (Fig. 2d and Extended Data Fig. 6), which are reminiscent of human early in utero development by 8 days post fertilization (d.p.f.) or Carnegie stage 5 (CS5) (Fig. 2a). At this stage, the implanting embryo starts to become surrounded by an outer layer of syncytiotrophoblasts, which directly invade the maternal endometrium and support future histotrophic nutrition in utero. Notably, the co-aggregation protocol devised here did not result in blastocoel cavity formation or in an inner-cell-mass-like structure, as opposed to blastoids or blastocysts, which indicates that SEMs do not go through a blastocyst-like stage (Supplementary Fig. 12). Furthermore, human blastoids did not develop further in our protocol when cultivated under suspended SEM culture conditions from day 3 onwards (Supplementary Fig. 13).

In humans and nonhuman primates, the inner cell mass segregates into two lineages: the epiblast and the hypoblast. The epiblast is formed by a columnar epithelium layer that expresses OCT4 (refs. ^16,17^), whereas the hypoblast is located underneath the epiblast and comprises cuboidal cells that express SOX17, GATA6, GATA4 and PDGFRA (CS5a; Fig. 2a). Notably, in human SEMs, epiblast-like and PrE-like cells segregated into two distinct compartments, differentially expressing the respective lineage marker genes (*OCT4* and *SOX17*) (Fig. 2d). Both epiblast-like and hypoblast-like compartments were surrounded by the trophoblast-like compartment, marked by CK7 starting from day 3 (Fig. 2d and Extended Data Fig. 6a), and SEMs advanced morphologically by day 6 (Extended Data Fig. 6b). The epiblast-like compartment initiated the formation of the amniotic-like cavity, whereas the hypoblast-like layer formed a YS-like cavity, establishing a bilaminar disc structure in between (Fig. 2d and Extended Data Fig. 6b) reminiscent of the 9–10 d.p.f. human embryo (CS5b; Fig. 2a). The efficiency of forming a correctly organized post-implantation human SEM at day 6 was estimated to be 1.64% for WIBR3 cells and 1.09% for WIBR1 cells of all starting aggregates at day 0, as judged by co-immunofluorescence analyses for lineage markers and morphology criteria (Fig. 2e and Extended Data Fig. 6c). We noted that the human SEMs showed a notable degree of asynchrony within each cell line and within individual experimental batch, with up to 2 days difference in developmental staging for SEMs found at days 6–8, leading to some more advanced and some earlier structures when evaluating SEMs at the same time point. Starting the human SEM protocol with human primed, rather than naive, ES cells did not generate equivalent SEMs (Extended Data Fig. 5e), as also seen in mice^3^.

**Fig. 3:**
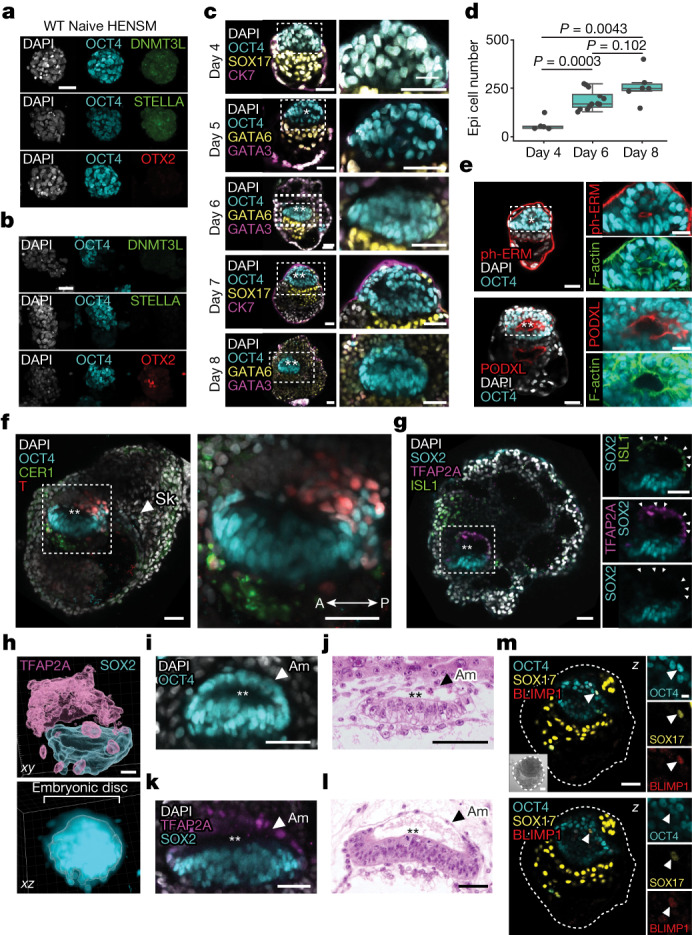
Human SEMs undergo epiblast morphogenesis and form a bilaminar disc-like structure. **a**,**b**, Representative immunofluorescence images of ES cells in HENSM (**a**) and day 3 SEMs in N2B27 (**b**) stained for OCT4, DNMT3L, OTX2 and STELLA. **c**, Left, images of day 4–8 SEMs showing epiblast-like (OCT4), hypoblast-like (SOX17), hypoblast/ExEM-like (GATA6) and trophoblast-like (CK7, SDC1 or GATA3) compartments. Right, zoom-in images. **d**, Epiblast-like cell numbers in successfully developed SEMs from day 4 (*N* = 1, *n* = 5), day 6 (*N* = 3, *n* = 12) and day 8 (*N* = 3, *n* = 6). Whiskers extend 1.5× the interquartile range (box) around the median line. *P* values, two-sided Mann–Whitney *U*-test. **e**, Images of day 6 SEMs showing phospho-ezrin, radixin and moesin (ph-ERM; top), podocalyxin (PODXL; bottom) and F-actin**. f**, Image of a day 8 SEM with the anterior–posterior (A–P) axis with T/BRA (red) in epiblast-like compartment (OCT4) opposite to CER1^+^ AVE in the anterior Hb. Right, zoom-in image. **g**, Image of a day 8 SEM with TFAP2A^+^, ISL1^+^ and SOX2^**–**^ amnion-like cells (arrowheads on the right). **h**, 3D segmentation of the amnion-like (TFAP2A) and the embryonic disc-like (SOX2) compartments in day 8 SEM. *xy* and *xz* views. **i**, Image of an amnion-like structure in a day 7 SEM with squamous OCT4^+^ cells. **j**,**l**, CS5c (**j**) and CS6a (**l**) histological sections reproduced from ref. ^20^. Amnion-like cells indicated by arrowheads. **k**, Image of squamous TFAP2A^+^ and SOX2^−^ amnion-like cells in day 8 SEMs. **m**, Images of day 8 SEMs (*z* slices *n* = 22 (top) and 26 (bottom)). Right, zoom into the OCT4^+^SOX17^+^BLIMP1^+^ PGC-like cells (arrowheads). Inset, BF image. The SEM perimeter is outlined. Single and double asterisks mark proamniotic-like and amniotic-like cavities, respectively. Nuclei were stained using DAPI. Scale bars, 10 µm (**m**, right), 25 µm (**e**, right), 20 µm (**h**) or 50 µm (all other images). Source Data

**Extended Data Fig. 7:**
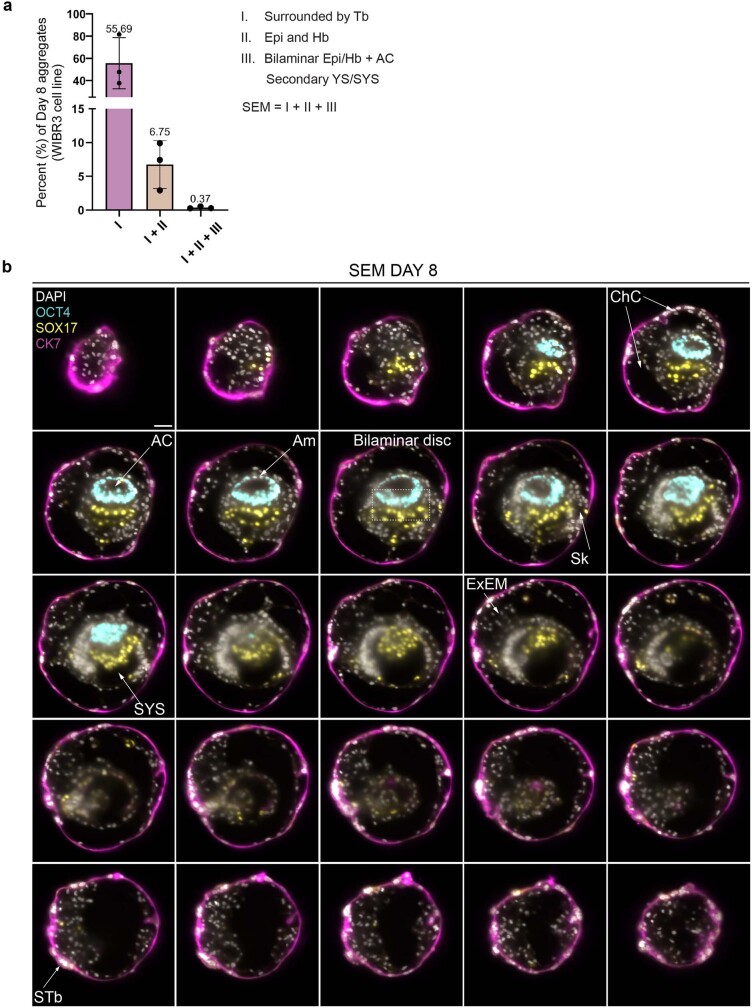
Analyzing 3D structure of the human SEM at day 8. **a**, quantification of the SEM derivation efficiency at day 8 for WIBR3 line according to the criteria: I, surrounding by Tb-like layer; II, presence of Epi-like and Hb-like compartments; III, presence of Bilaminar Epi/Hb disk-like structure, AC-like structure, and YS-like or Secondary(S) YS-like structures. N = 3 across 1251, 377, and 700 SEM aggregates. Bars show mean values, whiskers mark s.d. **b**, individual Z-planes of the 3D immunofluorescence image of a day 8 human SEM that meets criteria III specified in **a**. Epiblast-like (OCT4, cyan), hypoblast-like (SOX17, yellow), and trophoblast-like (CK7, magenta) compartments; nuclei (DAPI, white). AC, amniotic cavity-like; Am, amnion-like; ExEM, extraembryonic mesoderm-like; SYS, secondary yolk sac-like; ChC, chorionic cavity-like; STb, syncytiotrophoblast-like; Sk, stalk-like. Z-step, 20 µm; scale bar, 50 µm. See also Supplementary Video 1. Source Data

The early post-implantation pre-gastrulating human embryo already contains ExEM^18^, which contributes to the remodelling of the ChC; this results in formation of the connecting stalk and participates in the formation of blood and placental vasculature by filling the chorionic villi^19^. ExEM tissue becomes abundant between the primary yolk sac (PYS) and the trophoblast by CS5a, forming a ChC underneath the PYS by CS5c (11 d.p.f.; Fig. 2d). The latter was observed after close examination of day 6–8 SEMs, which revealed the presence of the cavity formed by an additional tissue layer between the YS-like and the trophoblast-like compartments, corresponding to a ChC-like structure, consistent with what has been characterized in natural human embryos corresponding to these stages (Fig. 2c,d). Later, the ExEM expands to allow the remodelling of the PYS into the secondary yolk sac (SYS) and the formation of a connecting stalk, the structure that crosses through the ChC and holds the bilaminar disc to the chorion, which later contributes to the umbilical cord^19^. In human day 8 SEMs, we also observed 3D expansion of all the above-mentioned lumina-like structures and growth of extra-embryonic-like tissues **(**0.42% efficiency of all starting day 0 aggregates; Extended Data Fig. 7a). This result indicates the differentiation and remodelling of the PYS-like compartment into the SYS-like compartment alongside ExEM-like compartment expansion and the formation of a stalk-like structure (Figs. 2c,d and 3f, Extended Data Fig. 7b and Supplementary Video 1).

By 11–12 d.p.f. (CS5c) in human embryos, the embryonic disc segregates into a ventral pseudostratified epiblast and a dorsal squamous amnion (Fig. 2a). The bilaminar epiblast will give rise to the embryo proper, whereas the amnion will constitute the protective membrane surrounding the fetus until birth. Starting from day 6 until day 8 of the ex utero culture, the dorsal segment of the epiblast-like compartment acquired squamous morphology resembling the amnion-like layer, and the bilaminar structure adapted a disc shape (Fig. 2d,f, Extended Data Fig. 7b and Supplementary Videos 1–3), thereby resembling a key hallmark of the in utero human post-implantation development in preparation for gastrulation. Altogether, our approach exploits the developmental plasticity of genetically unmodified transgene-free human HENSM naive ES cells and demonstrates their distinct capacity to self-assemble into early post-implantation human embryo models that comprise both embryonic and extra-embryonic compartments.

## SEMs with a bilaminar-disc-like structure

**Extended Data Fig. 8:**
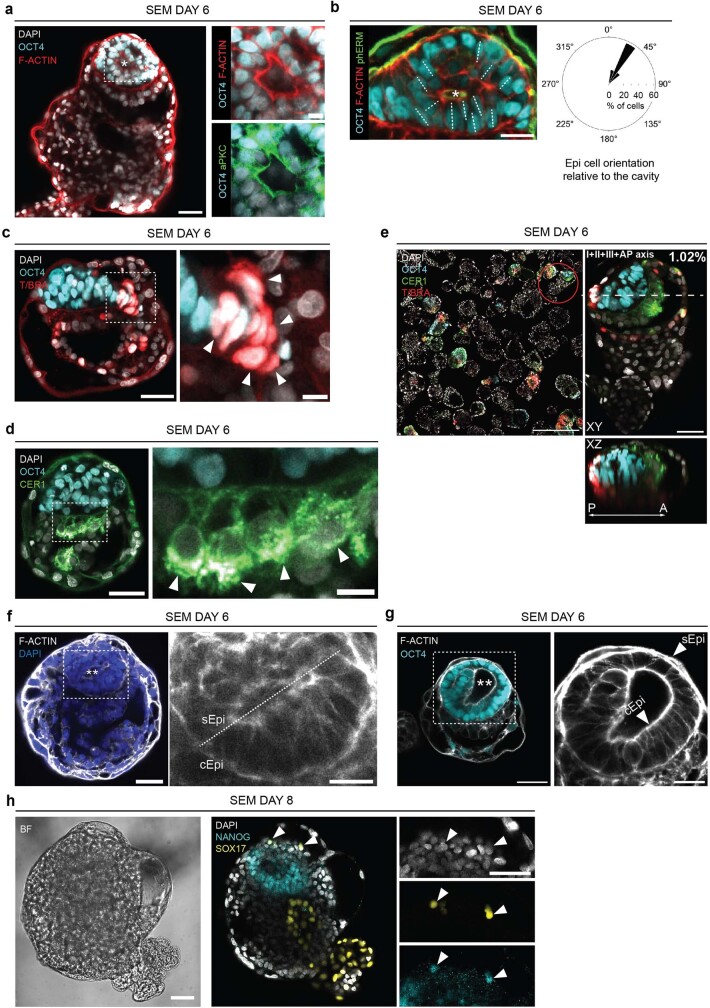
Characterization of the epiblast-like structure in human SEM. **a**, representative immunofluorescence image of day 6 SEM showing aPKC (green), OCT4 (cyan), F-ACTIN (red), nuclei (DAPI, white). Scale bar, 50 µm; 12.5 µm (zoom, right). **b (left)**, immunofluorescence image of day 6 SEM showing OCT4 (cyan), F-ACTIN (red), and phERM (green); alignment of Epi-like cells in a single 2D plane is marked with dashed lines; asterisk, pro-amniotic-like cavity. Scale bar, 25 µm. **b** (**right)**, quantification of the angle between the epiblast-like cell axis and the pro-amniotic-like cavity; the plot shows the radial histogram of the angle values and indicates predominant alignment of epi-like cells towards the center of the emerging cavity (n = 32). **c**, representative immunofluorescence image of day 6 SEM showing T/BRA expression (red); OCT4 (cyan), nuclei (DAPI, white). Right, zoom into the posterior epiblast-like structure, arrows mark individual T/BRA-positive cells. Scale bar, 50 µm, 12.5 µm (4x zoom in, right). **d**, representative immunofluorescence image of day 6 SEM showing CER1 (green) localization inside the intracellular vesicles; OCT4 (cyan), nuclei (DAPI, white). Right, zoom into hypoblast-like compartment, arrows mark apical side of the visceral endoderm-like cells with CER1 vesicles. Scale bar, 50 µm, 12.5 µm (4x zoom, right). **e (left)**, immunofluorescence image of day 6 aggregates showing T (red), OCT4 (cyan), CER1 (green), and nuclei (DAPI, white); scale bar, 500 µm. **e (right)**, zoom into the SEM with expression of T in epiblast-like compartment and CER1 in hypoblast-like compartment at the opposite sides (defined as AP-axis, see XZ cross section below) observed in 1.02% of total number of starting aggregates on day 0. N = 2 across 1251 and 700 aggregates. Scale bar, 50 µm. **f**, representative immunofluorescence image of day 6 SEM showing F-ACTIN (white) and nuclei (DAPI, blue). Right, zoom into epiblast-like structure with squamous cells (sEpi) in the top and cylindrical/columnar cells (cEpi) in the bottom parts of the epiblast-like compartment. Scale bar, 50 µm; zoom, 25 µm. **g**, representative immunofluorescence image of day 6 SEM showing F-ACTIN (white) and OCT4 (cyan). Right, zoom into epiblast-like compartment with squamous cells in the top and cylindrical cells in the bottom parts of the epiblast-like compartment. Scale bar, 50 µm; zoom, 25 µm. **h**, representative brightfield and immunofluorescence images of day 8 SEM showing NANOG (cyan), SOX17 (yellow), and nuclei (DAPI, white). Right, zoom into PGC-like cells in day 8 human SEMs, co-expressing NANOG and SOX17 (cells marked with arrows). Scale bars, 50 µm. Source Data

We aimed to characterize the development of key lineages in SEMs in more detail. As the epiblast is derived from initially naive ES cells, we checked whether they undergo priming after aggregation with other lineages in aggregation conditions. Shortly after co-aggregation, we noted loss of expression of naive markers (DNMT3 and STELLA) and upregulation of the primed pluripotency marker OTX2 by day 3 (Fig. 3a,b). Consistent with developmental progression, by day 4, ES cells formed an evident epiblast-like tissue inside the SEM, which grew considerably during subsequent development (Fig. 3c and Supplementary Videos 1–4). The epiblast-like compartment in human SEMs showed cell polarization and lumenogenesis from day 6, as judged by the apical localization of phosphorylated erzin, podocalyxin (Fig. 3e) and aPKC (Extended Data Fig. 8a), as well as alignment of the epiblast-like cells towards the emerging cavity (Extended Data Fig. 8b). The timing of lumenogenesis at day 6 also corresponded to a significant increase in epiblast-like cell numbers (Fig. 3d), in agreement with histological descriptions of the in utero human embryo^20^.

Early emergence of the anterior–posterior axis is prevalent in mammals when a proportion of epiblast cells initiates T expression at the prospective posterior side of the embryonic disc. Therefore, we checked for the expression of T in human SEMs and identified a T^+^ population of epiblast-like cells that marked the posterior part of the SEM epiblast (Fig. 3f and Extended Data Fig. 8c). In parallel, the emergence of the anterior visceral endoderm (AVE)-like compartment, which constitutes the anterior signalling centre for epiblast patterning, was seen by the expression of CER1 in the epiblast-adjacent part of the visceral endoderm (VE) from day 6 (Extended Data Fig. 8d). The vesicular localization of CER1 was evident in human SEMs (Extended Data Fig. 8d). Co-immunostaining for these markers further supported the establishment of the anterior–posterior axis and symmetry breaking starting from day 6, with an efficiency of 1.02% (Extended Data Fig. 8e), at which T^+^ epiblast-like cells could be found in the region opposite to CER1^+^ AVE-like cells in the Hb (Fig. 3f).

From day 6 onwards, the epiblast-like compartment exhibited patterning of the early amniotic sac-like structure, with a dorsal squamous cell population resembling the putative amnion and ventral columnar pseudostratified epiblast-like cells (Extended Data Fig. 8f,g). Immunostaining for TFAP2A and ISL1 in SEMs revealed their co-localization in multiple squamous dorsal cells, also depleted of SOX2 expression, which confirmed their amnion-like identity by localization, morphology and gene expression^21^ (Fig. 3g and Supplementary Video 5). Based on cell morphology and localization, the polarized amnion can be distinguished from the rest of the epiblast between 10 and 12 d.p.f. of human in utero development^20^. Moreover, by day 8 in SEMs, the epiblast-like structure acquired an apparent disc shape (Fig. 3h) with an enlarged amniotic-like cavity, whereas the amnion formed a thinner squamous-shaped epithelium highly resembling in utero embryo morphology as documented in the Carnegie collection at CS6a (Fig. 3h–l and Supplementary Video 5). Last, we asked whether advanced in utero-like development of the embryonic disc would also lead to the induction of early primordial germ cell (PGC)-like cells, as was seen in mouse SEMs derived from naive ES cells^3^. In some day 8 SEMs, co-immunostaining for several PGC markers identified a population of PGC-like cells positive for OCT4, SOX17 and BLIMP1 (Fig. 3m and Extended Data Fig. 8h).

## YS-like and ChC-like structures

**Fig. 4:**
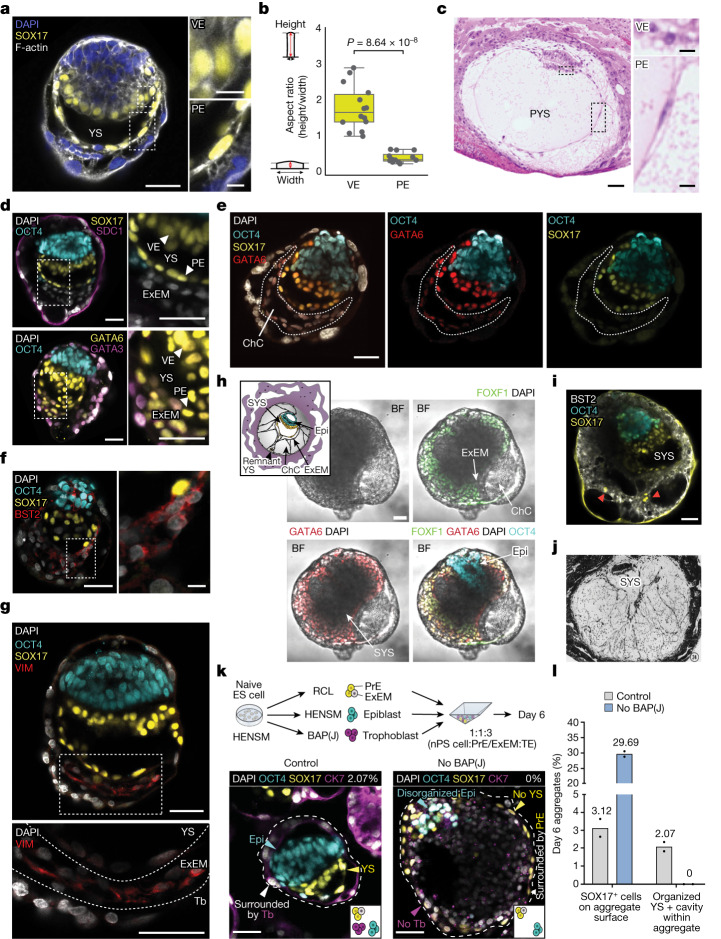
Human SEMs recapitulate YS-like lumenogenesis and SEM scaffolding by ExEM-like cells. **a**, Representative immunofluorescence image of a day 6 SEM showing YS-like, PE-like and VE-like (SOX17) compartments, F-actin and nuclei (DAPI). Right, zoom into VE-like and PE-like cells. **b**, Cell aspect ratio in VE-like (*n* = 14) and PE-like cells (*n* = 12) of the SEM in **a**. Whiskers extend 1.5× of the interquartile range (box) around the median line. Two-sided Student’s *t*-test. **c**, CS5c histological sections reproduced from ref. ^20^ showing PYS-like (left) and VE-like and PE-like compartments (right). **d**, Images of day 6 SEMs showing epiblast-like (OCT4^+^), hypoblast-like (SOX17^+^), hypoblast/ExEM-like (GATA6^+^) and trophoblast-like (CK7^+^ and GATA3^+^) compartments. Right, ×2 zoom images of ExEM-like cells. **e**, Image of a day 6 SEM showing a ChC-like structure within GATA6^+^SOX17^–^ ExEM-like tissue (outlined). **f**, Image of a day 6 SEM expressing BST2^+^ underneath a SOX17^+^ YS-like structure. Right, zoom-in image. **g**, Image of a day 8 SEM expressing VIM underneath SOX17^+^ YS-like cells (yellow). Bottom, zoom-in image. **h**, Inset shows a schematic of the ExEM cells (grey) and SYS (yellow) in 14 d.p.f. human embryo. BF images are of day 8 SEMs showing FOXF1^+^GATA6^+^ ExEM-like cells. **i**, Image of a day 8 SEM with a cavitated BST2^+^ ExEM-like cell and SOX17. Red arrowheads indicate PYS remnant-like cells. **j**, CS6 histological section reproduced from ref. ^18^ showing filamentous ExEM and SYS (×100 magnification). **k**, Top, scheme of the regular aggregation experiment (control) and without trophoblast-like cells (no BAP(J)). Bottom, images of control SEM and no BAP(J) aggregate from day 6 stained for SOX17 and CK7. **l**, Quantification of day 6 aggregates in control (*N* = 2, *n* = 344 and 1,321) and no BAP(J) (*N* = 2, *n* = 472 and 193) conditions. Bars show mean values from two biological replicates, and each dot indicates an average value of a biological replicate. Scale bars, 10 µm (zoom-in images in **a**,**c**,**f**) or 50 µm (all other images). Source Data

**Extended Data Fig. 9:**
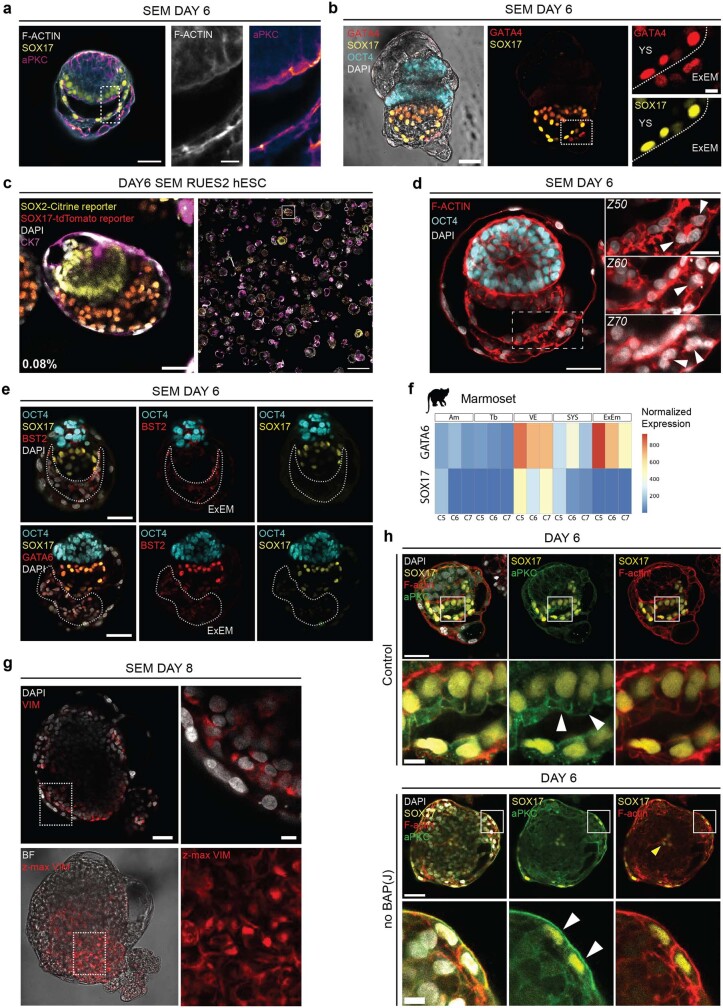
Characterization of hypoblast-lake layer and extraembryonic mesoderm-like cells in human SEMs. **a**, representative immunofluorescence image of day 6 SEM showing apical polarity of the visceral and parietal hypoblast-like layers (SOX17, yellow); aPKC (heat gradient), F-ACTIN (white). Scale bar, 50 µm; zoom, 10 µm. See also Supplementary Video 6. **b**, representative immunofluorescence image of day 6 SEM showing OCT4 (cyan), GATA4 (red), SOX17 (yellow), and nuclei (DAPI). Right, zoom on ExEM-like cells expressing GATA4, but not SOX17. YS, yolk sac-like. Scale bar, 50 µm; zoom, 10 µm. **c (left)**, zoom into the SEM example with adequate morphology and structure (see Methods) aggregated from RUES2 reporter hESC line showing epiblast-like (SOX2-Citrine, yellow), hypoblast-like (SOX17-tdTomato, red), and trophoblast-like (CK7, magenta) compartments with 0.08% efficiency (N = 3 across 409, 295, and 528 aggregates); nuclei (DAPI, white); scale bar, 50 µm. **c (right)**, representative large field of view immunofluorescence image of day 6 SEM aggregates, the zoom on the left is outlined; scale bar, 500 µm. **d**, representative immunofluorescence image of day 6 SEM showing mesenchymal-like cells underneath the yolk sac-like structure; OCT4 (cyan), F-ACTIN (red), and nuclei (DAPI, white). Right, zoom into the region underneath the yolk sac-like structure, shown in different Z-planes (number 50, 60, and 70). Arrows point at the cells between yolk sac-like and the trophoblast-like compartments. Scale bar, 50 µm; zoom, 25 µm. See also Supplementary Video 4. **e**, representative immunofluorescence images of day 6 SEMs showing chorionic-like cavity surrounded by ExEM-like cells (outlined), negative for SOX17 (yellow), but expressing BST2 (top, red) and GATA6 (bottom, red); OCT4 (cyan), nuclei (DAPI, white). Scale bar, 50 µm. **f**, heatmap of GATA6 and SOX17 gene expression across extraembryonic tissues in marmoset, corresponding to the Carnegie stages (CS) 5-7), extracted from previously published gene expression dataset^8^. Am, amnion; Tb, trophoblast; VE, visceral endoderm; SYS, secondary yolk sac; ExEM, extraembryonic mesoderm. **g (top)**, representative immunofluorescence image of Z slice from day 8 SEM and the zoom into the ExEM-like region showing VIM (red) expression and nuclei (DAPI, white). **g (bottom)**, merged brightfield and maximum intensity projection showing VIM (red) expression. Scale bar, 50 µm; zoom, 10 µm. **h (top)**, representative immunofluorescence image of day 6 SEM from aggregation with trophoblast-like cells (control). The arrows in the zoom below point at the apical surface of the visceral hypoblast-like layer (SOX17, yellow); aPKC (green), F-ACTIN (red). Scale bar, 50 µm; zoom, 10 µm. **h (bottom)**, representative immunofluorescence image of day 6 aggregate from experiment where trophoblast-like cell fraction was omitted (no BAP(J)) condition (related to Fig. 4k). The arrows in the zoom below point at the apical surface of the PrE/hypoblast-like cells (or layer) surrounding the aggregate (SOX17, yellow); aPKC (green), F-ACTIN (red). Scale bar, 50 µm; zoom, 10 µm.

The YS starts forming from the hypoblast between CS4 and CS5 in humans. The part of the hypoblast located underneath the epiblast (together forming the characteristic bilaminar disc) belongs to the VE and is a dynamic signalling centre for epiblast patterning. It is connected to the parietal endoderm (PE), which forms the inner cavity called the PYS, which becomes reorganized during development^18^. Eventually, the YS serves multiple functions for the growing embryo, supplying nutrients and maintaining blood cell progenitors during the embryonic period until the placenta takes over^22^. Formation of the YS cavity-like structure was frequently seen in SEMs with all segregated lineages and became more prominent at day 6 of ex utero development (Fig. 4). Once formed, the SOX17^+^ YS-like compartment comprised columnar VE-like cells in proximity of the epiblast-like layer and squamous PE-like cells lining the opposing side of the cavity (Fig. 4a–c), which resembled VE and PE cell morphology in the PYS of CS5c natural embryos (Fig. 4c). The latter was uniformly observed in SEMs with YS-like compartment formation. Both VE-like and PE-like cells acquired apicobasal polarity, as judged by the apical localization of aPKC (Extended Data Fig. 9a and Supplementary Video 6), which was in agreement with hypoblast cell morphology in equivalent developmental-stage rhesus monkey embryos^23^. Consistent with polarity, cell shape and localization in natural embryos, the SOX17^+^ hypoblast-like compartment always co-expressed the primitive endoderm markers GATA6 and GATA4 (Fig. 4d,e and Extended Data Fig. 9b). Testing of the SEM organization was also done using a SOX17–tdTomato; SOX2–Citrine RUES2 reporter human ES cell line, which enabled the detection of the SOX17^+^ YS-like and the SOX2^+^ epiblast-like compartments. This experiment confirmed the formation of structured SEMs from a third independently generated naive ES cell line, albeit with reduced (0.08%) efficiency (Extended Data Fig. 9c).

We characterized the above-mentioned OCT4^−^ and SOX17^−^ cell population beneath the YS-like compartment (Fig. 2d). These cells had mesenchymal rather than epithelial morphology, extending cell protrusions towards the surrounding tissues and forming an intermediate mesh-like 3D structure (Extended Data Fig. 9d). We then checked the expression of multiple lineage-specific transcription factors, which differentially marked the ExEM compared with the YS during the relevant stages in marmoset embryos^8^. The ExEM-like cells expressed GATA6 and GATA4, but not SOX17 (Fig. 4e and Extended Data Fig. 9e), a result consistent with the ExEM expression profile from marmoset embryos^8^ (Extended Data Fig. 9f) and from human naive ES cell in vitro-derived ExEM cells^9^. The observed lower GATA6 level in ExEM-like cells compared with PrE-like cells (Fig. 4e and Extended Data Fig. 9e) was also in agreement with the expression for those lineages in the in utero marmoset embryos^8^ (Extended Data Fig. 9f). Immunostaining for additional mesenchymal markers, such as BST2 (ref. ^9^), VIM^16^ and FOXF1, which can help distinguish the ExEM from the PrE, further validated the ExEM-like identity of these cells in day 6–8 SEMs (Fig. 4f,g, Extended Data Fig. 9g and Supplementary Video 7). Hence, we concluded that the inner cavity of the ExEM-like cells represents the ChC-like structure, which is formed by the remodelling of a mesh-like population of mesenchymal cells predominantly visible starting from as early as day 6 in SEMs, that corresponds to the human ExEM.

Histological descriptions of human embryos suggest that there is remodelling of the PYS cavity by ExEM expansion, with subsequent pinching-off and vesiculation of PYS remnants that results in the formation of the SYS^18^. In some of the day 8 SEMs, BST2^+^ and FOXF1^+^ cells formed a complex filamentous meshwork with multiple cavities that contributed to the complex architecture of the SEM (Fig. 4h,i and Supplementary Fig. 14). Closer examination of these structures revealed clusters of SOX17^+^ cells entrapped between ExEM-like cells, which suggested the presence of residual PYS-like cells after tissue remodelling (Fig. 4i,j and Supplementary Fig. 14). Moreover, an ExEM-like cell population connecting the bilaminar disc to the trophectoderm could be seen in day 8 SEMs that resembled a connecting stalk structure (Fig. 2d and Extended Data Fig. 7b).

Following deeper analysis of the structure patterns obtained in lineage omission experiments (Extended Data Fig. 5a–c), we noted that when BAP(J)-induced cells were omitted from the initial aggregation mixture, this abolished internal structure and cavity formation, including YS-like compartment formation and patterning and self-organization of the bilaminar disc (Fig. 4k), and aberrant expansion of an OCT4^−^SOX17^−^CK7^−^ cell population that corresponded to ExEM-like cells included in aggregates (Extended Data Fig. 2). Specifically, in the absence of BAP(J) cell input, SOX17^+^ cells fully surrounded the aggregates, which was in contrast to control SEMs surrounded by a trophoblast-like compartment and had the ability to form a SOX17^+^ inner YS-like and bilaminar disc-like structures (Fig. 4k,l). Consistently, in positive control SEM aggregation settings conducted throughout this study, we never observed correct YS-like compartment formation in the absence of a surrounding trophoblast-like layer (Extended Data Fig. 9h and Fig. 4k, l). These results provide evidence in the human context that the trophoblast compartment functionally influences the organization of the epiblast-like and YS-like compartments and cavity formation, at least in the context of this specific human embryo model regimen. It will be of future interest to decipher the mechanistic basis for this effect, which could be signalling based and/or influenced by physical cues dictated by the confining trophoblast compartment.

## Trophoblast-like cell maturation in SEMs

**Fig. 5:**
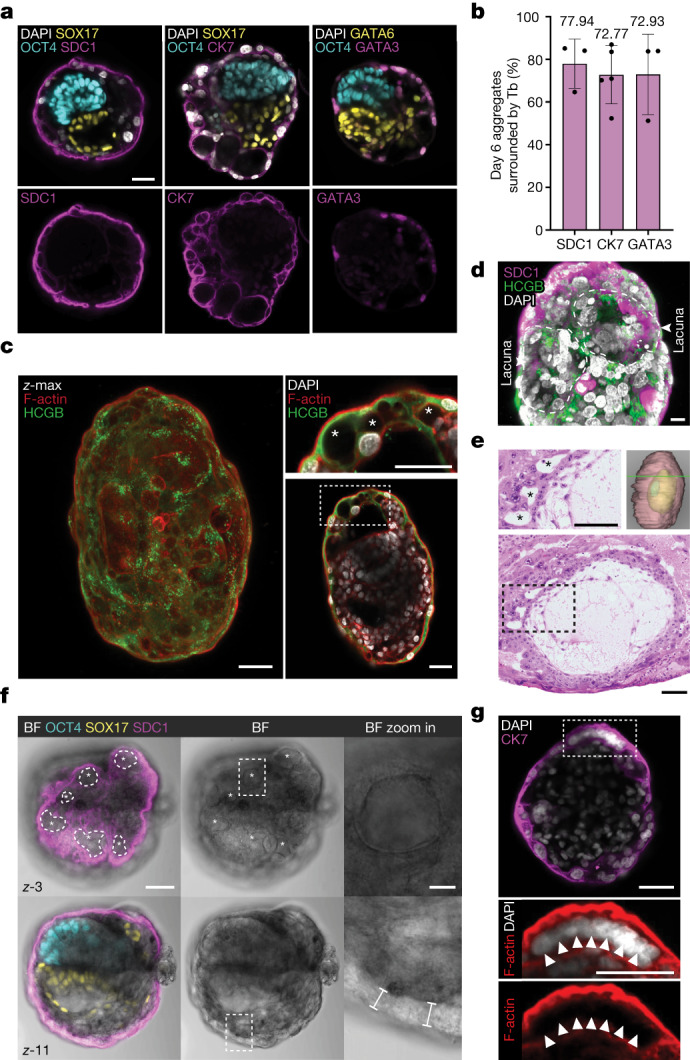
Trophoblast-like compartment integration and maturation in human SEMs. **a**, Top, representative immunofluorescence images of day 6 SEMs showing epiblast-like (OCT4), hypoblast/YS-like (SOX17 or GATA6), ExEM (GATA6) and trophoblast-like (SDC1, CK7 or GATA3) compartments. Bottom, single-channel images of the trophoblast-like compartment surrounding the SEMs. **b**, Average percentage of aggregates surrounded by a trophoblast-like compartment at day 6, as judged by the expression of SDC1, CK7 or GATA3. SDC1, *N* = 3 across 533, 232 and 94 aggregates; CK7, *N* = 5 across 302, 153, 344, 344 and 85 aggregates; and GATA3, *N* = 3 across 295, 170 and 62 aggregates. Error bars indicate the s.d. **c**, Left, *z* maximum intensity projection (*z* max) image of day 6 SEMs showing HCGB expression in the outer cells. Right, image of the same SEM showing lacunae-like structures (marked with asterisks) inside the outer syncytiotrophoblast-like layer. **d**, 3D projection of the lacunar-like structures (outlined) in the trophoblast-like layer of a day 6 SEM shown in **c**. Immunofluorescence for SDC1, HCGB and nuclei (DAPI). **e**, Histological section and 3D reconstruction (top right) reproduced from the Carnegie collection of a human embryo^20^ at CS5c showing lacunae in the syncytiotrophoblast (asterisks or box with dotted outline). **f**, Representative BF and immunofluorescence images of two different *z* planes (number 3 and 11, top and bottom, respectively) of day 6 SEMs showing epiblast-like (OCT4), hypoblast-like (SOX17) and trophoblast-like (SDC1) compartments. Top left, lacunae-like structures are outlined and marked with asterisks. Top right, zoom into the lacunae-like structure (top). Bottom, zoom into the outer syncytiotrophoblast-like layer. Brackets mark the thickness of syncytium-like tissue. **g**, Top, image of a day 6 SEM showing CK7, F-actin and nuclei (DAPI). Bottom, zoom into the multinucleated syncytiotrophoblast-like cell. Arrowheads indicate multiple nuclei inside the single cell. Scale bars, 10 µm (**f**, top zoom-in), 20 µm (**d**) or 50 µm (all other images). Source Data

**Extended Data Fig. 10:**
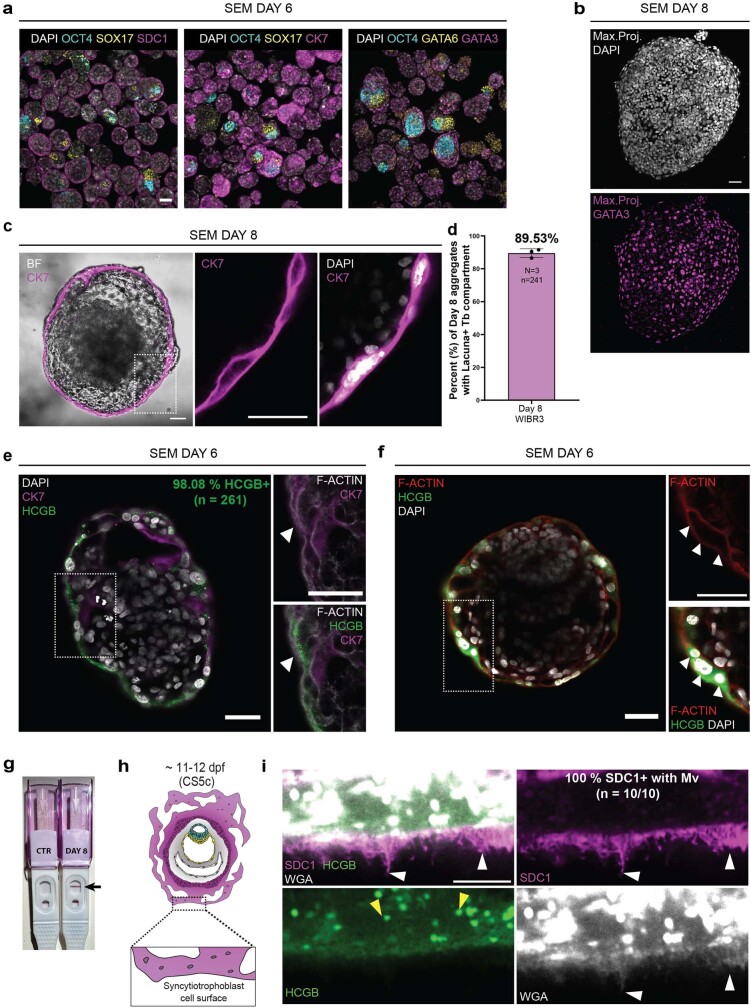
Characterization of the trophoblast-like compartment in human SEM. **a**, representative immunofluorescence images of multiple day 6 SEMs showing epiblast-like structure (OCT4, cyan), hypoblast-like structure (SOX17 and GATA6, yellow), and trophoblast-like layer (SDC1 and GATA3, magenta) surrounding the SEMs; nuclei (DAPI, white), that were used for calculation presented in (Fig. 5b). Scale bar, 200 µm. **b**, maximum intensity projection (Max. Proj.) of the immunofluorescence image of day 8 SEM showing trophoblast-like cells (GATA3, magenta) and nuclei, (DAPI, white). Scale bar, 50 µm. **c**, representative brightfield image overlayed with the immunofluorescence of day 8 SEM showing the outer trophoblast-like compartment (CK7, magenta) surrounding the entire SEM. Right, zoom into the multinucleated trophoblast-like layer. Scale bars, 50 µm. **d**, quantification of the percentage of lacunae-like positive aggregates in all Tb-like positive aggregates (N = 3 independent experimental replicates, n = 241). Bars show mean values, whiskers mark s.d. **e**, representative immunofluorescence image of day 6 SEM showing HCGB (green) and CK7 (magenta). Right, zoom into HCGB+ syncytiotrophoblast-like cells; nuclei (DAPI, white), F-ACTIN (white, right zoom). HCGB expression was detected in 98.08% of all of aggregates with Tb-like compartment (n = 261). Arrows point at the outer syncytiotrophoblast-like cell surface. Scale bars, 50 µm. **f**, representative immunofluorescence image of a human SEM showing multinucleated HCGB-positive syncytiotrophoblast-like cells; HCGB (green), F-ACTIN (red), nuclei (DAPI, white). Arrows point at multiple nuclei inside the same single cell. Scale bars, 50 µm. **g**, commercial ELISA pregnancy test run on spent medium of the day 8 SEMs (Day 8, right) compared to unspent medium as a negative control (CTR, left) which detects the secretion of HCGB from the syncytiotrophoblast-like compartment of day 7-8 human. **h**, scheme of the 11-12 dpf human embryo with the zoom into the surface of the syncytiotrophoblast (magenta). **i**, representative immunofluorescence images of the syncytiotrophoblast-like cell surface in day 8 SEM showing SDC1 (magenta), HCGB (green), and the lipid membrane (WGA, white). Yellow and white arrows point at the HCGB+ vesicles and microvilli-like (Mv) plasma membrane protrusions, respectively. Mv-like protrusions were observed in 10 out of 10 SEMs surrounded by Tb-like compartment that expresses SDC1 (selected randomly for high-resolution imaging). Scale bar, 10 µm. Source Data

In utero, the human embryo develops surrounded by the trophoblast layer, which is essential for truly integrated experimental models of early post-implantation development. Immunofluorescence analysis showed that the majority of SEM aggregates were surrounded by trophoblast-like cells, with an efficiency of 48–74%, and expressed multiple trophoblast markers such as GATA3, CK7 and SDC1 (ref. ^14^) (Fig. 5a,b and Extended Data Fig. 10a, b). Marker expression and cell morphology further indicated that the outer-most trophoblast-like layer was formed by syncytiotrophoblast-like cells, thereby confirming the development of the post-implantation trophoblast in human SEMs (Fig. 5a and Extended Data Fig. 10a–c). Notably, SDC1 was not expressed on the starting TE-like cells following BAP(J) induction before the aggregation step, which indicated that maturation of the TE-like cells occurs in the aggregates (Extended Data Fig. 3d).

The lacunar phase of trophoblast development begins after implantation, when the fluid-filled spaces from within the trophoblast syncytium merge and partition the trophoblast into trabeculae^24^ that later contribute to the placental villi. In around 90% of aggregates surrounded by the trophoblast-like compartment, we observed multiple cavities with variable sizes forming inside the syncytiotrophoblast-like layer, which were predominantly located at the SEM periphery (Fig. 5c,d, Extended Data Fig. 10d and Supplementary Video 8) and resembled the trophoblast lacunae on the embryo periphery in utero at CS5c (Fig. 5e). Immunostaining for human chorionic gonadotropin-β (HCGB) demonstrated abundance of the hormone protein in surrounding syncytiotrophoblast-like cells of the SEM and enriched in the intracellular vesicles^14^ (Fig. 5c and Extended Data Fig. 10e,f,i). Its secretion was confirmed by detection of soluble HCG in medium in which SEMs were cultured from day 7 to day 8 (Extended Data Fig. 10g).

Examination of the trophoblast syncytium-like layer in SEMs revealed microvilli-like structures on the plasma membrane of syncytiotrophoblast-like cells in all validated SEMs analysed (Extended Data Fig. 10h,i), similar to the placental syncytium in utero^24^. Last, we checked whether the syncytium in SEMs is multinuclear, as is typical during early placental development. Phase images alongside co-immunostaining of the trophoblast marker SDC1 enabled us to see more clearly the trophoblast cell shape forming a layer of thick outer syncytium (Fig. 5f). Co-immunostaining with F-actin, which helps define individual cell membrane boundaries, and DAPI validated that the trophoblast-like cells have multiple nuclei (Fig. 5g and Extended Data Fig. 10f).

## scRNA-seq analysis validates human SEMs

**Fig. 6:**
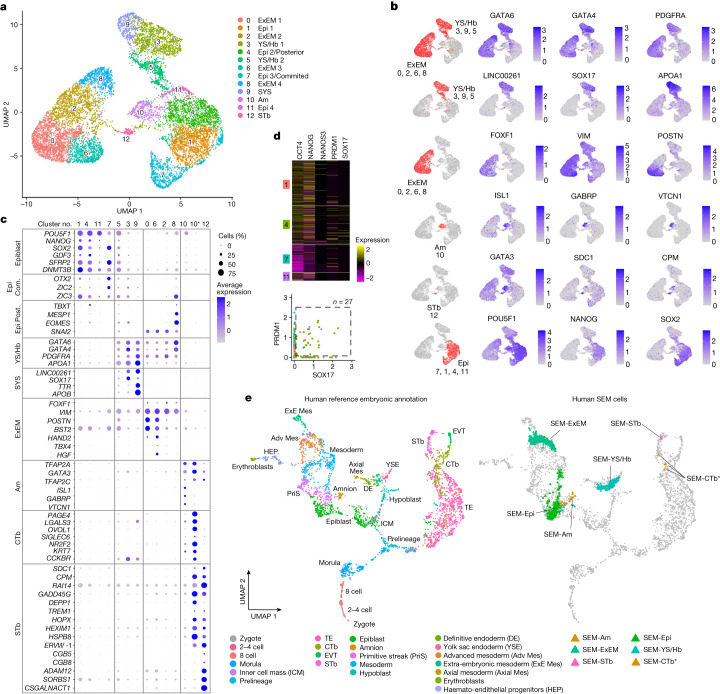
scRNA-seq analysis validates key cell-type identities constituting the human SEM. **a**, UMAP of single cells from human SEMs coloured by the cell clusters and identified on the basis of known marker genes and gene signatures (Methods). Cluster sizes: 1,963 (cluster 0); 1,483 (cluster 1); 1,431 (cluster 2); 1,344 (cluster 3); 1,265 (cluster 4); 957 (cluster 5); 905 (cluster 6); 898 (cluster 7); 662 (cluster 8); 448 (cluster 9); 441 (cluster 10); 265 (cluster 11); and 128 (cluster 12). **b**, Normalized expression of key markers projected on the UMAP. Cell-type clusters are highlighted in red. **c**, Dot plot illustrating the expression of key markers across the 13 cell type clusters. Subcluster 10* indicates a subgroup of cells (*n* = 31) from cluster 10 that were re-annotated as CTb (cytotrophoblast-like cells) (Methods and Extended Data Figs. 12d and 14). The colour intensity indicates average expression. The dot size indicates the percentage of cells in the cluster that express the marker. Com., committed; Post. posterior. **d**, Top, heatmap showing normalized expression of PGC markers (SOX17, PRDM1, NANOS3, NANOG and OCT4). Nine cells were OCT4^+^PRDM1^+^SOX17^+^ and NANOS3^+^. Bottom, OCT4^+^PRDM1^+^SOX17^+^ PGC-like cells (*n* = 27) were identified in epiblast-like clusters, with the majority in cluster 4 (*n* = 19, green). **e**, Left, UMAP of integrated human embryonic reference data^27^ consisting of six human embryonic datasets spanning early zygotes, in vitro cultured human blastocysts^29–31^, 3D in vitro cultured human blastocysts until pre-gastrulation stages^32^, and a CS7, 16–19 d.p.f., human gastrula^33^. The colour of each data point corresponds to the cell annotations from the respective publication. Right, grey data points represent embryonic reference cells, as in the left. Coloured triangles represent the projection positions of the neighbourhood nodes from SEM cells onto the human embryonic reference. SEM-CTb*, neighbourhood nodes representing subcluster 10* cells projected on the reference CTb.

It was previously shown that conventional single-cell transcriptomic analysis (scRNA-seq), which lacks spatial information, is insufficient on its own to indicate whether a stem-cell-derived aggregate has embryo-like structure and compartmental organization, as it does not distinguish between correctly organized mouse SEMs and disorganized aggregates^3,25^. Thus, such an scRNA-seq approach is necessary but not sufficient and should be used to reconfirm a cell-type presence within aggregates after the initial critical microscopy and immunostaining-based tests have been conducted, to unequivocally prove the structural integrity of aggregates as SEMs, as was done above. Thus, we moved next to perform a single-cell transcriptomic analysis by Chromium 10x scRNA-seq on selected SEMs (Supplementary Fig. 15a–c). Uniform manifold approximation and projection (UMAP) analysis identified a total of 13 separate cell clusters (Fig. 6a). Cluster annotation was performed based on the expression or lack of expression of previously defined lineage-specific markers that enabled us to annotate all 13 identified cell clusters (Fig. 6b,c): epiblast-like (in total four clusters: OCT4^+^ and SOX2^+^); YS-like and hypoblast-like (in total three clusters: SOX17^+^, APOA1^+^ and LINC00261^+^ in addition to GATA6^+^, GATA4^+^ and PDGFRA^+^); ExEM-like (in total four clusters: FOXF1^+^, VIM^+^ and BST2^+^, in addition to GATA6^+^ and PDGFRA^+^); amnion-like (one cluster: ISL1^+^, GABRP^+^ and VTCN1^+^); and syncytiotrophoblast-like (one cluster: SDC1^+^, GATA3^+^ and CPM^+^) (Fig. 6a–c).

**Extended Data Fig. 11:**
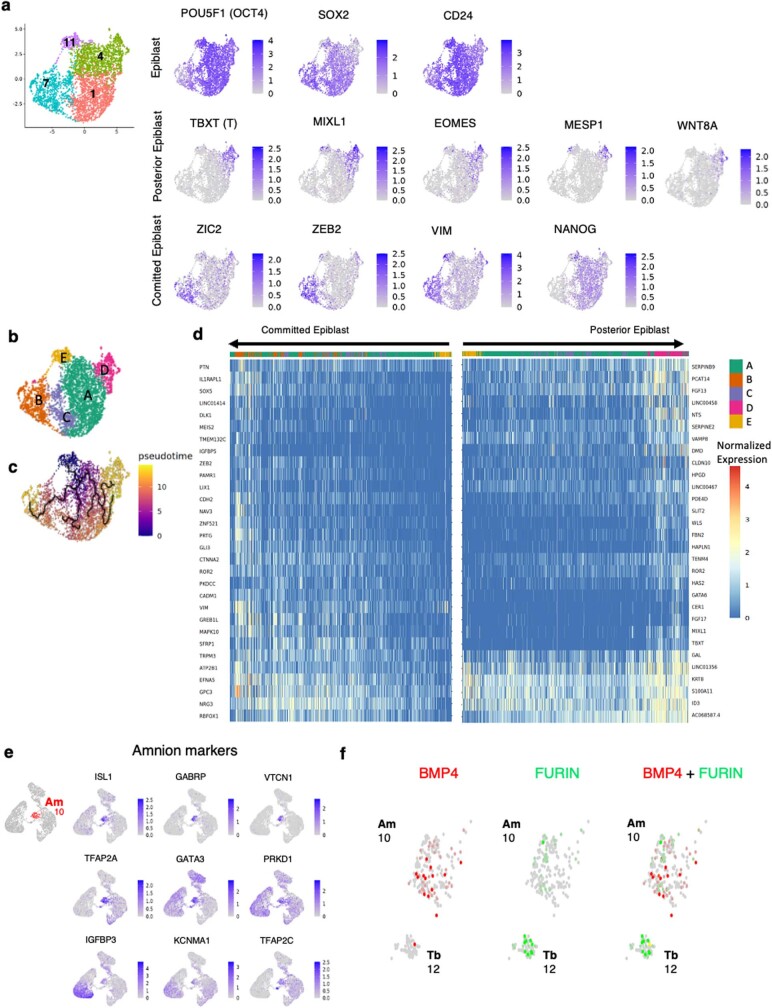
Identification and validation of specific cell sub-types in human SEMs. **a**, UMAP of the four annotated epiblast-like clusters (1, 4, 7 & 11) alongside normalized expression of key marker genes. From the four Epi-like clusters we subclassified two. The first we termed “Posterior epiblast-like cluster” (#4), which was characterized by upregulation of the EMT markers such as TBXT (T/Brachyury), MIXL1, EOMES, MESP1 and WNT8a. The second we termed a “committed epiblast-like” cluster (#7), which was marked by ZIC2, ZEB2, and VIM lineage commitment markers and the absence of NANOG, while maintaining OCT4 and SOX2. **b**, reclustering of the 4 epiblast-like clusters (1, 4, 7, 11) resulted in 5 finer clusters (A-E). **c**, pseudotime analysis over epiblast-like cells starting with epiblast-like (E+A) and progressing through two trajectories towards either committed epiblast-like cells (B) or posterior epiblast-like cells (D). **d**, gene expression profile of top 30 markers of committed epiblast-like sub compartment **(left)** or posterior epiblast-like sub compartment **(right)**. Cells are ordered by cell pseudotime score, showing the gradual increase in expression over pseudotime. **e**, normalized expression of key amnion marker genes projected on human SEM-related UMAP. Amnion-like cluster (#10) is highlighted in red. **f**, expression of BMP4 and FURIN in Amnion (Am)-like cluster 10 and STb-like cluster 12.

From the four annotated epiblast-like clusters (all being OCT4^+^SOX2^+^) (Extended Data Fig. 11a), we subclassified two of them. The first one, which we termed posterior epiblast-like cluster (cluster 4), was marked by the upregulation of T and the co-expression of MIXL1, EOMES, MESP1 and WNT8A (Extended Data Fig. 11a), which are all markers of the epithelial-to-mesenchymal transition process, known to accompany the upregulation of T during peri-gastrulation in mammalian species. The second epiblast-like cluster (cluster 7) we called committed epiblast, as it was marked by the upregulation of ZIC2, ZEB2 and VIM lineage commitment marker expression and the absence of NANOG while maintaining OCT4 and SOX2 pluripotency markers **(**Extended Data Fig. 11a). Pseudotime analysis of epiblast-like cells (Extended Data Fig. 11b–d) showed progression of the transcriptional profile, starting with the unpatterned epiblast-like cells and progressing through two trajectories towards either committed epiblast-like or posterior epiblast-like cells, a result consistent with developmental progression in non-human primates^8^.

**Extended Data Fig. 12:**
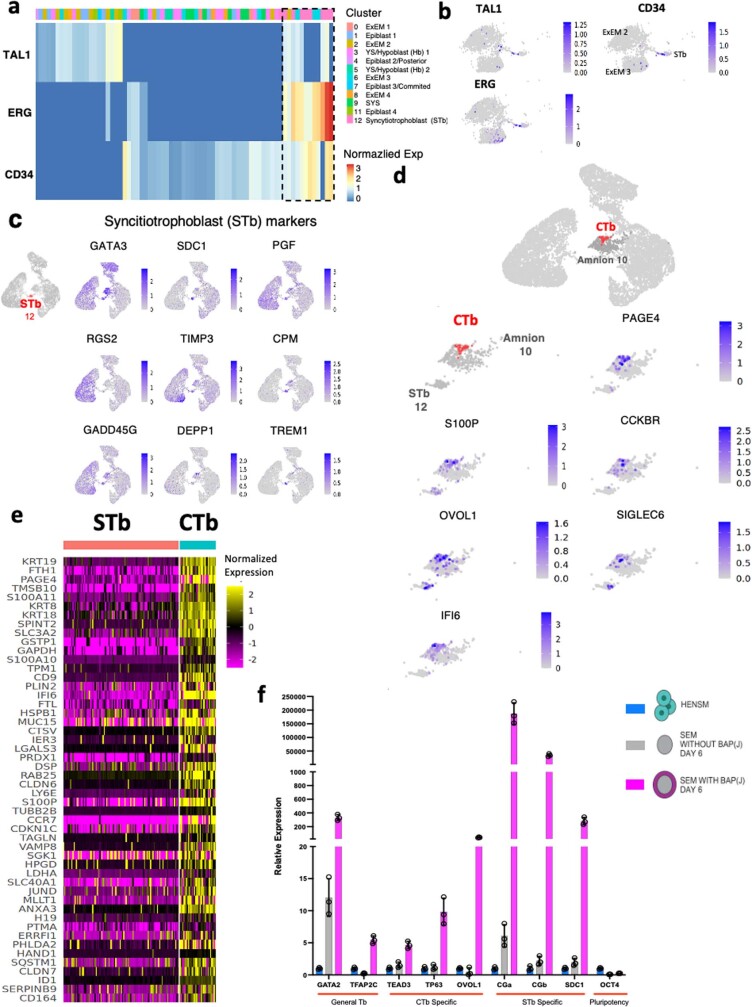
Identification and validation of blood- and trophoblast-like cell sub-types in human SEMs. **a**, normalized expression of selected blood markers (TAL1, ERG, CD34), across cells with positive (>0) expression of CD34 or TAL1. In the dashed area 6 cells which are positive to all 3 markers can be observed. **b**, normalized expression of key blood marker genes (TAL1, ERG, CD34) projected on a subset of SEM UMAP clusters. **c**, normalized expression of key trophoblast marker genes projected on SEM UMAP. Syncytiotrophoblast (STb)-like cluster (#12) is highlighted in red. **d**, normalized expression of key CTb marker genes projected on SEM UMAP clusters 10 (Amnion-like) and 12 (Syncytiotrophoblast-like). Cytotrophoblast (CTb)-like cells are highlighted in red. **e**, expression of top 50 differentially expressed genes (two-sided Wilcoxon test p-value < 0.05, logFC > 1.06 set as threshold), upregulated in Cytotrophoblast (CTb)-like cells compared to Syncytiotrophoblast (STb)-like cells. Annotation of CTb-like cells (n = 31) and STb-like cells (n = 100) was done based on the projection of SEM UMAP on the embryonic reference map (Fig. 6e). **f**, representative RT-qPCR gene expression (normalized by HPRT and RPL3) measured in SEM cells generated with or without BAP(J), and HENSM as control. Cell type markers were measured as following: general Tb markers (GATA2, TFAP2C), CTb-specific markers (TEAD3, TP63, OVOL1)^14^, STb-specific markers (CGa, CGb, SDC1) and pluripotent marker OCT4 (used as a negative control). Bar plot based panel showing the average value of each sample (which represents average value of 3 technical replicates), error bars indicate s.d. A single representative experiment out of N=3 biological replicates performed is shown. Source Data

PGC-like cells (OCT4^+^PRDM1^+^SOX17^+^, a human definitive PGC marker combination^26^) (*n* = 27) could be also identified in SEMs within epiblast clusters, with most of them (*n* = 19) in cluster 4 (Fig. 6d). Rare cells expressing CD34^+^TAL1^+^ERG^+^ could also be detected, which corresponded to early blood progenitors (Extended Data Fig. 12a,b). Although we had technical limitations in maintaining high trophoblast viability and recovery rate following different SEM enzymatic dissociations tested, we still observed a well-separated cluster that expressed lineage markers of syncytiotrophoblast-like cells (cluster 12) (Extended Data Fig. 12c). Although amnion cells can share certain markers with trophoblasts (such as GATA3 and TFAP2A)^16,27^, they formed a separate cluster from syncytiotrophoblasts and expressed amnion markers such as ISL1, GABRP and VTCN1, which are specific to amnion but not to cytotrophoblasts or syncytiotrophoblasts (Extended Data Fig. 11e). We note that BMP4 and furin are expressed in a fraction of human SEM-derived amnion-like cells (Extended Data Fig. 11f). Seurat cluster analysis did not produce a separate cluster containing cytotrophoblast-like cells (probably due to the low capture rate for trophoblasts after SEM enzymatic dissociation) when mapping cytotrophoblast-specific markers such as PAGE4 and S100P (ref. ^14^). However, we were able to identify a subcluster (labelled as 10*) within the amnion-like cluster 10, which was also validated and accurately annotated in an integrated human embryonic reference^27^ that expressed other cytotrophoblast-specific markers such as CCKBR, SIGLEC6, OVOL1 and IFI6 (which are not expressed in amnion or in syncytiotrophoblasts) (Extended Data Fig. 12d,e). PCR analysis with reverse transcription (RT–PCR) on day 6 SEMs with and without BAP(J) cell input validated the detection of both syncytiotrophoblast-specific (TP63, TEAD3 and OVOL1)^14^ and cytotrophoblast-specific (CGa, CGb and SDC1) markers only when BAP(J) primed cells were aggregated together (Extended Data Fig. 12f).

**Extended Data Fig. 13:**
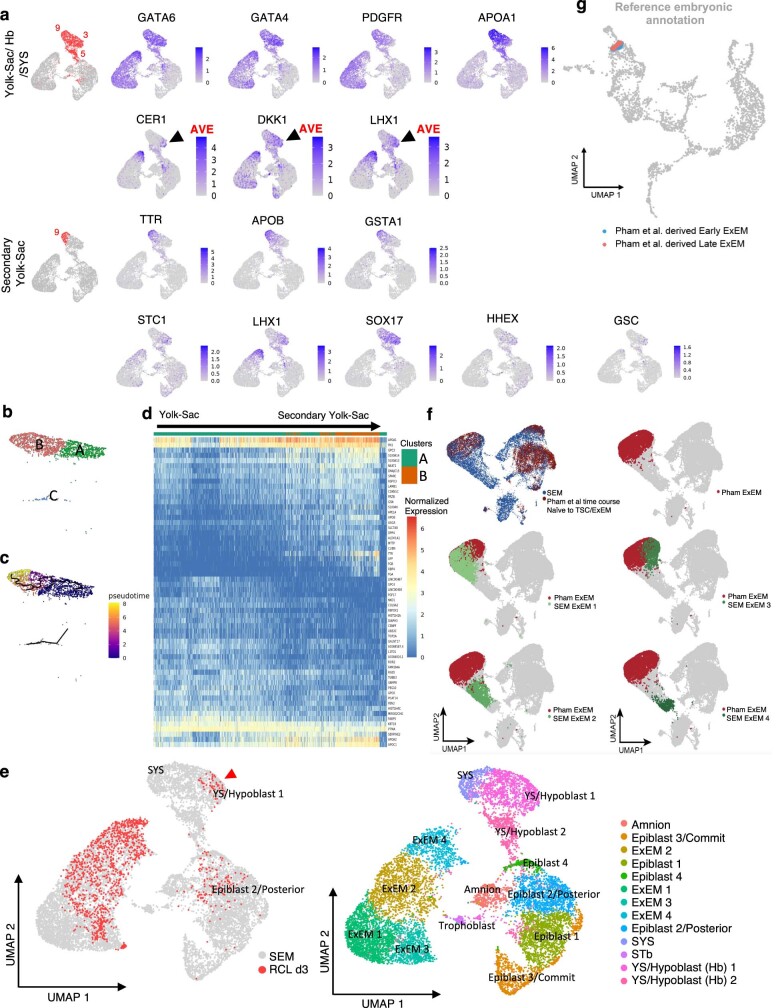
Identification of Primary- and Secondary Yolk-sac-like cells, and Extra-Embryonic Mesoderm-like cells. **a**, normalized expression of key yolk sac (YS) marker genes projected on the SEM UMAP. The co-expression of DKK1 and LHX1 alongside CER1 among SOX17+ YS-like cell population (arrows) in cluster 3 marks AVE-like cells. **b**, reclustering of the two YS-like clusters (3 and 9) resulted in 3 finer clusters (A-C). **c**, pseudotime analysis over YS-like clusters showing progression of the transcriptional profile, reflected by pseudotime score, starting with YS-like structure and ending with SYS-like compartment. **d**, gene expression profile of the top 60 differentially expressed genes (between cluster #3 (YS-like) and cluster #9 (SYS-like), ordered by cell pseudotime score. **e (left)**, projection of day 3 RCL induced cell population (red triangle) on SEM UMAP, showing three cell populations in day 3 RCL fraction: ExEM-like, PrE/Hypoblast-like cells and residual primed ESCs. **e (right)**, the corresponding annotated SEM UMAP. **f**, integration of SEM ExEM-like cells with previously described *in vitro* ExEM^10^ conversion time-course. UMAP of SEM ExEM-like populations (green hues) integrated with the previously published time course dataset of naive to PrE/ExEM conversion^9^ (red hues). **g**, projection of early and late ExEM population derived and sequenced in Pham et al.^9^ on the reference embryo meta-analysis (described in Fig. 6e).

Among the three different YS-like cell clusters that commonly expressed GATA4, GATA6, PDGFRA and APOA1 (clusters 3, 5 and 9), we found that recently identified markers of SYS in marmoset^8^, TTR, APOB and GSTA1, were expressed only in cluster 9, thereby supporting its subclassification as a SYS-like compartment **(**Extended Data Fig. 13a and Fig. 6a,c). STC1 and LHX1 were absent in SYS-like cluster 9, but not in PYS-like clusters 3 and 5, also consistent with findings in marmoset^8^. Lack of uniform HHEX and GSC expression among most cells in all YS-like compartment clusters was consistent with primitive, rather than definitive, endoderm identity for these three YS-like-related clusters^10^ (Extended Data Fig. 13a). The expression of both DKK1 and LHX1, detected using scRNA-seq, alongside CER1 expression among some of the SOX17^+^ cluster 3 YS-like and hypoblast-like cells supports the validity of AV-like cell identity in SEMs (Extended Data Fig. 13a). Pseudotime analysis of YS-like compartment clusters showed a progression of the transcriptional profile, starting with the YS and progressing towards a SYS-like state (Extended Data Fig. 13b–d). Furthermore, PrE-like cells obtained at day 3 following RCL induction mapped to the YS, but not to the SYS, cluster, further indicating that maturation towards SYS-like identity occurred during SEM ex utero culture (Extended Data Fig. 13e). The four identified ExEM-like clusters in SEMs, based on FOXF1 and VIM expression (Fig. 6b), aligned in a single cluster together with previously described reference human ExEM cells^9^ (Extended Data Fig. 13f,g).

We next conducted a comparison analysis of the transcriptional profile of cells in the human SEM dataset to an integrated human embryonic reference^27^ consisting of six human embryonic datasets spanning early zygotes^28^, in vitro cultured human blastocysts^29–31^, 3D in vitro cultured human blastocysts until pre-gastrulation stages^32^, and a CS7, 16–19 d.p.f., human gastrula^33^ (Fig. 6e). UMAP confirmed the annotated identity of the SEM clusters and validated their resemblance to the transcriptome and cell-type composition of early post-implanted human embryos (Fig. 6e and Supplementary Figs. 15d,e and 16). Notably, some human SEM-derived cells projected on primitive streak cells annotated in the integrated human embryonic reference (Fig. 6e), suggestive of a peri-gastrulation stage in some SEMs. Projection of SEM cells onto the human embryonic reference further highlighted that the transcriptional profile of cells differed from human pre-implantation embryos as expected, and all cells corresponded to a post-implanted lineage (Fig. 6e).

**Extended Data Fig. 14:**
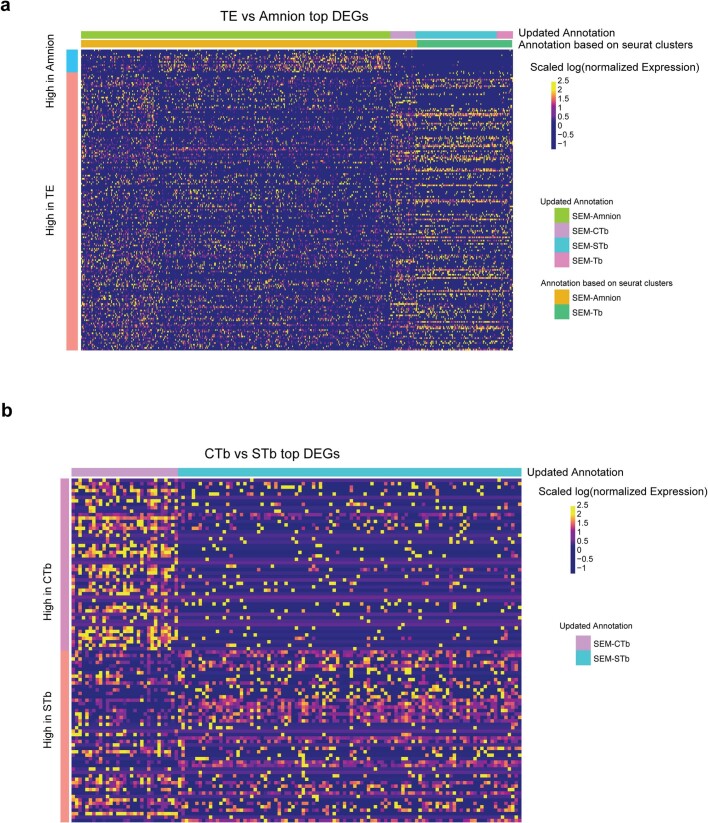
Annotation and characterization of STb- and CTb-like cell populations in human SEMs. **a**, expression of significant differentially expressed genes between Amnion and Tb taken from Zhao et al.^27^, in SEM amnion-like cells and Tb-like cells (performed using the FindMarkers function from R Seurat package and the ‘roc’ test). Seurat annotation and updated annotation from embryonic reference projection were indicated above. **b**, expression of top 50 significant differentially expressed genes between STb and CTb from^32^, in SEM CTb-like and STb-like cells (using the ‘roc’ test of FindMarkers function).

Seurat cluster analysis did not resolve a separate distinct cytotrophoblast-like cell cluster, although detailed marker analysis suggested that a subset of the amnion cluster 10 is cytotrophoblast-like cells, as indicated above (Fig. 6a,c and Extended Data Fig. 12d–f). The integrated human embryonic reference resolved the presence of cytotrophoblast-like cells among the amnion-like cells within the Seurat cluster 10 (called CTb-like subcluster 10*) (Fig. 6a,e and Supplementary Fig. 15d,e). Examining an extended panel of amnion and trophoblast markers^32^ further supported this result, thereby establishing final lineage annotation of amnion, cytotrophoblast and syncytiotrophoblast cells (Extended Data Fig. 14 and Supplementary Tables 1 and 2). This analysis validated the detection of post-implantation epiblast-like, YS-like, ExEM-like, syncytiotrophoblast-like and cytotrophoblast-like cells, as well as the emergence of other transcriptomic states, such as amnion-like, hypoblast-like and YS-like and posterior epiblast-like cells and compartments (Fig. 6e, Supplementary Fig. 15d,e and Extended Data Fig. 14). Overall, despite the known limitations of cross-dataset, cross-platform comparisons, the single-cell transcriptomic analysis presented above supports the conclusion that human SEMs recapitulate lineage differentiation of the early human post-implantation embryo.

## Discussion

Studying early human post-implantation development is crucial for understanding human embryogenesis, developmental birth defects and early pregnancy loss. Moreover, optimizing protocols for in vitro directed differentiation of human PS cells into mature cell types would greatly benefit from understanding the key mechanical, transcriptional and signalling pathways active during early embryogenesis to improve PS cell differentiation quality and efficiency. Such research endeavours would require large numbers of donated human embryo-derived materials from post-implantation stages; however, justified ethical barriers and the scarcity of such samples make conducting human embryo-based research ethically and technically impossible. Given the capacity to generate both embryonic cells and extra-embryonic primed cells from human naive PS cells that can assemble into structured and dynamic human stem-cell-derived models capable of mimicking the key developmental milestones occurring during the early post-implantation stages is becoming a necessary element to establish advanced-stage fully integrated and complete embryo models^1^.

Here we built on our recently described approach in mice^3^ to generate self-organizing complete post-implantation SEMs solely from naive ES cells, without the need to derive and utilize extra-embryonic cell lines from embryo tissue samples^3^. The latter technical finding is of great importance, as it suggested that only establishing naive PS cells may be sufficient to enable the generation of advanced embryo-like structures ex utero from other mammals, including humans^3^. Remarkably, the protocol developed here to generate complete human SEMs relies on starting solely from naive PS cells and does not even require the genetic modification or overexpression of exogenous lineage factors for priming the naive ES cells towards the three different extra-embryonic lineages prevalent at these developmental stages, contrary to what is currently still required in mouse SEM derivation protocols^3^. The latter further underscores the importance of devising human naive and naive-like pluripotency conditions in recent years^4,7^ as a critical determinant for enabling the formation of complete human SEMs, as these cells retain the self-organization ability to generate both embryonic compartments and all extra-embryonic compartments^4^, including the ExEM, and suggest that human naive PSCs are de facto totipotent. The human SEMs generated ex utero here mimicked the 3D architecture and key developmental landmarks of in utero-developed natural human embryos from 7–8 d.p.f. to 13–14 d.p.f. (CS5a to CS6a). We observed proper spatial allocation of cell lineages into defined and correctly positioned embryonic and extra-embryonic compartments in the complete absence of fertilization or interaction with maternal tissues and without the need to provide external directed signalling pathway induction during the self-organization of the aggregated cells. Moreover, we emphasize that our goal is to make an embryo model with recognizable recapitulation of the key milestones and the crucial embryonic structures, which may be helpful for research purposes even if it is not fully identical to the natural human embryo. At the structural level, our human SEM highly resembles, but not identical, to the in utero situation.

While this initially preprinted manuscript^2^ was under revision, two papers reported aggregates derived from human stem cells expanded in our previously published naive-like RSeT media^7^, with claims of mimicking stages of human post-implantation development^34,35^. However, their generated aggregates did not contain the most elementary and defining hallmarks of integrated embryo models and therefore are more akin to EBs that contain certain extra-embryonic cells but lack organization patterns that resemble those characteristic of any stage of human embryo development in utero. Specifically, such aggregates showed an absence of essential key cell lineages of the developing embryo (for example, trophoblast lineage, visceral and parietal endoderm), and lacked key hallmarks of structural compartments with correct morphological organization (for example, embryonic disc-like, hypoblast-like, bilaminar-disc-like, YS-like, ChC-like and surrounding trophoblast-like compartment). Moreover, they lacked dynamism relating to the ability to progress structurally to the next stages in development following initial aggregate formation^3^. Thus, following their inability to meet any of the elementary criteria and hallmarks laid out in the introduction, these aggregates should not be categorized as models for embryos and cannot be developmentally assigned a Carnegie stage, as such developmental day staging is based entirely on structural organization and embryo-like morphological criteria.

The current low efficiency and developmental-stage variability observed during the formation of our human SEMs are limiting factors that need to be overcome to facilitate the use of such platforms for certain experimental set-ups. Nevertheless, the emergence of well-defined complete structures suggests that this will probably be possible in the future (Supplementary Information). It will be of interest to further explore whether omission of RCL-induced cells and relying instead on the ability of HENSM naive ES cells to spontaneously give rise to PrE and ExEM lineages in N2B27 basal conditions can be altered to produce equivalent or enhanced SEM outcome to the co-aggregation regiments utilized here. Given that human embryo implantation invades the endometrium entirely^15^, it will also be important to experiment whether further and better trophoblast-like compartment development and maturation (for both cytotrophoblast-like and syncytiotrophoblast-like cells) would occur if human SEMs would be embedded within different extracellular microenvironments. Finally, testing whether human SEMs described here can develop further towards completing gastrulation and advancing through organogenesis, as recently achieved with mouse SEMs exclusively made from naive ES cells^3^, may be of experimental importance and will offer insights into previously inaccessible windows of early human development and might contain authentic differentiated somatic cell types that could be useful for cell-transplantation therapy and tissue regeneration.

## Methods

### Ethics

All experiments reported herein involving human ES cells and iPS cells were conducted after obtaining approval by the Weizmann Institutional Review Board (IRB) (approval number 1868-2) to generate human embryo(id) models (with and without transgene expression to form extra-embryonic cells) from human ES cell and iPS cell lines^36^. Consent forms for all the previously derived human ES cell lines used in this study (WIBR1, WIBR2, WIBR3 and RUES2 human ES cells) allow their use in human embryo model development and research conducted here. The newly derived human iPS cell lines used herein (JH22 and JH33 iPS cells) were specifically and explicitly consented for use in human embryo model development according to Weizmann Institutional IRB approval numbers 1871-2 and 1868-2. All the experiments reported herein follow the latest ISSCR guidelines released in 2021 (ref. ^37^). This study does not involve the derivation of new human ES cell lines, does not use any newly obtained samples from fetal abortions and does not use any newly donated human blastocysts. Furthermore, this study does not involve in utero transfer of any human SEMs into any other species, consistent with ISSCR guidelines and Israeli legislation. Finally, all the human SEMs described herein do not correspond to developmental stages beyond 14 d.p.f. Not all the features of a 14-day human embryo were present by morphology and immunostaining at the experimental end point.

### Data reporting

No statistical methods were used to predetermine sample sizes. Samples were randomly allocated when placed in the different growth conditions. Other experiments were not randomized. The investigators were not blinded to allocation during experiments and outcome assessment as there was no relevant scientific reason to do so. Throughout the manuscript, all data points and samples represent biological replicates (*N* indicated in figure legends or in graphs; *n* number of samples per biological replicate is indicated where relevant), except for RT–PCR, for which per individual representative experimental graph, each sample was run as a technical triplicate per each gene. The number of biological replicates of each RT–PCR panel from which the representative experiment was taken and shown is indicated per panel.

### Statistics and reproducibility

The following figure panels showing microscopy images are representative of independent biological replicates as follows (*N*) as detailed: Fig. 1: e, *N* = 3; g, *N* = 11; Fig. 2: d, *N* = 11; Fig. 3: a,b, *N* = 3; c, *N* = 11; e–k, *N* = 2; m, *N* = 3 ; Fig. 4: a, *N* = 12; d, *N* = 11; e–i, *N* = 5; k, *N* = 3; Fig. 5: a,f, *N* = 11; c,d,g, *N* = 5; Extended Data Fig. 2: a–c, *N* = 4; Extended Data Fig. 3: c, *N* = 3; d, *N* = 4; Extended Data Fig. 4: b, *N* = 4; Extended Data Fig. 5: b, control, *N* = 11; b (no HENSM),e, *N* = 3; b, no BAP(J), *N* = 4; b, no RCL, *N* = 3; d, *N* = 3; e, *N* = 3; Extended Data Fig. 6: a, *N* = 2; b,c, *N* = 11; Extended Data Fig. 7: b, *N* = 5; Extended Data Fig. 8: a,c–e,g, *N* = 3; f, *N* = 3, h, *N* = 3; Extended Data Fig. 9: a,b,g,h, *N* = 3; c, *N* = 3; d,e, *N* = 5; Extended Data Fig. 10: a, *N* = 11; b,c, *N* = 7; e,f, *N* = 3; i, *N* = 2; Supplementary Fig. 1: b, *N* = 5; Supplementary Fig. 2: a,b, *N* = 4; Supplementary Fig. 4: b, *N* = 6; c–f, *N* = 3; Supplementary Fig. 5: a, *N* = 3; c, *N* = 3; Supplementary Fig. 6: a,c,d, *N* = 3; Supplementary Fig. 7: b, *N* = 3; Supplementary Fig. 8: b,c, *N* = 5; Supplementary Fig. 9: b,c, *N* = 3; Supplementary Fig. 10: a, *N* = 3; b, *N* = 4; d, *N* = 2; e, *N* = 3; Supplementary Fig. 11: b, *N* = 11; c, *N* = 3; Supplementary Fig. 12: b,d, *N* = 3; c,e,f, *N* = 11; g,h,j, *N* = 4; Supplementary Fig. 13: b, *N* = 3; c, *N* = 3; and Supplementary Fig. 14: *N* = 3.

### PS cell lines

The following already established human ES cell lines were used: WIBR3 (WT female) and WIBR1 (WT male) human ES lines^36^. The WIBR2 female human ES cell line so far has failed to give us validated complete day 8 SEMs. The RUES2 human ES cell line carrying fluorescent reporters for endoderm, mesoderm and ectoderm differentiation (a gift from A. Brivanlou) was also used to validate endogenous marker gene expression in SEMs through the reporters. Newly derived JH22 and JH33 iPS cell lines (taken from a healthy adult male donor from the Middle East and obtained following donor informed consent to make genetically unmodified iPS cell lines from donated peripheral blood (as approved by the Weizmann IRB, approval number 1871-2)), were used for lineage priming efficiency experiments where indicated. The Lis49 human ES cell line was used for naive versus primed scRNA-seq comparison alongside the WIBR3 human ES cell line. The previously established mouse Tet-ON iGATA4 KH2-WT ES cell line was used for comparing the induction efficiency of PDGFRA^+^ cells^3^. The Tet-ON iGATA4 KH2-WT ES cell line, the Tet-ON iGATA4 KH2-iCdx2 ES cell line and the WT BVSC mouse ES line were used for mouse SEM experiments. All cell lines were routinely checked for mycoplasma contamination every month (Lonza–MycoAlert), and all samples analysed in this study were not contaminated.

### Human HENSM naive and RSeT naive-like PS cell in vitro culture conditions

Golden stocks of human PS cells were cultured on a feeder layer of irradiated MEFs (from embryonic day 13.5 ICR-DR4 mouse embryos) and maintained in conventional human FGF/KSR primed conditions and frozen for future experimentation. The FGF/KSR conditions on MEF substrate were as follows: 400 ml of DMEM/F12 (Invitrogen, 10829) supplemented with 20% knockout serum replacement (KSR; Invitrogen, 10828-028), 1 mM GlutaMAX (Gibco, 35050061), 1% nonessential amino acids (NEAA; BI, 01-340-1B), 1% sodium pyruvate (Biological Industries, 03-042-1B), 1% penicillin–streptomycin (Biological Industries, 03-031-1B) and 8 ng ml^–1^ bFGF (Peprotech, 100-18B-1MG).

The primed human PS cell lines were routinely expanded as naive-like cells in RSeT medium (Stem Cell Technologies, 17148311) (RSeT medium (2-component) is a commercialized version of NHSM medium that has been previously described^7^), and was assembled according to the manufacturer’s instructions, including dissolving 250 μl Matrigel in 500 ml medium as instructed; https://www.stemcell.com/products/rset-medium-2-component.html), and expanded on Matrigel or Cultrex-coated plates and passaged with TrypLE every 3–5 days. These were kept for up to 40 passages in RSeT medium (with and without freezing–thawing in this state) and used to quickly and homogenously convert from naive-like cells into naive cells in HENSM conditions (a detailed step-by-step protocol for key experiments in this study is published on Protocol Exchange^38^.

To reprogramme the latter naive-like ‘formative’ RSeT PS cells into a naive HENSM cells, human ES cells were transferred and expanded as previously described^4^ in serum-free HENSM on plates coated with 1% Matrigel (Corning, 356231) (a detailed HENSM protocol with more explanations and troubleshooting is also available at the Hanna Laboratory website protocol section: https://www.weizmann.ac.il/molgen/hanna/) (or less preferably for SEM protocol on MEF/gelatin-coated plates). The HENSM version used herein consists of the following: 470 ml of 1:1 mix of Neurobasal (Invitrogen, 21103-049) and DMEM/F12 (Invitrogen, 21331), 5 ml penicillin–streptomycin (Biological Industries, 03-033-1B), 5 ml GlutaMAX (Invitrogen, 35050061), 5 ml NEAA (Biological Industries, 01-340-1B), 5 ml sodium pyruvate (Biological Industries, 03-042-1B), 10 ml B27 supplement (Gibco, 17504-044 (lots used: 2450392, 2415388 and 2450384) or prepared in-house or by Lifegene NCS21 -(lot used: D5482)), 50 μg ml^–1^ l-ascorbic acid 2-phosphate (vitamin C) (Sigma, A8960), 1 ml Geltrex (Invitrogen, A1413202/A1413302) (0.2% final concentration), 5 ml N2 supplement (Gibco, 17502048 or prepared in-house), 10 ng ml^–1^ LIF (Peprotech, 300-05 or prepared in-house), 2 μM of the WNT and TNK inhibitor XAV939 (Sigma X3004), 2 μM of the PKC inhibitor Gö6983 (Axon 2466), 1–1.2 μM of the MEK1/2 inhibitor PD0325901 (Axon 1408), 1–1.2 μM of the SRC inhibitor CGP77675 (Axon 2097 (batch 9 or 10) or Sigma Aldrich, SML0314 (batch 10)), 5 ng ml^–1^ activin A (Peprotech, 120-14E), 1–1.2 μM of the ROCK inhibitor Y27632 (Axon Medchem 1683), and 0.8 μM BIRB796 P38i (Axon, Medchem 1358). At least 2 passages in HENSM conditions were applied before cells were used for experiments. Human naive HENSM PS cell lines were used for up to 10 passages after transfer into HENSM conditions. Naive HENSM cells were routinely assessed by FACS to confirm high expression of the naive pluripotency cell surface marker CD77, which is totally absent on human primed PSCs. For maintenance of ES cells/iPS cells in naive HENSM conditions, cells were passaged every 3–5 days using TryplE (Gibco, 12604054). Different batches of B27 can produce some notable differences in the level of expression of naive pluripotency markers and cell growth rate of the same PSC line, which may require small adaptation of the SEM protocol at the extra-embryonic cell-priming step to maintain cell priming and SEM derivation efficiency and quality (see associated Protocol Exchange document for relevant details and troubleshooting^38^). Because large numbers of cells were needed to obtain a proper induction yield per experimental batch, we used HENSM that includes low-dose 5 ng ml^–1^ activin A, which is a booster for human naive cell proliferation rate (as previously indicated^4^). We could not reach very high numbers of cells in HENSM conditions without activin A to meaningfully run experiments and reach equivalent conclusions. Naive HENSM, naive-like (RSeT) and primed human ES cells were expanded and primed towards different lineages in a 5% CO_2_ incubator at 5% O_2_ at 37 °C. If HENSM cells were not homogenous and differentiated cells were apparent, we checked that HENSM was assembelled according to the protocol and no components were mistakenly omitted. We also considered using a different B27 batch or B27 from a different vendor, and ensured that the medium was not more than 10 days old. Some PS cell lines may require small modifications in the HENSM protocol (for example, a slight increase in activin A dose used or a decrease in MEK1/2 inhibitor used). Refer to the Hanna Laboratory HENSM protocol detailed specifics posted on the J.H.H. Laboratory website (https://www.weizmann.ac.il/molgen/hanna/) for further standardization and explanation, if needed.

### Generation of iGATA4, iGATA6, iCDX2 and iGATA3 human ES cell clones

To generate Tet-ON inducible lines, we used a PiggyBac plasmid expressing a cDNA insert and transposase vector of choice under the control of a doxycycline-inducible promoter (a gift from V. Busskamp, Addgene, plasmid 104454). The donor vector carries M2RtTa and a site for cDNA insert of the transcription factor of interest. We used this vector to generate four different doxycycline-inducible lines in WIBR3 WT human female ES cells: human iCDX2 or human iGATA3 (to promote human ES cell differentiation towards trophectoderm) and human iGATA4 or human iGATA6 (to promote human PrE/ExEM priming from human ES cells). Puromycin selection was applied for approximately 6–8 days. Resistant clones were picked and cultured for downstream characterization. Insertion was validated by immunostaining after doxycycline (2 µg ml^–1^) induction of the gene of interest. Transgene expression was verified to be specifically detected only after doxycycline addition in the corresponding lines. Detailed generation, characterization and validation of these lines can be found in Extended Data Figs. 1, 3 and 7. Generation of a fluorescently labelled WIBR3 iCDX2 line was made after transduction with lentivirus constitutively expressing tdTomato protein. For lentivirus generation, HEK293T cells were used as the most conventional and commonly used line for lentiviral packaging and generation. HEK293T cells were plated on 10-cm dishes filled with 10 ml DMEM 10% FBS and penicillin–streptomycin, at a density of 5.5 million cells per plate. On the next day, cells were transfected with second-generation lentiviral vectors (Addgene, 8454 and 8455) using X-tremeGENE 9 transfection reagent along with 16 μg of the target plasmid for the transduced fluorescent protein (tdTomato). The supernatant containing the virus was collected 48 h following transfection, filtered using 0.45 μm filter and concentrated by ultracentrifugation. Human ES cells were plated in mTESR medium on Matrigel-coated 6-well plates at low density. The next day, they were transduced with lentivirus in the presence of protamine sulfate (10 µg ml^–1^) for 6 h, after which the medium was exchanged. After 2 days, the infected human ES cells were expanded for 1 passage and the positive population was sorted using FACS and further expanded for experimentation.

### Derivation of a stable human TS cell line from human ES cells

TS cell lines were produced from naive (HENSM) and primed conditions according to ref. ^12^ and ref. ^13^, respectively. In brief, human naive WIBR3 ES cells were expanded at least 3 passages in naive or primed conditions and then transferred into TS cell medium on 1% Matrigel (Corning, 356231)-coated plates. After 3 passages, stable TS cell lines could be established and could be passaged up to 70 times or more. Cells were expanded in a 5% CO_2_ incubator at 5% O_2_. For maintenance, human TS cells were passaged with TrypLE (Gibco, 12604054) when they reached 70–80% confluency. Immunostaining was performed to confirm human TS cell identity: human TS cells were negative for CDX2 and positive for CK7, GATA3 and TFAP2C, consistent with previous reports. Only confirmed lines were used for SEM experiments. Human TS cell medium used herein has been previously described^13^, but with the following slight modifications: 470 ml DMEM/F12 (Invitrogen, 21331), 5 ml commercial N2 supplement (Invitrogen, 17502048), 10 ml B27 supplement (Invitrogen, 17504-044 or made in-house), 5 ml sodium pyruvate (Biological Industries, 03-042-1B), 5 ml penicillin–streptomycin (Biological Industries, 03-033-1B), 5 ml GlutaMAX (Invitrogen, 35050061), 5 ml NEAA (Biological Industries, 01-340-1B), 50 μg ml^–1^ l-ascorbic acid 2-phosphate (Sigma A8960), 50 ng ml^–1^ human EGF (Peprotech, AF-100-15), 0.75–1 µM of the TGFR inhibitor A83-01 (Axon 1421), 2 µM of the GSK3 inhibitor CHIR99021 (Axon Medchem, 1386) and 5 µM of the ROCK inhibitor Y27632 (Axon, 1683).

### Priming of human naive HENSM ES cells towards TE-like cells for SEM generation

Human TE-like cells were obtained from human naive ES cells expanded in human HENSM conditions for at least 2 passages. At 24 h before the BAP(J) induction–priming initiation, HENSM naive PS cells were seeded in HENSM onto 1% Matrigel (Corning, 356231)-coated plates supplemented with 10 µM of the ROCK inhibitor Y27632 (Axon 1683). The next day after seeding, HENSM was removed and the 72-h BAP(J) protocol was started. The 3-day BAP(J) treatment for human TE-like cell priming–induction has been previously described and is adapted from a previously described method^14^ as follows: BAP(J) medium was used for 72 h in total. This medium consisted of 2 µM of the TGFR inhibitor A83-01 and 2 µM of the MEK1/2 inhibitor PD0325901 base, which was complemented with 10 ng ml^–1^ human recombinant BMP4 (Peprotech) only for the first 24 h, and then BMP4 was substituted with for 1 µM of the JAK inhibitor I (Calbiochem, 420099) on days 2 and 3. The base medium consisted of the following: 470 ml of 1:1 mix of neurobasal (Invitrogen, 21103-049) and DMEM/F12 (Invitrogen, 21331), 5 ml penicillin–streptomycin (Biological Industries, 03-033-1B), 5 ml GlutaMAX (Invitrogen, 35050061), 5 ml NEAA (Biological Industries, 01-340-1B), 5 ml sodium pyruvate (Biological Industries, 03-042-1B), 10 ml B27 supplement (Gibco, 17504-044), 5 ml N2 supplement (Invitrogen, 17502048, or prepared in-house), 2 µM of the TGFR inhibitor A83-01 (Axon Medchem A83-01) and 2 µM of the MEK and ERK inhibitor PD0325901 (Axon Medchem, 1408). The entire process was incubated in a 37 °C incubator with 5% O_2_ and 5% CO_2_. The end of the 72-h BAP(J) induction–priming regimen became day 0 of the SEM co-aggregation protocol, which is the day in which BAP(J)-treated cells were collected and aggregated. It is important to note that cell confluency during TE-like cell induction is essential for high quality and highly efficient reproducible results and needs to be calculated for each cell line and condition tested. In our case for the WIBR3 human ES cell line growth on Matrigel, 1 × 10^6^ HENSM PS cells per 10-cm Matrigel-coated plate showed optimal results. For the WIBR1 human ES cell line grown in HENSM on Matrigel-coated plates, seeding 2 × 10^6^ HENSM cells on a 10-cm Matrigel-coated dish and then initiating the BAP(J) regimen gave optimal results as checked by FACS on day 3 of the BAP(J) regimen. We recommend conducting a cell confluency plating curve for BAP(J) induction to be checked on day 3 after induction initiation using ENPEP/TACSTD2 expression by FACS, which can give an idea on adequate HENSM PS cell numbers to be seeded for different cell line calibration of the optimal TE-like cell priming expected outcome, which then leads to successful SEM generation after moving to the aggregation stage of the protocol.

To validate the identity of the starting BAP(J) cells derived after 3 days from HENSM ES cells, we applied scRNA-seq and integrated the data with a reference naive TE and naive cytotrophoblast differentiation protocol involving BAP(J) during the first 3 days from t2iLGö cells^14^. BAP(J) cells induced herein for 3 days aligned predominantly to previously described day 2 and day 3 naive TE cells but not day 10 naive cytotrophoblasts (Extended Data Fig. 3g). Thus, BAP(J) treatment induces naive TE-like cells from WT human ES cells expanded in our devised HENSM conditions. Please note that the cells induced using the BAP(J) regimen do not maintain their TE-like identity if maintained in BAP(J) for more than 3–4 days or if moved to N2B27 medium after day 3 (which was used in the first 3 days of SEM formation protocol). This indicates that the switch from BAP(J) to N2B27 is not the reason for the current reduced efficiency of SEM formation.

### PrE-like and ExEM-like cell priming from human naive HENSM PS cells

PrE-like and ExEM-like cells were primed and induced from human naive ES cells expanded in HENSM conditions for at least 3 passages as described above. For priming into PrE-like and ExEM-like cells, HENSM cells were plated onto gelatin-MEF-coated plates in HENSM medium with 10 µM of the ROCK inhibitor Y27632 the day before the 72 h of RCL induction initiation. On the next day after cell seeding, HENSM medium was changed to RCL for 72 h, and the RCL medium was exchanged every 24 h. RCL is composed of the following: 480 ml RPMI medium (Gibco, 21875-03), 10 ml B27 minus insulin supplement (Invitrogen, A18956-01), 1 mM GlutaMAX (Invitrogen, 35050061), 1% penicillin–streptomycin (Invitrogen), 3 µM CHIR99021 (Axon Medchem, 1386) and 10 ng ml^–1^ LIF (Peprotech, 300-05). RCL medium contains the same composition as RACL but without adding recombinant activin A. WT human naive ES cells or iGATA4 and iGATA6 cell lines were used for induction in the presence or absence of doxycycline as indicated. The entire process was incubated in a 37 °C incubator with 5% O_2_ and 5% CO_2_. The end of 72 h RCL induction–priming regimen became day 0 of the SEM aggregation protocol, which is the day in which RCL-treated cells are collected and aggregated together. Please note in Fig. 1d, 6 days of RCL induction or 3 days of RCL and 3 days of basal N2B27 induction produced similar outcomes of PDGFRA^+^ fraction. Therefore, in this case, the switch of medium is not the reason for the reduced efficiency in human SEM formation described here.

Note that cell confluency during RCL induction is essential for high efficiency and reproducible induction results and need to be calculated for each HENSM naive PS cell line tested. In our case for the WIBR3 cell line grown in HENSM on Matrigel in preparation for RCL induction, plating 8 × 10^5^ HENSM naive ES cells per one 10-cm MEF-coated Petri dish showed the best results regarding RCL induction efficiency outcome at day 6. By contrast, in the case of the WIBR1 cell line, plating 2 × 10^6^ HENSM PS cells per one 10-cm MEF-coated Petri dish showed optimal RCL induction results on day 6. For each HENSM naive line calibration, we recommend conducting cell number plating curves, followed by using PDGFRA expression by FACS on day 6 after RCL induction initiation, which can give experimentalists a good estimate of the optimal HENSM PS cell numbers needed to be plated for each line to obtain optimal induction efficiency of PrE-like and ExEM-like cells that then leads to successful SEM generation after moving to the co-aggregation stage of the protocol.

### Generation of human SEMs

To generate SEMs from human naive pluripotent cells (WIBR1, WIBR2, WIBR3 and RUES2 human ES cell lines), three starting cell mixtures were aggregated together using AggreWell 400 24-well plates (Stem Cell Technologies, 34415): (1) Naive ES cell and naive PS cell WT cells cultured in HENSM medium in a 5% CO_2_ incubator at 5% O_2_ at 37 °C. (2) For the PrE-like and ExEM-like compartments, naive WT cells were plated on irradiated MEF–gelatin-coated plates in HENSM supplemented with 10 µM of a ROCK inhibitor (Axon Medchem, 1683). The next day, cells were washed with PBS twice (without collection), and HENSM was replaced with RCL medium. RCL was kept for 72 h, with 24-h medium exchanges in a CO_2_ incubator at 5% O_2_ at 37 °C. (3) For the TE-like lineage, naive WT cells were plated on feeder-free conditions (Matrigel) in HENSM supplemented with 10 µM of a ROCK inhibitor. The next day, cells were washed with PBS twice (without collection), and HENSM was replaced with BAP medium for 24 h, followed by replacement with AP(J) for another 48 h in a 5% CO_2_ incubator at 5% O_2_ at 37 °C (termed the BAP(J) protocol).

Co-aggregation was defined as time point 0 of the protocol. At 12–24 h before aggregation, all donor cells were supplemented with 10 µM of a ROCK inhibitor (Axon Medchem, 1683). At the day of aggregation (day 0), AggreWell 400 24-well plate preparation was done according to the manufacturer’s instructions. In brief, 500 µl of anti-adherence rinsing solution (Stem Cell Technologies, 07010) was added to each well, the plate was centrifuged at 2,000*g* for 5 min and incubated 30 min at room temperature. Subsequently, rinsing solution was removed and the plate was washed with PBS. Each well was filled with 500 µl of aggregation medium and kept at 37 °C for medium equilibration. Aggregation medium (BSA-supplemented N2B27 medium) consisted in 500 ml of a 1:1 mix of neurobasal (Invitrogen, 21103-049) and DMEM/F12 (Invitrogen 21331), 5 ml penicillin–streptomycin (Biological Industries, 03-033-1B), 5 ml GlutaMAX (Invitrogen 35050061), 5 ml NEAA (Biological Industries, 01-340-1B), 5 ml sodium pyruvate (Biological Industries, 03-042-1B), 10 ml B27 supplement (Invitrogen, 17504-044), 5 ml N2 supplement (Invitrogen 17502048), 1 ml β-mercaptoethanol 50 mM (Gibco, 31350-010) and 2.25 ml of BSA solution 35% (Sigma A7979).

The three cell populations were collected using TrypLE (Thermo Fisher, 12604054) (3 min for the HENSM and RCL-induced cell populations, and 5 min for the BAP(J) primed cells) at 37 °C. Afterwards TrypLE was removed by vacuum and the cells were incubated for 2 min at room temperature and cells were subsequently collected with PBS. Cells were centrifuged at 1300 r.p.m. for 3–5 min and resuspended in aggregation medium. Next, RCL-induced cells were plated on gelatinized tissue culture plates on MEF medium consisting of 500 ml DMEM (Gibco, 41965-039) 20% FBS (Sigma, F7524-500ml), 5 ml penicillin-streptomycin (Biological, Industries 03-033-1B), 5 ml GlutaMAX (Invitrogen, 35050061), 5 ml NEAA (Biological Industries, 01-340-1B), 5 ml sodium pyruvate (Biological Industries, 03-042-1B), for MEF depletion for 30 min at 37 °C. At the end of MEF depletion, the supernatant was collected and passed through a 70 µm cell strainer, and all three cell types were centrifuged separately and resuspended and passed through a 70 µm cell strainer in N2B27 medium. The three cell fractions were counted and combined as follows in an AggreWell 400 plate (in each well there are 1,200 microwells): total cell number per single individual microwell per aggregates is 120 cells, total number of cells per a single well of a 24-well plate is 144,000 cells (based on the calculation of 120 cells × 1,200 microwells). Ratio of 1:1:3 (HENSM: RCL: BAP(J)) or (Epi-like: PrE/ExEM-like: TE-like) = 28,800 Epi (HENSM) cells, 28,800 Pre/ExEM-like (RCL) cells, and 86,400 TE-like (BAP(J)) cells per each well of a 24-well AggreWell 400 plate. To start an aggregation, HENSM ES cells/iPS cells (PS cells) on day 3–4 after passaging, RCL day 3 and BAP(j) day 3 cells are needed. BAP(J) cells are required in bigger amounts because aggregation ratios are 1:1:3 (ES:RCL:BAP(J)), so usually 2–3 × 10 cm plates of BAP(J) primed cells are prepared per 1 × 10 cm plate of HENSM naive PS cells and 1 × 10 cm plate of RCL primed fraction. A detailed step-by-step protocol for making human SEMs is published on Protocol Exchange^38^ and includes a supplementary Excel sheet macro-template for cell number and ratio calculation. Complete cell mixtures after cell counting were prepared as 2× concentration (288,000 cells per ml with 20 µM of ROCK inhibitor). A volume of 500 µl of cell mix suspension was gently added dropwise to each well of the AggreWell plate (final yield per each well = 1 ml final volume with 10 µM final ROCK inhibitor concentration and 144,000 cells). The plate was centrifuged at 100*g* for 3 min and incubated at 37 °C in hypoxic incubator conditions (5% O_2_ and 5% CO_2_).

The next day (day 1), 1 ml of aggregation medium per well was pre-warmed for 30–60 min at 37 °C in a water bath. The AggreWell plate was removed from the incubator and observed under a microscope to ensure that cells had started to form aggregates inside the microwells. Approximately 800–900 µl of medium was gently removed from each well and 1 ml of pre-warmed aggregation medium was gently added to each well. The plate was returned to the hypoxic incubator. The same volume of medium was replaced at day 2, and plates returned to 37 °C hypoxia 5% O_2_ 5% CO_2_ incubator.

At aggregation day 3, 6-well cell-suspension non-adherent tissue culture plates (Greiner, 657185) were filled with 3 ml of hEUCM2 (20% FBS) per well and were placed for 30 min on an orbital shaker rotating at 60 r.p.m. (Thermo Scientific 88881102 + 88881123) located inside a 5% CO_2_ incubator in 20% O_2_ for pre-equilibration.  The AggreWell plate containing the aggregates was taken out, and most (nearly all) of the aggregation medium was removed without disturbing the aggregates, and 1 ml of hEUCM 20% taken from the 6-well plate was added (with the goal of transferring aggregates from 2 wells of a 24-well plate to 1 well of a 6-well plate), and 2 ml of the 3 ml hEUCM2 of the 6-well was distributed into two 24-wells (1 ml per well). Using a 3 ml sterile Pasteur pipette, with up and down slow movements, the aggregates were collected back to the 6-well (the total volume of medium per each well of the non-adherent 6-well plate should be 3 ml after finishing these transfers). The 6-well plate was incubated in a 20% O_2_ 5% CO_2_ 37 °C normoxic incubator on top of an orbital shaker at 60 r.p.m. placed within the incubator. Note that it is important not to significantly deviate from total 3 ml volume per each well of the 6-well plate to avoid clumping of the aggregates.

On day 4, 2 ml of medium was gently removed per well and replaced with 2 ml of pre-heated (37 °C in water bath) hEUCM2 with 30% FBS. The same procedure was repeated on day 5, refreshing with 2 ml of hEUCM2 with 30% FBS and put back to the same shaker/incubator setting. After 6 days, 2 ml of medium was gently removed per each well of the 6-well plate and replaced with 2 ml of pre-heated hEUCM2 with 50% FBS and placed back on the shaker in the normoxic incubator conditions. The same procedure was repeated at day 7, and placed back on the shaker in the normoxic incubator conditions. Cultures were ended at day 8 after aggregation.

hEUCM2 (ref. ^3^) was adapted from EUCM2 and is formulated as follows for human SEMs: advanced DMEM/F12 (Gibco, 21331-020), 1 mM GlutaMAX (Gibco, 35050061), 1% penicillin–streptomycin (Biological Industries, Sartorius 03-031-1B), 1× of ITS-X supplement (Thermo Fisher Scientific, 51500-056), 8 nM B-oestradiol (Sigma-Aldrich, E8875), 200 ng ml^–1^ progesterone (Sigma-Aldrich, P0130), 25 µM *N*-acetyl-l-cysteine (Sigma-Aldrich, A7250), 20–50% FBS (Sigma Aldrich F7524, heat-inactivated and filtered) as indicated in Fig. 2b, and optionally extra added 1 mg ml^–1^ d(+)-glucose monohydrate (J.T. Baker, 0113) (for example, add 500 mg per 500 ml medium). Culture medium was pre-heated for at least 1 h in a 37 °C water bath. FBS batches were tested and qualified for SEM assay by expanding V6.5 ES cells carrying an OCT4–GFP reporter and expanded for 3 passages on gelatin-coated plates and checked for >95% GFP^+^ signal by FACS analysis. We emphasize that the success of our human SEM protocol described above is not a single rare FBS batch-specific phenomenon, as three of four tested FBS batches used in the laboratory from two different vendors over the past 3 years of this ongoing project produced consistent results with some differences in efficiency between serum batches (successful FBS batches: (1) Sigma FBS F7524, lot no. 0001654682; (2) Biological Industries European-grade FBS 04-0071A lot no. 2004013 and (3) Sigma FBS F7524 lot no. 1664377) generated similar SEMs. To study the development of our human SEMs in further detail and with adequate reference, we mostly relied on the data from the Virtual Human Embryo Project based on the Embryo Carnegie Collection as the most detailed and relevant source to date^20,39^.

We emphasize that before starting to conduct co-aggregation experiments for human SEM generation as described herein, it is important to first test that the RCL and BAP(J) induction protocols are producing high-efficiency induction (as can be tested by FACS on day 3 for BAP(J) and day 6 for RCL cells). If RCL induction from naive HENSM PS cells that were expanded in hypoxia conditions on Matrigel is not showing a majority of PDGFRA^+^ cells (>65% PDGFRA^+^) at day 6, please be reminded that the RCL induction efficiency and quality mainly depends on the optimal initial cell seeding confluency before induction initiation, which can vary among human naive PS cell lines. It is recommended to make a seeding curve (assessed by FACS), starting with different initial cell numbers plated for induction and conduct FACS analysis on day 6. Also ensure that fresh RCL medium (less than 1 week old) is used and use B27 without insulin. If BAP(J) induction is not giving >85% ENPEP^+^TACSTD2^+^ TE-like cells at day 3, be reminded that BAP(J) induction quality and efficiency is also mainly dependent on the cell confluency, which can vary among human PS cell lines. It is recommended that a seeding curve is made, starting with different initial cell numbers plated for induction. Revise the cell confluency following TACSTD2 and ENPEP FACS analysis on day 3 of the BAP(J) induction regimen. Make sure not to keep BMP4 for more than the 24 h (only during the first day of the 3-day BAP(J) priming regimen). Once the two latter priming–induction protocols are producing adequate efficiency, as indicated above, then one can proceed to conducting co-aggregation experiments.

In our case, for the WIBR3 cell line grown in HENSM on Matrigel-coated plates for RCL induction, plating 8 × 10^5^ cells seeded onto each single 10-cm gelatin–MEF-coated Petri dish showed best results regarding RCL induction efficiency outcome at day 6, whereas in the case of the WIBR1 cell line, plating 2 × 10^6^ HENSM PS cells grown on Matrigel-coated plates onto a single gelatin–MEF-coated 10-cm plate showed optimal RCL induction results as measured by FACS on day 6. For the BAP(J) priming protocol from HENSM naive ES cells, for WIBR3 human ES cell HENSM naive cells grown on Matrigel, 1 × 10^6^ HENSM PS cells were plated per single 10 cm Matrigel-coated plate showed optimal results as measured by FACS on day 3 of the BAP(J) regimen. For the WIBR1 human ES cell line, seeding 2 × 10^6^ HENSM PS cells per 10 cm Matrigel-coated Petri dish produced optimal BAP(J) results as measured by FACS on day 3.

For initial training and calibration in conducting the human SEM protocol, we recommend stopping and evaluating initial experiments on day 6 and look within the generated aggregates for human SEMs (with amniotic-like cavity, bilaminar disc-like structure, hypoblast-like layer, YS-like compartment and TE-like outer layer). Then, once confident of success over multiple biological replicates, proceed to later stages up to day 8 of the protocol.

### Human blastoid generation and PALLY and PALY conditions

Human blastoids were generated according to a previously described method^15^ with a few modifications. WIBR3 human ES cells were grown in HENSM for at least 3 passages in feeder-free conditions (1% Matrigel- or Cultrex-coated plates) and were used for generating human blastoids. After 3 days of growth, naive ES cells were collected and counted, and 55 cells were seeded per microwell (total of 66,000 cells were seeded per 24-well in 1 ml of medium) AggreWell 400 24-well (Stem Cell Technologies, 34415) in N2B27-BSA aggregation medium supplemented with 10 µM of the ROCK inhibitor Y27632. The next day, medium was changed to PALLY, which consisted of N2B27 base with 1 µM of the MEK1/2 inhibitor PD0325901 (Axon Medchem, 1408), 1 µM of the TGFR inhibitor A83-01 (Axon Medchem, A83-01), 1 µM LPA (Tocris, 3854), human LIF (10 ng ml^–1^), and 10 µM of the ROCK inhibitor Y2763. This medium was repeated on day 2, but on day 3, the medium was changed for LY (1 µM LPA (Tocris, 3854) and 10 µM of the ROCK inhibitor Y27632) for another 48 h. Afterwards, human blastoids were manually selected and collected for further analysis. The entire procedure was conducted under 37 °C, 5% O_2_ and 5% CO_2_ conditions. PALY medium has the same composition of PALLY but without including LIF.

### Mouse SEM generation

Mouse SEM aggregations were made according to a previously described method^3^. Mouse animal experiments pertained only to mouse SEMs and were performed according to the Animal Protection Guidelines of the Weizmann Institute of Science and approved by the following Weizmann Institute IACUC (numbers 01390120-1, 01330120-2, 33520117-2). Embryo samples derived from the *Mus musculus* (mouse) ICR strain were used as reference controls for mouse SEM-related experiments. Male and female ICR mice (4–10 weeks old) were used for timed matings for natural embryo dissection that were used as controls. Mouse aggregation medium was also tested for human SEMs, but was found to be inappropriate. Mouse aggregation medium consisted of 1× DMEM (Gibco, 41965) supplemented with 20% FBS (Sigma), 1 mM GlutaMAX (Gibco, 35050061), 1% penicillin–streptomycin (Biological Industries, Sartorius 03-031-1B), 1% sodium pyruvate (Biological Industries, Sartorius 03-042-1B), 1% NEAA (Biological Industries, Sartorius 01-340-1B) and 0.1 mM β-mercaptoethanol (Thermo, 31350010).

### Morphological evaluation of human early embryonic development and efficiency calculations

Assessment of appropriate human development was performed through the careful analysis of available in utero histological embryo collections (predominantly the Carnegie collection), taking into account different tissue morphology and structure organization through different stages of development. Furthermore, available work on primate development was used as a reference for anatomical structure^8,40,41^, specific markers of each of the compartments was inferred from previous in vitro human development works^6,32^, in vitro differentiation protocols and primate existing databases^8^. Most of human histological descriptions and figures used for this paper are mentioned in the virtual human embryo website (https://www.ehd.org/virtual-human-embryo/)^20^ and available human embryology papers^18^.

All human in utero data and figures included in this study were made only from Carnegie collections after obtaining the appropriate copyright approvals. Only SEMs presenting all the previously defined features were considered as properly developed. The percentage of human SEMs generation was calculated on the basis of the number of properly developed structures observed per random fields of view at a specific time point during independent experiments while relying on immunofluorescence to corroborate the different lineage self-organization. Efficiency quantification was performed for SEMs according to the following three criteria at the indicated time points: (1) surrounded by a trophoblast-like layer, defined by the expression of CK7 or SDC1 in the perimeter of the SEM; (2) thepresence of epiblast-like and hypoblast-like compartments, defined by the expression of OCT4 and SOX17, respectively, in groups of cells inside the SEM; (3) the epiblast-like and hypoblast-like compartment forming a bilaminar disc-like structure along with the presence of amniotic-like and YS-like cavities, defined by the absence of the nuclear immunostaining signal in the central area of the epiblast-like and YS-like tissues.

The number of biological replicates (*N*) and number of samples per biological replicate sampled (*n*) are indicated in the figure legends and/or in the figure panels where relevant.

### Quantification and statistical analysis

Statistical analyses of real-time PCRs were performed using QuantStudio (v.1.3) software and visualized using GraphPad Prism 7. Visualization and statistical analyses of the cell numbers and SEM efficiencies were performed using Python (v.3.8.5) software with scipy (v.1.8.0) and seaborn (v.0.11.0) libraries. Boxplot graphs indicate medians with interquartile ranges, and the whiskers mark the distribution range. The barplots show average values plus the s.d. The dots mark individual numerical values used for visualization of the data distributions and analyses. The significant difference between two samples was evaluated using two-sided Mann–Whitney test for non-normally distributed data or two-tailed Student’s *t*-test as indicated per panel. A threshold of *P* < 0.05 was considered statistically significant.

### Whole-mount immunostaining

Human SEMs were collected using sterile plastic Pasteur-pipettes (Alex-Red), PE-3 ml/size 155 mm, SO P12201) and fixed in 4% paraformaldehyde EM grade (Electron Microscopy Sciences, 15,710) in PBS at room temperature for 1 h in glass spot plates (Corning, 722085). SEMs were then washed in PBS 3× for 5 min and permeabilized in PBS with 0.5% Triton X-100 (Sigma, 9002931)/0.1 M glycine (Sigma G7126) for 30 min. Blocking was performed in blocking solution (PBS/0.01% Tween20 (Sigma, 9005-64-5)/10% normal donkey serum (Jackson ImmunoResearch, 017000121)/0.1% BSA (Sigma, A7906)) for 1 h at room temperature, and incubated overnight at room temperature with primary antibodies, diluted in blocking solution.

Afterwards, SEMs were rinsed 3× for 5 min each in PBS/0.1% Triton X-100 and incubated with Alexa Fluor (488, 568 and/or 647)-conjugated donkey secondary antibodies (Jackson ImmunoResearch) diluted in blocking solution (1:200) for 2 h. The samples were counterstained with DAPI for nuclei (1 mg ml^–1^ in PBS) for 10 min and were washed three times with PBS for 5 min each. When membrane staining was required, rhodamine phalloidin (Invitrogen, R415) or wheat germ agglutinin (Invitrogen, W21404) was added in a 1:200 dilution with the secondary antibodies.

The following antibodies and dilutions were used for immunofluorescence: mouse monoclonal anti-Oct3/4 (clone C-10) (Santa Cruz, SC-5279) 1:100; rabbit polyclonal anti-Oct3/4 (clone H-134) (Santa Cruz, SC-9081) 1:100; goat polyclonal anti-Sox17 (R&D, AF1924) 1:100; rabbit monoclonal anti-CK7 (Abcam, ab181598) 1:200; rabbit monoclonal anti-CK7 (Abcam, ab68459) 1:200; goat polyclonal anti-Gata3 (R&D, AF2605) 1:100; rabbit monoclonal anti-syndecan1 (Abcam, ab128936) 1:400; mouse monoclonal anti-Cdx2 (Biogenex, MU392A-UC) 1:200; rabbit monoclonal anti-phospho-ezrin (Cell Signaling, 3726) 1:400; rabbit monoclonal anti-Brachyury (D2Z3J) (Cell Signaling, 81694) 1:100; goat polyclonal anti-Cer1 (R&D, AF1075) 1:100; rabbit monoclonal Nanog (Abcam, ab109250) 1:100; mouse monoclonal anti-PKC zeta antibody (H-1) (Santa Cruz, SC-17781) 1:200; mouse monoclonal anti-podocalaxyn (clone 222328) (R&D, MAB1658) 1:200; rabbit polyclonal anti-Gata4 (Abcam, ab84593) 1:100; mouse monoclonal anti-vimentin (Abcam, ab8978) 1:100; rabbit monoclonal anti-BST2/Tetherin antibody (EPR20202-150) (Abcam, ab243230) 1:100; rabbit monoclonal anti-hCG beta (5H4-E2) (Abcam, ab9582) 1:200; rabbit monoclonal anti-Gata6 (clone D61E4) (Cell Signaling, 5951) 1:100; rabbit monoclonal anti-Islet1 (EP4182) (Abcam, ab109517) 1:100; mouse monoclonal anti-TFAP2a (AP-2α) (3B5) (Santa Cruz, SC-12726) 1:100; goat polyclonal anti-Sox2 (R&D, AF2018) 1:200; rabbit polyclonal anti-Dnmt3l (Imgene, IMG-6804A) 1:100; goat polyclonal anti-Otx2 (R&D, AF1979) 1:200; mouse monoclonal anti-Stella (D-5 clone) (Santa Cruz, SC-376862) 1:100; rabbit monoclonal anti-Blimp1/PDRI-BF1 (clone C14A4) (Cell Signaling, 9115) 1:100; and goat polyclonal anti-FoxF1 (R&D, AF4798) 1:100.

### Immunofluorescence

Cells were fixed in 4% paraformaldehyde in PBS at room temperature for 10 min. Samples were then washed 3× in PBS, permeabilized in PBST (PBS with 0.1% Triton X-100) for 10 min, blocked in PBS/0.05% Tween/5% FBS/1% BSA for 1 h and incubated with primary antibodies diluted in blocking solution at 4 °C overnight. Subsequently, cells were washed in PBS/0.05% Tween (3×, 5 min each) and incubated with Alexa Fluor (488, 568 and/or 647)-conjugated secondary antibodies (Jackson ImmunoResearch) diluted in blocking solution (1:200). Samples were counterstained with 1 μg ml^–1^ DAPI for 10 min at room temperature, washed with PBS 3× (5 min each) and mounted on slides with Shandon Immuno-Mount (Thermo Scientific) or kept in PBS.

The following antibodies and dilutions were used for cell immunofluorescence: rabbit monoclonal anti-BST2/Tetherin antibody (EPR20202-150) (Abcam, ab243230) 1:100; goat polyclonal anti-Sox17 (R&D, AF1924) 1:100; rabbit polyclonal anti-Gata4 (Abcam, ab84593) 1:100; goat polyclonal anti-FoxF1 (R&D, AF4798) 1:100; goat polyclonal anti-Gata3 (R&D, AF2605) 1:100; mouse monoclonal anti-Cdx2 (Biogenex, MU392A-UC) 1:200; goat monoclonal Tfap2c (R&D, AF5059) 1:200; rabbit monoclonal anti-syndecan 1 (Abcam, ab128936) 1:400; rabbit monoclonal anti-hCG beta (5H4-E2) (Abcam, ab9582) 1:200; rabbit monoclonal anti-CK7 (Abcam, ab68459) 1:200; mouse monoclonal anti-vimentin (Abcam, ab8978) 1:100; mouse monoclonal anti-Oct3/4 (clone C-10) (Santa Cruz, SC-5279) 1:100; rabbit polyclonal anti-Oct3/4 (clone H-134) (Santa Cruz, SC-9081) 1:100; goat polyclonal Gata6 (R&D, AF1700) 1:200; rabbit monoclonal Gata6 (Cell Signaling, 5951) 1:200; goat polyclonal nidogen 2 (R&D, AF3385) 1:100; and rabbit monoclonal anti-Gata2 (EPR2822) (Abcam, ab109241) 1:200.

### Flow cytometry

Flow cytometry analyses were done on a BD FACS-Aria III. Cells were collected using TrypLE and washed once with PBS. Cells were then incubated for 30 min with conjugated primary antibodies (5 µl) with 100 µl PBS/0.5% BSA. The following primary antibodies were used: mouse monoclonal TROP2-488 labelled (R&D, FAB650G); mouse monoclonal TROP2-PE labelled (R&D, FAB60P); mouse monoclonal CD249 (ENPEP)-BV421 labelled (BD, 744872); rat monoclonal anti-mouse CD140a (PDFGRa)-PE/Cy7 labelled (BioLegend, 135912); mouse monoclonal anti-human CD140a (PDFGRa)-PE/Cy7 labelled (BioLegend, 323508); and mouse monoclonal anti-human CD140a (PDFGRA)-APC labelled (BioLegend, 323512). FSC and SSC singlets were gated, and only single cells were considering for all analyses. An unstained control was used to determine the negative/positive populations for all antibodies, ensuring that nearly 100% of the unstained population was allocated on the negative area of the histogram/dot plot. Flow cytometry data were analysed using FlowJo (v.10.7). Supplementary Fig. 17 demonstrates FACS gating strategies used in this study.

### RNA extraction and RT–PCR analysis

Total RNA was isolated using a RNeasy mini kit (Qiagen) following the manufacturer’s instructions. 1 μg of total RNA was reverse transcribed using a High-Capacity Reverse Transcription kit (Applied Biosystems). RT–PCR was performed in triplicate technical wells for each of the sample included per each gene, using SYBR Green PCR master mix (Qiagen) and run on a ViiA7 platform (Applied Biosystems). Values were normalized to actin and/or GAPDH and/or HPRT and/or RPL3 across all experiments. The data are presented as relative expression values compared with the reference sample using the ΔΔCT method. Samples were visualized using Prism (v.7), plotting the mean value with s.d. A list of RT–PCR primers is provided in Supplementary Table 3.

### Confocal microscopy

The immunofluorescence images were acquired using a Zeiss LSM 700 and a Zeiss LSM 800 inverted confocal microscope, both equipped with 405, 488, 555 and 635 nm solid-state lasers using a Plan-Apochromat ×20 air objective (numerical aperture 0.8) or an EC Plan Neofluar ×10 air objective (numerical aperture 0.3). Images of the trophoblast cell surface were acquired using a C-Apochromat ×40 water objective (numerical aperture 1.2) using a LSM 800 microscope. For a detailed description of the imaging parameters, see Supplementary Table 4. Confocal 3D images and maximum-intensity projections were processed using Fiji (v.1.52p or 1.53t)^42^, Zen 2 blue edition software 2011 or ZEN Z3.5 (Zeiss), and Adobe Illustrator 2023 CC.

### Light-sheet microscopy

The immunofluorescence images were acquired using a Zeiss Z7 light-sheet microscope equipped with 405, 488, 561 and 638 nm lasers, using a single water ×20 Plan-Apochromat (numerical aperture 1.0) detection objective (Zeiss) and two air ×10 Plan-Apochromat (numerical aperture 0.2) illumination objectives (Zeiss). Before imaging, the sample was mounted in a glass capillary filled with 1% low-melting temperature agarose. Following solidification, the agarose was pulled out of the capillary with a custom plunger to hang into the imaging chamber filled with PBS.

A single sample was imaged each time from several angles using Multiview acquisition. Light-sheet volumes along the *z* axis were acquired in a dual scanning mode using a pivot scan. Light-sheet thickness was set to 3.77 µm, and laser power in the 1–50% range was applied. The frame size was 1,920 × 1,920 px, and the exposure time was 50 ms. The light sheets for left-side and right-side illuminations were adjusted independently inside the sample volume for each channel based on the signal intensity in the focal plane of the detection lens. See also Supplementary Table 4.

Light-sheet image processing was performed using Zen 3.5 software. Dual side images were fused based on the maximum-intensity signal. Multiview fusion was performed using interactive registration of the brightest channel (typically DAPI) or each channel independently in front and side views. The ‘Blending’ parameter was typically set to 50, and the intensities of the fused images were averaged (‘Method: Mean Fusion’). The image deconvolution was applied for single-view images before their fusion in the ‘Fast Iterative’ or ‘Constrained Iterative’ settings.

### Confocal imaging of human SEMs or aggregates

To assess the quality of the experiment and to select putative SEMs for further imaging, an overview of the majority of the SEMs was gathered using tiled scanning implemented in Zeiss LSM 700 and LSM 800 inverted confocal microscopes. SEM(s) were mounted in a 35 mm glass-bottom dish (Mattek, P35G-1.5-14-C) covered with PBS. To generate the overview images, a ×10 EC Plan Neofluar air objective (0.3 NA) or a ×20 Plan-Apochromat air objective (1.0 NA) were used with Zen software (black edition; Zeiss).

#### Confocal imaging of multiple SEMs or aggregates

After mounting the sample, the image acquisition parameters were established for each of the assessed wavelengths (405, 488, 568, 647 nm) according to the used secondary antibodies. Tiled images were obtained to sample a significant portion or most of the aggregates. As aggregates are dispersed in different directions and angles, *z*-stacks were used to better understand their structure. The efficiency of the experiment was calculated after gathering the overview image (see below). In addition, the images of individual SEMs or aggregates was taken by moving the objective to the recorded positions within a tiled scan. For light-sheet microscopy, the SEMs were examined and picked with a mouth pipette (aspirator tube; Sigma, A5177) connected to a thin glass capillary pulled from a glass microlitre capillary (Blaubrand intraMark, 708744) with an inner diameter greater than the diameter of the SEM. Under the binocular view, the SEM of interest was taken from the imaging plate while trying to avoid disturbing other aggregates and without generating air bubbles. The picked SEM was placed in a new drop of PBS on a Petri dish for further analysis.

#### Confocal imaging of an individual SEM or aggregate

The previously collected overview images of the experiment were used to select the SEMs based on the microscope stage position. Note that to maintain the same stage position, the SEMs should not be moved or disturbed during the entire imaging process. Hence, for every single imaging session, a new overview image was required. The stage was redirected to one of the chosen positions and switched to a higher magnification objective and acquisition parameters for each channel were recalibrated. Digital zoom and rotation was used to centre the SEM as desired. It was important to ensure that the entire SEM fit into the imaging frame when examining morphology. If 3D volumes were required, the beginning and end frames were set to cover the SEM, and the slice spacing was selected according to the desired sampling along the *z* axis. Alternatively, the laser intensity was adjusted (implemented in Zeiss LSM) to reduce decay of the signal along the *z* axis. Images were then acquired.

#### Quantification of experimental efficiency

Quantification of the efficiency of the experiment was performed on the basis of the overview tile-scanned images (see the section ‘Confocal imaging of multiple SEMs or aggregates’). The cell-type markers for immunostaining were selected before the experiment depending on the defined efficiency criteria. For assessing the efficiency and quality of the experiments, we used markers for the main three lineages: epiblast-like (OCT4 and SOX2); YS-like (SOX17); ExEM-like (VIM1 and FOXF1) and trophoblast-like (GATA3, CK7 or SDC1). Afterwards, the following steps were taken: (1) Fiji software was used to open the previously acquired overview image; (2) adequate colours were assigned to each channel; the Cell Counter tool was used to manually count the total number of aggregates that fit entirely into the image; (4) the aggregates that met the desired criteria were selected using another Cell Counter tool. The efficiency of the experiment was calculated by dividing the number of adequate SEMs by the total number of structures. This value was multiplied by 100 to obtain percentage values.

#### Picking human SEMs under by confocal microscopy

To select stained SEMs for further analysis and imaging, picking them with the help of confocal microscopy while imaging was our current best solution because the morphology of human SEMs is difficult to discriminate by bright-field microscopy and long periods of training are required. In brief, confocal microscopy was used to localize the chosen SEM (the previously gathered overview helped guide the stage to the right direction). The live view was used to ensure that the correct SEM was selected. Using the binocular view and a mouth pipette (with a previously pulled glass capillary), the structure was taken from the plate while trying to avoid disturbing other structures as little as possible and without generating bubbles. The picked SEM was placed in a new drop of PBS on a Petri dish for further analysis.

### Electronically controlled ex utero roller culture platform

Human SEMs can be kept in an ex utero electronically controlled roller culture platform after day 6. The system provides a continuous flow of oxygenating gas^43^ through an electronic gas-modulating unit (designed by J.H.H. and assembled and sold by Arad Technologies, Hanna Lab model no. 1) that is adapted to the roller culture unit from B.T.C. Engineering (Cullum Starr Precision Engineering), as previously described^3,43^. On day 7, all human SEMs from a well of a 6-well plate were picked and transferred to glass culture bottles (50–100 aggregates per bottle) containing 4 m of fresh hEUCM2 50% FBS. The bottles were placed on the rolling culture system, rotating at 30 r.p.m. at 37 °C and continuously gassed with an atmosphere of 21% O_2_, 5% CO_2_ at 6.5–8 psi, which produces a gas input into the humidifier bottle of 1 l min^–1^. Bottles were kept inside glass culture bottles rotating on a spinning wheel allocated inside a precision incubator system (BTC01 model with gas bubbler kit, B.T.C. Engineering, Cullum Starr Precision Engineering) (BTC 04). The gas flowed into the gas mixing box at 0.5 psi and from the gas mix box through the inlet into the humidifier water bottle (designed by J.H.H. and manufactured as a modifier for the incubator part by Arad Technologies, and this upgrade is essential for the incubator set-up) at 6.5–8 psi to produce an input flow to the humidifier of 1 l min^–1^, and then to the inside of the bottles in the rotating drum. The bubble rate was adjusted using the valve on the lid of the water humidifier bottle to the first point where continuous bubbling is observed, which generally corresponded to 0.06–0.1 psi output after the humidifier water bottle to produce around 60–100 ml min^–1^ gas flow output from the humidifier bottle. The rate of bubbles created inside an outlet-test tube filled with water was used to generate a gas output of 0.06–0.1 psi and a gas flow of about 60–100 ml min^–1^ into the wheel compartment. A black cloth or large diaper was used cover the incubator to provide protection against phototoxicity. A volume of 2–3 ml of hEUCM2 with 50% FBS was used in the roller culture for human SEMs at these stages.

### Chromium 10x scRNA-seq

To further validate and examine the milieu of cell types present in the human SEMs generated herein in a more unbiased manner, we performed a single-cell transcriptomics analysis by Chromium 10x scRNA-seq. Human SEMs grown ex utero were manually selected on the basis of morphological criteria that matched representative structures shown in Fig. 2c between days 4 and 8 and collected for scRNA-seq (Supplementary Table 1) at days 4, 6 and 8 (from two biological replicates run in parallel to reduce intrinsic variability per each time point) using the Chromium Next GEM Single Cell 3′ platform (v.3.1). All human SEMs analysed by scRNA-seq were generated by co-aggregating WT WIBR3 naive ES cell lines grown in HENSM with RCL-induced or BAP(J)-induced WT cells. At day 4, around 80 human SEMs were pooled into one lane of a 10x chromium chip, whereas at day 6 and 8, a pool of about 50 SEMs were sequenced per lane. All SEM samples were processed, including extra-embryonic compartments without any dissection. SEMs were dissociated using trypsin-EDTA solution C (0.05%) for 10 min (Biological Industries, 030501B). Trypsin was neutralized using medium with 10% FBS, and cells were washed and resuspended in 1× PBS with 400 µg ml^–1^ BSA. The cell suspension was filtered through a 100 µm cell strainer to remove cell clumps. A cell viability of at least 90% was determined by trypan blue staining for all samples. Cells were diluted to a final concentration of 1,000 cells per µl in 1× PBS with 400 µg ml^–1^ BSA. scRNA-seq libraries were generated using a 10x Genomics Chromium v.3.1 Dual Index system (5,000 cell target cell recovery) and sequenced using an Illumina NovaSeq 6000 platform according to the manufacturer’s instructions.

### 10x scRNA-seq analysis for SEM, RCL and BAP(J) samples

10x Genomics data analysis was performed using Cell Ranger 7.1.0 software (10x Genomics) for pre-processing of raw sequencing data and with Seurat 4.3.0 for downstream analysis. To filter out low-expressing single cells, possible doublets produced during the 10x sample processing, or single cells with extensive mitochondrial expression, we filtered out cells with fewer than 1,000 expressing genes, more than 8,000 expressing genes and more than 15% mitochondrial gene expression. We analysed around 4,000–8,000 cells from pooled high-quality SEMs. After quality control and strict filtering, a total of 12,190 single cells were used for subsequent analyses (Supplementary Fig. 15a–c). Seurat integrated analysis and anchoring of all individual samples were performed and then normalized by log-normalization using a scale factor of 10,000. The top 2,000 variable genes were identified using the variance stabilizing transformation method and subsequently scaled and centred. Principal component analysis was performed for dimensional examination using the ‘elbow’ method. The first ten dimensions showed the majority of data variability. Therefore, UMAP dimensional reduction was performed on the first ten dimensions in all samples. Clusters were detected using the Seurat Find Clusters function, with a resolution parameter of 0.5. Dotplots describing the expression and prevalence of specific genes were generated using the Seurat DotPlot() function. Projection of selected genes on SEM UMAP was generated using the Seurat FeaturePlot() function. Heatmaps were generated using the Seurat DoHeatmap() function or with R pheatmap package (v.1.0.12). Analysis code is available at GitHub (https://github.com/hannalab/Human_SEM_scAnalysis). We note that although ExEM-like cell cluster 8 cells express CER1, DKK1 and LHX1 AVE markers, they lack other key VE/AVE markers such as SOX17 and APOA1 and were therefore not annotated as AVE-like but rather as ExEM-like cells as they predominantly express a mesenchymal signature (Extended Data Fig. 13a). The latter is consistent with CER1, DKK1 and LHX1 being co-expressed in ExEM cells as detected in primates^8^ and gastrulating human embryo datasets^33^. The fact that PGC-like cells did not form their own cluster or subcluster probably results from their relative scarcity within SEMs, as was observed in mouse SEMs^3^.

Integration of scRNA-seq data from the RCL starting population with published scRNA-seq day 6 primitive endoderm RACL based conversion^9^ (Extended Data Fig. 1f) was performed using the Seurat v.3 integration standard workflow^44,45^. Before integration, datasets were normalized, and the top 2,000 most variable genes were selected. Integration anchors were identified using the FindIntegrationAnchors function with default arguments, incorporating all available parameters and data across features. An integration-based UMAP was constructed using the runUMAP function with dimensions ranging from 1 to 10 for visualization. Similar analyses were performed to integrate scRNA-seq of the BAP(J) starting population with naive trophectoderm and naive cytotrophoblasts^14^ (Extended Data Fig. 3g) and scRNA-seq of SEM ExEM-like cell populations with the previously published time course dataset of naive to TE cell/TS cell/ExEM cell dataset^9^ (Extended Data Fig. 13f).

### HENSM naive and primed 10x single-cell multiomics library prep, sequencing and computational analysis

HENSM scRNA-seq (Supplementary Fig. 3) data were processed alongside single-cell ATAC–seq data measured from the same cells (multimodal strategy) in a protocol described below: LIS49 and WIBR3 human ES cells were cultured for 3 passages in HENSM and primed conditions on Matrigel-coated plates. Cell nuclei were isolated using the protocol provided by 10x genomics (CG000365), aiming for around 6,000 nuclei. 10x Genomic library preparation was performed using the protocol provided by 10x Genomics (CG000338) and sequenced with 2 units of NovaSeq SP sequencing system (100 cycles). For computational analyses, sequencing outputs were demultiplexed using Cell-Ranger-arc (v.2.0; 10x Genomics) mkfastq command, and counts were estimated using Cell-Ranger count software. ATAC and RNA data were pre-processed separately and filtered using the Seurat 4.0 R package. For ATAC data, cells with very high (>10,000) or very low (<1,000) numbers of overlapping peaks were filtered out, as well as cells with high (>3) nucleosome signal scores or low TSS enrichment scores (<2). For RNA data, cells with low numbers of features (bottom 10%), high number of UMIs (top 10%) or high mitochondrial percentage (top 10%) were filtered out. Doublet cells were also assessed by RNA data using scDblFinder (v.1.6). Cells that passed both RNA and ATAC filtering parameters were further examined together. Seurat integrated analysis and anchoring of RNA data were performed and then normalized by log-normalization using a scale-factor of 10,000. The top 5,000 variable genes were identified using the variance stabilizing transformation method and subsequently scaled and centred. UMAP was performed on the first 30 principal components. ATAC data were normalized by term frequency inverse document frequency (TF-IDF), and partial singular value decomposition (SVD) was performed using RunSVD by Signac (v.1.6.0). Batch effect correction was performed using RunHarmony by harmony package (https://portals.broadinstitute.org/harmony/index.html), whereas the cell line type was excluded from the correction, and reduction was based on latent semantic indexing (LSI) produce by SVD. UMAP was performed on the harmony (v.3)-corrected 2:50 dimensions. Construction of the weighted nearest neighbour was perform using FindMultiModalNeighbors by Seurat based on RNA PC1:30 and ATAC harmony 2:50.

### Pseudotime analysis

Pseudotime analysis of scRNA-seq data was done using monocle3 R package (v.1.3.1) over the relevant Seurat objects. A heatmap of top differentially expressed genes is presented with the order determined by the pseudotime using pheatmap R package (v.1.0.12).

### Projection on the Human Embryo Reference Compendium

The human embryo reference^27^ was built by integrating previously published datasets consisting of 6 human embryonic datasets spanning early in vitro cultured human blastocysts^28^, 3D in vitro cultured human blastocysts until pre-gastrulation stages^29–31^, and a CS7, 16–19 d.p.f. human gastrula^33^ using fastMNN from batchelor (v.1.6.2)^46^ as recently described^27^. The raw counts for cells of human SEMs were aggregated within neighbourhood nodes as calculated using Milo^47^, resulting in 945 representative neighbourhoods, followed by projecting the summed counts matrix onto the assembled human embryo reference using MNN (mutual nearest neighbour correction)^46^, followed by stabilized UMAP projection using the umap_transform function from R package uwot (v.0.1.14)^48^ (Fig. 6e). Milo neighbourhoods for projects was chosen (rather than scMAP tool to map single-cells onto existing datasets) given the noise, and after verifying that the neighbourhoods were approximately homogenous in their cell-type composition. Note that we have checked the assigned cluster information for representative neighbourhoods and related neighbourhoods. As shown in Supplementary Fig. 16a, 97.5% and 83.7% of the related neighbourhoods have the same cell identity with representative neighbourhoods based on lineage information and Seurat cluster information, respectively. Thus, validating that the neighbourhoods are highly homogenous in their cell-type composition. Alluvial plot was used to compare the cell-type annotations of representative node SEM cells to the predicted identities obtained from scMAP^32,49^ (Supplementary Fig. 16b). The prediction results for SEM cells (after aggregation using neighbourhood methods) are shown here. The majority of SEM-epiblast-like, SEM-syncytiotrophoblast-like and SEM-YS/hypoblast-like cells were identified. However, the SEM Amnion-like and ExEM-like cells were determined as unassigned, which is probably due to the low number of embryonic reference cells for these cell types and the relatively low sequencing depth, as previously described^33^.

### Mouse extra-embryonic annotation analysis

RHOX5^+^ cells (>1 counts) were chosen for the analysis of extra-embryonic tissue. The cells were annotated on the basis of marker genes as previously conducted^25^, such that if at least 4 markers (3 in the case of SpA-TGC and SpT-Gly) were expressed (>0 counts), the cell was annotated in that category. In total, 26% of the annotated cells were annotated by multiple categories. The following markers were used as previously described^25^: chorion (Irx4, Esx1, Id1, Id3, Phlda2 and Klhl13); chorion progenitors (Sox3, Dusp6, Nat8l, Bmp4, Sox2, Esrrb and Eomes); intermediate chorion (Ascl2, Fgfr2, Cited1, Gjb3, Ndrg1, Irx2 and Irx3); uncommitted Ecto-placental cone (EPC) (Chsy1, Gjb3, Krt19, Lgals1, Cald1 and Ctsl); SpA-TGC (Ctla2a, Pecam1, Ramp3, Igfbp7 and Nos3); SpT-Gly (Dlx3, Car2, Ncam1, Pcdh12 and Tpbpa); TGC-progenitors (Adm, Fosl1, Hand1, Trpm5, Maged2 and Prl5a1); and p-TGC (Star, Serpinb9d, Hsd3b6, Rhox6 and Cts7). R ggplot was used to generated scatter plots along with geom_smooth(method=”lm”).

### Mouse and Human IGV analysis

Bulk ATAC–seq and RNA-seq profiles in the proximity of selected genes (*GATA3*, *GATA4* and *GATA6*) are presented using Broad IGV genome browser (v.2.16.2). Mouse datasets were taken from published datasets^50–52^ and from a Gene Expression Omnibus dataset (https://www.ncbi.nlm.nih.gov/geo/query/acc.cgi?acc=GSE181053), as were human datasets^4,12,53^. Mouse enhancers were taken from a previously published study^51^, and human enhancers were taken from GeneHancer^54^. Open regions that overlapped with a promoter or an exon were excluded from the analysis. Potential enhancers in these regions were manually curated.

### Reporting summary

Further information on research design is available in the Nature Portfolio Reporting Summary linked to this article.

## Online content

Any methods, additional references, Nature Portfolio reporting summaries, source data, extended data, supplementary information, acknowledgements, peer review information; details of author contributions and competing interests; and statements of data and code availability are available at 10.1038/s41586-023-06604-5.

### Supplementary information

Supplementary Information Supplementary Figs. 1–17 and legends, supplementary introduction, supplementary discussion and supplementary references.

Reporting Summary

Supplementary Table 1 Human SEM scRNA-seq analysis related gene expression list. Top markers (average log_2_(fold change )> 0.25 and one-sided Wilcoxon text *P* value < 0.01) of each of the 13 cell clusters in human SEMs, as identified by Seurat package using one-sided Wilcoxon test.

Supplementary Table 2 Cell annotation of human SEM cells. a, Cell annotation of human SEM. b, List of CTb-like and STb-like annotated cells. c, Normalized gene expression of top 50 differentially expressed genes that are upregulated in CTb-like compared with STb-like cells (Extended Data Fig. 12e).

Supplementary Table 3 PCR primers used in this study. Primer names, DNA sequences from 5′ to 3′ end, and the cited reference (when applicable).

Supplementary Table 4 Summary of the microscopy parameters used for imaging in this study. The spreadsheet provides information of the type of the microscope, used detection objectives, laser lines and the voxel size for acquisition of the images published herein.

Supplementary Video 1 3D reconstruction of a day 8 human SEM (WIBR3 cell line). Immunofluorescence for epiblast-like (OCT4, cyan), hypoblast-like (SOX17, yellow), trophoblast-like (CK7, magenta) compartments, and nuclei (DAPI, white). 0–8 s, 3D view of the outer trophoblast-like layer with enlarged multinuclear cells. 8–22 s and 39–42 s, 3D segmentation of the epiblast-like (cyan) and hypoblast-like (yellow) structures with DAPI. 22–38 s, inner SEM structure comprising a bilaminar disc-like structure with amnion-like and YS-like compartments, surrounded by connective tissue and trophoblast-like cells. Immunofluorescence signal and tissue segmentation are outlined. Acquisition with Z7 microscope and processing with Imaris (v.10.0.1).

Supplementary Video 2 3D reconstruction of a day 8 human SEM (WIBR3 cell line). Immunofluorescence for epiblast-like (OCT4, cyan), YS-like (SOX17, yellow), trophoblast-like compartment (CK7, magenta). 0–8 s and 19–21 s, 3D view of the outer trophoblast-like layer. 8–18 s, slicing through the 3D volume showing the inner cellular structure of the SEM comprising a bilaminar disc-like structure, amnion-like and YS-like compartment with the inner cavity. 3D rendering of the thresholded immunofluorescence signal. Acquisition with Z7 light-sheet microscope and processing with Imaris (v.10.0.1).

Supplementary Video 3 3D reconstruction of a day 6 human SEM (WIBR1 cell line). Immunofluorescence for epiblast-like (OCT4, cyan), YS-like (SOX17, yellow), trophoblast-like compartment (CK7, magenta), and nuclei (DAPI, white). 0–10 s, 3D view of the outer trophoblast-like layer. 10–50 s, 3D segmentation of the epiblast-like (cyan) and hypoblast-like cells (yellow) with DAPI maximum projection. 21–41 s, slicing through the 3D volume showing the inner structure of the SEM comprising a bilaminar disc-like structure with early amnion-like compartment, connected to the outer trophoblast-like layer. Immunofluorescence signal and tissue segmentation are outlined. Acquisition with Z7 light-sheet microscope and processing with Imaris (v.10.0.1).

Supplementary Video 4 3D reconstruction of a human SEM at day 6 demonstrating pro-amniotic-like cavity formation within the epiblast-like compartment. 3D reconstruction of the human SEM at day 6 shows its 3D morphology (0–12 s) and beginning of the proamniotic-like cavity formation within the epiblast-like compartment (12–18 s). Immunofluorescence for OCT4 (epiblast, cyan), F-acting (red) and nuclei (DAPI, white). The image was acquired with a Zeiss LSM 800 microscope and processed with Imaris (v.10.0.0).

Supplementary Video 5 3D reconstruction of a human SEM at day 8 shows formation of embryonic disc-like structure and amnion-like compartment. 3D reconstruction of the embryonic disc-like structure (SOX2, cyan) and amnion-like compartment (TFAP2A, magenta) in a day 8 human SEM. 3D segmentation of epiblast-like tissue (cyan) and amnion-like tissue (pink) is denoted as the semi-transparent outline together with the corresponding immunofluorescence signal. 17–21 s, human SEM epiblast-like compartment has a disc shape. The image was acquired with Z7 light-sheet microscope and processed with Imaris (v.10.0.0).

Supplementary Video 6 3D reconstruction of a human SEM at day 6 shows YS-like morphology and polarity. 3D reconstruction of a human day 6 SEM showing YS-like structure (marked by SOX17, yellow). 5–18 s, zoom into the visceral and parietal YS-like cells having columnar and squamous cell shape, respectively. The cells exhibit apical polarization (aPKC, green). F-actin (red), nuclei (DAPI, white). The image was acquired with a Zeiss LSM 700 microscope and processed with Imaris (v.10.0.0).

Supplementary Video 7 3D reconstruction of a human SEM at day 8 shows ExEM-like cell integration underneath the YS-like structure. 3D reconstruction of a human SEM at day 8 showing ExEM-like cells marked by expression of VIM (red) and located predominantly underneath the YS-like structure (yellow); OCT4 (epiblast, cyan), nuclei (DAPI, grey). 3D rendering of the thresholded immunofluorescence signal. The image was acquired with a Zeiss LSM 700 microscope and processed with Imaris (v.10.0.0).

Supplementary Video 8 3D reconstruction of human SEM at day 6 shows development of the syncytiotrophoblast-like layer with lacunae-like structures. 3D reconstruction of a human SEM at day 6 showing development of the STb-like layer, expressing both SDC1 (magenta) and HCGB (green). The STb-like compartment forms multiple lacunae-like structures. F-actin (red), nuclei (DAPI, grey). 11–19 s, slicing through the 3D volume showing the inner structure of the multiple trophoblast lacunae-like structures. The image was acquired with a Zeiss LSM 700 microscope and processed with Imaris (v.10.0.0).

### Source data

Source Data Fig. 2

Source Data Fig. 3

Source Data Fig. 4

Source Data Fig. 5

Source Data Extended Data Fig. 1

Source Data Extended Data Fig. 3

Source Data Extended Data Fig. 5

Source Data Extended Data Fig. 7

Source Data Extended Data Fig. 8

Source Data Extended Data Fig. 10

Source Data Extended Data Fig. 12

## Data Availability

All newly generated scRNA-seq and 10x Chromium Single Cell Multiome ATAC and gene expression data have been deposited into the Gene Expression Omnibus database with the identifier GSE239932. GSE numbers and references are indicated for all other previously published and publicly available scRNA-seq and ATAC–seq data are indicated. Any other data are available upon request. All other information required to re-analyse the data reported in this work is available upon request from the corresponding author. Source data are provided with this paper.

## Code availability

The custom code generated in this study is provided at GitHub: https://github.com/hannalab/Human_SEM_scAnalysis. The custom code was not essential to the main conclusions of this study.
