# Supplementary material for: Complete human day 14 post-implantation embryo models from naive ES cells
Source: Nature. 2023 Sep 6;622(7983):562–73. doi: 10.1038/s41586-023-06604-5 (PMC10584686; doi:10.1038/s41586-023-06604-5)
Supplement: Supplementary file 1 — Supplementary Figs. 1–17 and legends, supplementary introduction, supplementary discussion and supplementary references. [file 41586_2023_6604_MOESM1_ESM.pdf]

nature portfolio

https://doi.org/10.1038/s41586-023-06604-5

Supplementary information

Complete human day 14 post-implantation embryo models from naive ES cells

In the format provided by the authors and unedited

Supplementary Information (SI) Guide

i) Supplementary Table Legends

ii) Supplementary Video Legends

iii) Supplementary Figures and Legends

iv) Supplementary Introduction

v) Supplementary Discussion

vi) Supplementary References

Supplementary Table Legends

Supplementary Table 1. Human SEM scRNA-seq analysis related gene expression list.
Top markers (average log2(Fold-change)>0.25 and one-sided Wilcoxon text p-value < 0.01) of each of the 13 cell clusters in human SEMs, as identified by Seurat package using one-sided Wilcoxon test.

Supplementary Table 2. Cell annotation of human SEM cells.
a. Cell annotation of human SEM. **b**. List of CTb-like and STb-like annotated cells. **c**. Normalized gene expression of top 50 differentially expressed genes that are upregulated in CTb-like compared to STb-like cells (**Extended Data Fig. 12e**).

Supplementary Table 3 - PCR primers used in this study.
Primer names, DNA sequences from 5' to 3' end, and the cited reference (when applicable).

Supplementary Table 4. Summary of the microscopy parameters used for imaging in this study. The spreadsheet provides information of the type of the microscope, used detection objectives, laser lines, and the voxel size for acquisition of the images published herein.

Supplementary Video Legends

Supplementary Video 1. 3D reconstruction of the day 8 human SEM (WIBR3 cell line).
Immunofluorescence for epiblast- (OCT4, cyan), hypoblast- (SOX17, yellow), trophoblast-like (CK7, magenta) compartments, and nuclei (DAPI, white). 0 – 8 sec, 3D view of the outer trophoblast-like layer with enlarged multinuclear cells. 8 – 22 sec and 39 – 42 sec, 3D segmentation of the epiblast- (cyan) and hypoblas-like (yellow) structures with DAPI. 22 sec – 38 sec, inner SEM structure comprised of bilaminar disk-like structure with amnion-like and yolk sac-like compartments, surrounded by the connective tissue and the trophoblast-like cells. Immunofluorescence signal and tissue segmentation are outlined. Acquisition with Z7 microscope and processing with Imaris v10.0.1.

Supplementary Video 2. 3D reconstruction of the day 8 human SEM (WIBR3 cell line).
Immunofluorescence for epiblast- (OCT4, cyan), yolk sac- (SOX17, yellow), trophoblast-like compartment (CK7, magenta). 0 – 8 sec and 19 – 21 sec, 3D view of the outer trophoblast-like layer. 8 sec – 18 sec, slicing through the 3D volume showing the inner cellular structure of the SEM comprised of bilaminar disk-like structure, amnion-, and yolk sac-like compartment with the inner cavity. 3D rendering of the thresholded immunofluorescence signal. Acquisition with Z7 light-sheet microscope and processing with Imaris v10.0.1.

Supplementary Video 3. 3D reconstruction of the day 6 human SEM (WIBR1 cell line).
Immunofluorescence for epiblast- (OCT4, cyan), yolk sac- (SOX17, yellow), trophoblast-like compartment (CK7, magenta), and nuclei (DAPI, white). 0 – 10 sec, 3D view of the outer trophoblast-like layer. 10 – 50 sec, 3D segmentation of the epiblast- (cyan) and hypoblast-like cells (yellow) with DAPI maximum projection. 21 sec – 41 sec, slicing through the 3D volume showing the inner structure of the SEM comprised of bilaminar disk-like structure with early amnion-like compartment, connected to the outer trophoblast-like layer. Immunofluorescence signal and tissue segmentation are outlined. Acquisition with Z7 light-sheet microscope and processing with Imaris v10.0.1.

Supplementary Video 4. 3D reconstruction of the human SEM at day 6 demonstrating pro-amniotic-like cavity formation within the epiblast-like compartment.
3D reconstruction of the human SEM at day 6 shows its 3D morphology (0 – 12 sec) and beginning of the proamniotic-like cavity formation within the epiblast-like compartment (12 – 18 sec). Immunofluorescence for OCT4 (epiblast, cyan), F-ACTIN (red), and nuclei (DAPI, white). The image was acquired with Zeiss LSM 800 microscope and processed with Imaris v10.0.0.

Supplementary Video 5. 3D reconstruction of the human SEM at day 8 shows formation of embryonic disk-like structure and amnion-like compartment.

3D reconstruction of the embryonic disk-like structure (SOX2, cyan) and amnion-like compartment (TFAP2A, magenta) in the day 8 human SEM. 3D segmentation of epiblast-like tissue (cyan) and amnion-like tissue (pink) is denoted as the semi-transparent outline together with the corresponding immunofluorescence signal. 17 – 21 sec, human SEM epiblast-like compartment has a disk shape. The image was acquired with Z7 light-sheet microscope and processed with Imaris v10.0.0.

Supplementary Video 6. 3D reconstruction of the human SEM at day 6 shows yolk sac-like morphology and polarity.

3D reconstruction of human day 6 SEM showing yolk sac-like structure (marked by SOX17, yellow). 5 – 18 sec, zoom into the visceral and parietal yolk sac-like cells having columnar and squamous cell shape, respectively. The cells exhibit apical polarization (aPKC, green). F-ACTIN (red), nuclei (DAPI, white). The image was acquired with Zeiss LSM 700 microscope and processed with Imaris v10.0.0.

Supplementary Video 7. 3D reconstruction of the human SEM at day 8 shows ExEM-like cell integration underneath the yolk sac-like structure.

3D reconstruction of the human SEM at day 8 showing ExEM-like cells marked by expression of VIM (red) and located predominantly underneath the yolk sac-like structure (yellow); OCT4 (epiblast, cyan), nuclei (DAPI, grey). 3D rendering of the thresholded immunofluorescence signal. The image was acquired with Zeiss LSM 700 microscope and processed with Imaris v10.0.0.

Supplementary Video 8. 3D reconstruction of human SEM at day 6 shows development of the syncytiaotrophoblast-like layer with lacunae-like structures.

3D reconstruction of the human SEM at day 6 showing development of the syncytiotrophoblast-like layer, expressing both SDC1 (magenta) and HCGB (green). The syncytiotrophoblast-like compartment forms multiple lacunae-like structures. F-ACTIN (red), nuclei (DAPI, grey). 11 – 19 sec, slicing through the 3D volume showing the inner structure of the multiple trophoblast lacunae-like structures. The image was acquired with Zeiss LSM 700 microscope and processed with Imaris v10.0.0.

Supplementary Figure 1

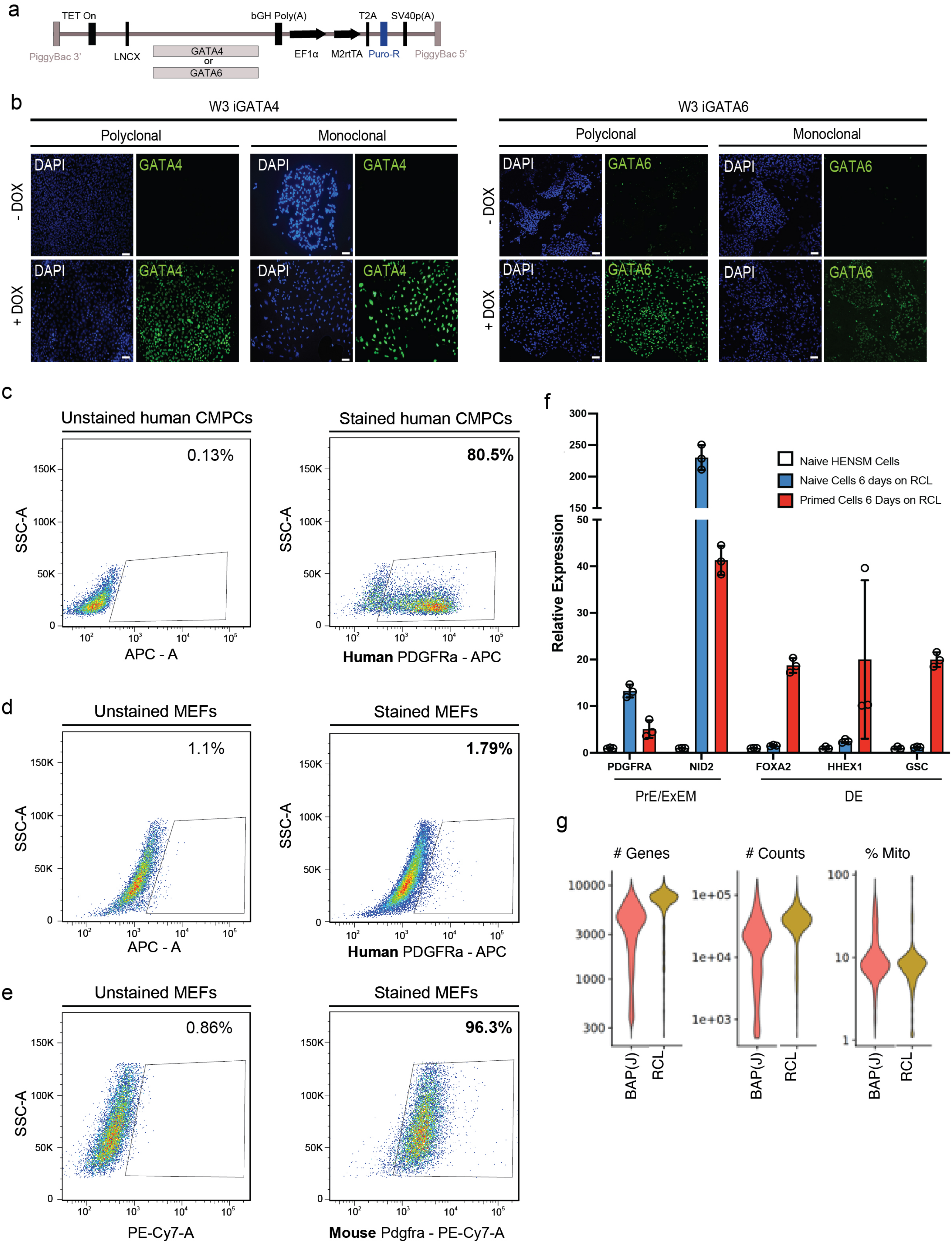

Supplementary Figure 1. Cell lines and strategies to obtain and define PrE/ExEM-like cells from human naïve ESCs.

a, scheme of the donor plasmid vector used for genomic integration of the DOX-inducible iGATA4 or iGATA6 overexpression in human ESCs. **b**, representative immunofluorescence images of mono- and polyclonal iGATA4 (left) and iGATA6 (right) WIBR3 hESC (W3) clones, showing uniform expression of GATA4 (green) and GATA6 (green) in response to DOX; nuclei (DAPI, blue). Scale bars, 50 µm. **c**, control FACS plots of human cardiomyocyte precursor cells (CMPCs, known to express high levels of PDGFRa) unstained **(left)** or stained with anti-human PDGFR **(right)**. **d**, FACS plots of unstained **(left)** and anti-human PDGFRa-stained **(right)** mouse embryonic fibroblasts (MEFs), validating specificity of the anti-human PDGFRa antibody. **e**, FACS plots of MEFs either unstained **(left)** or stained with anti-mouse PDGFRa antibody **(right)**. The staining pattern validates high species-specificity of the used antibodies and the none-to-negligible contribution of MEFs to the PDGFRa+ fraction when confluent human cells, following MEF depletion, were stained with the human-specific anti-PDGFRa antibody. **f**, Representative qRT-PCR gene expression (normalized by GAPDH and ACTIN) of the endodermal markers for PrE/ExEM-like cell induction in RCL medium starting form naïve (blue) vs primed (red) human ESC. Naïve human ESCs maintained in HENSM (white) were used as a reference control. DE – definitive endoderm. Values of each sample represent average value of 3 technical replicates per sample, error bars indicate s.d.. A single representative experiment out of N=3 biological replicates performed is shown. **g (left to right)**, numbers of genes, total read counts, and percentage of mitochondrial read counts in the scRNA-seq samples of HENSM ESC derived cells induced in BAP(J) or RCL conditions for 3 days.

Supplementary Figure 2

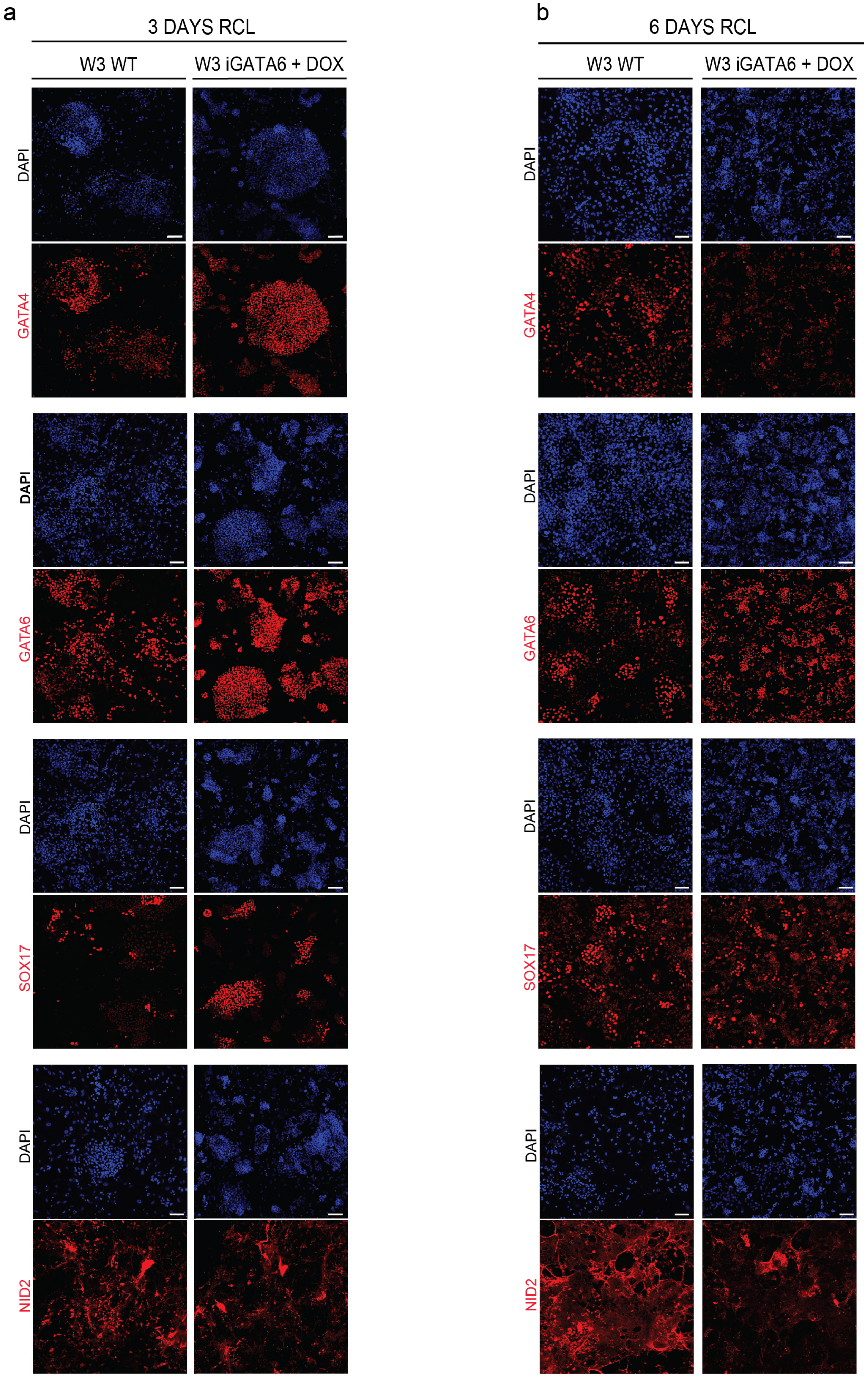

Supplementary Figure 2. Immunostaining analysis of RCL induced cells.

a, b, representative immunofluorescence images of WIBR3 WT and WIBR3 iGATA6 naïve ESCs with DOX induced in RCL media for 3 or 6 days (**a** and **b**, respectively), showing expression of the indicated PrE markers (red), including SOX17, and nuclei (DAPI, blue)**.** Scale bars, 100 µm.

Supplementary Figure 3

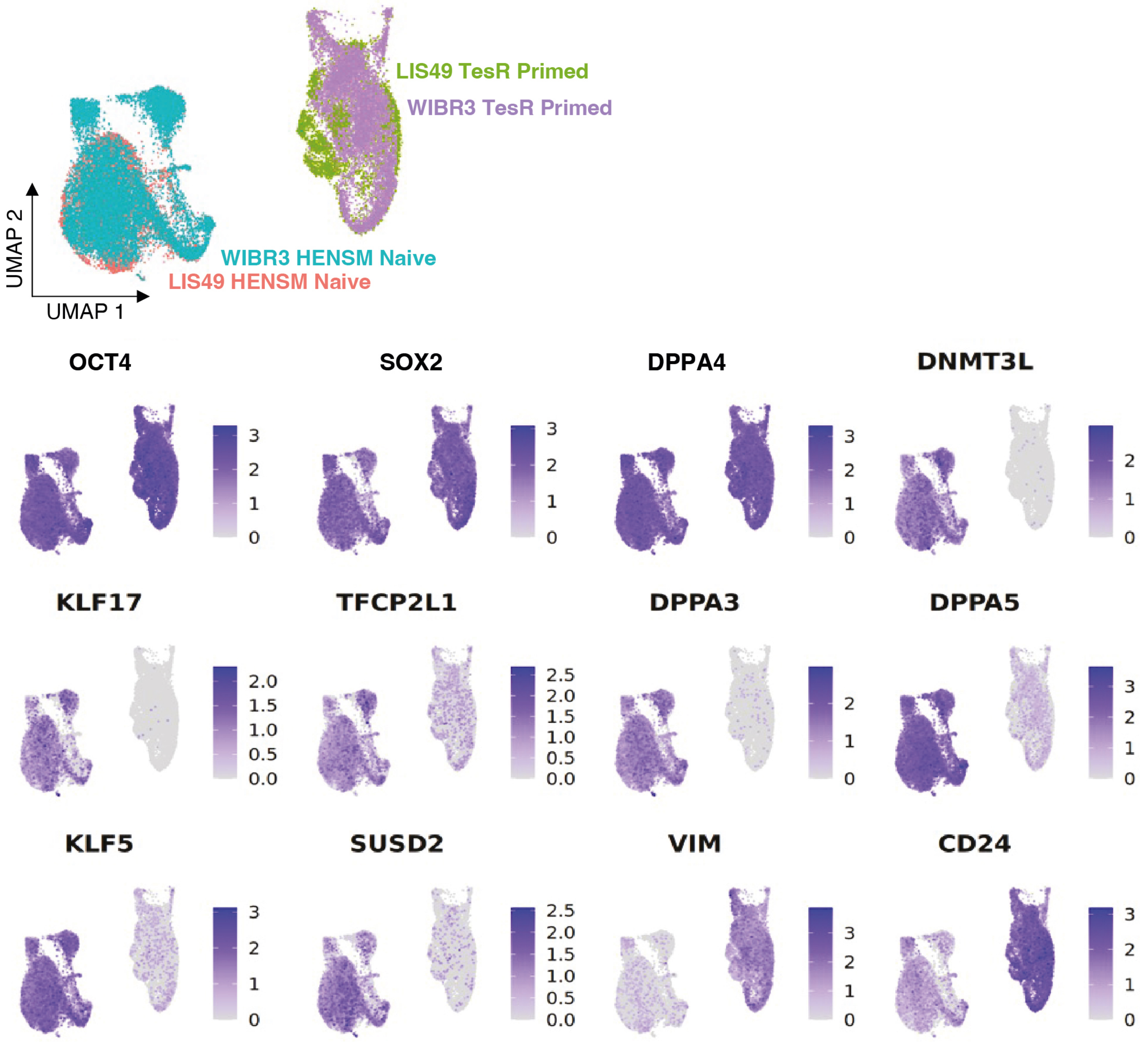

Supplementary Figure 3. scRNA-seq analysis of human naïve ESCs grown in HENSM vs. isogenic primed ESCs. scRNA-seq analysis of isogenic primed and early passage (P3) HENSM naïve human ESC lines (LIS49 naïve n=9517 cells, WIBR3 naïve n=9798 cells, LIS49 primed n=7916 cells, WIBR3 primed n=6287 cells). UMAPs demonstrate expression of naïve markers (e.g., DNMT3L, KLF17, DPPA3, DPPA5, KLF5) specifically in HENSM naïve cells, and a higher expression of primed pluripotency markers (CD24 and Vimentin) in primed cells rather than in HENSM naïve cells.

Supplementary Figure 4

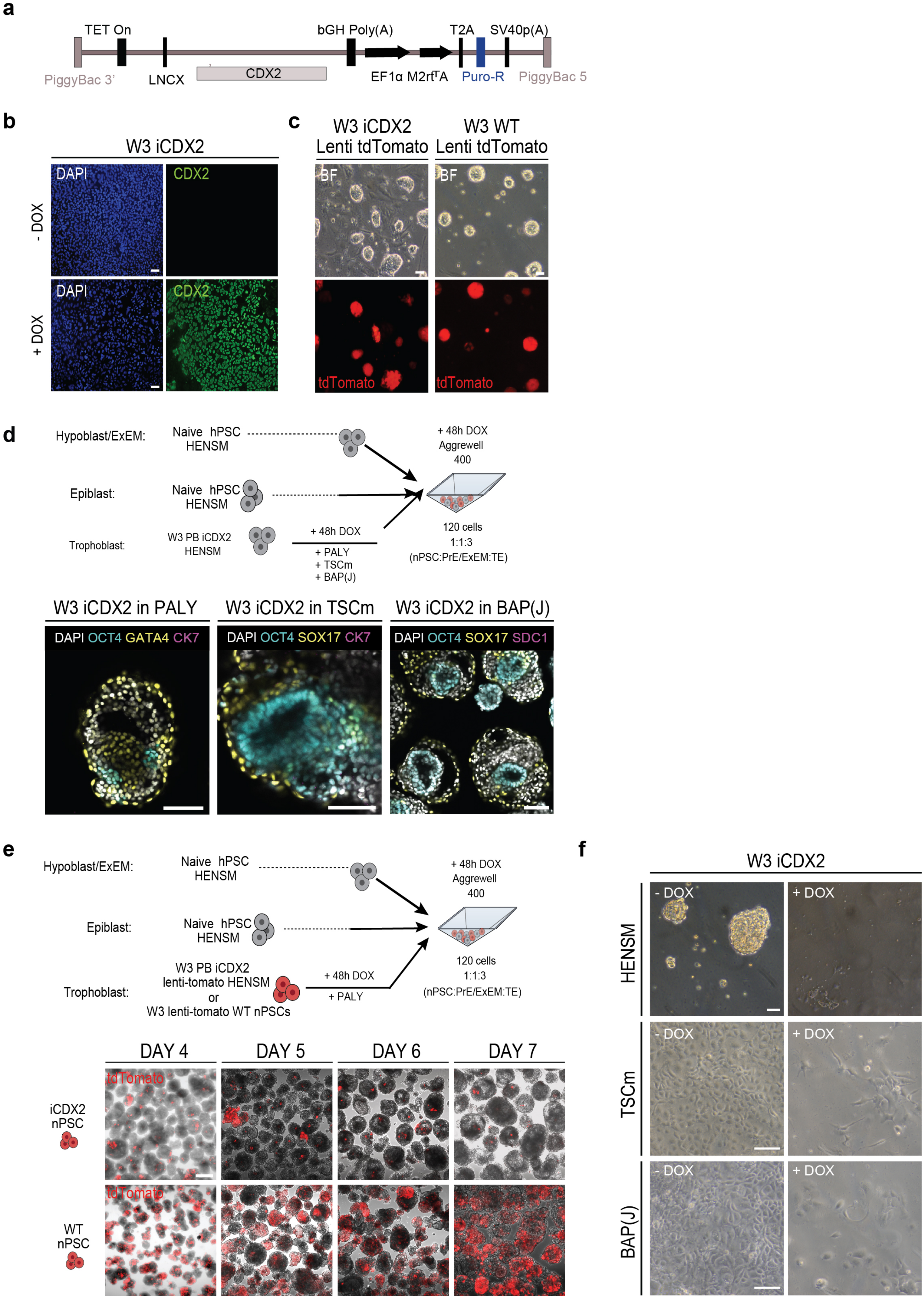

Supplementary Figure 4. Characterizing the capacity of CDX2 overexpression to generate TE/TSC-like cells from human naïve ESCs compatible with successful SEM formation.

a, scheme of the donor plasmid vector used for genomic integration of the DOX-inducible CDX2 overexpression in human ESCs. **b**, representative immunofluorescence images of iCDX2 cells, showing CDX2 (green) expression only in response to DOX; nuclei (DAPI, blue). Scale bars, 50 µm. **c**, representative images of iCDX2 (left) and WT (right) human W3 hESCs in HENSM conditions, showing live fluorescence of tdTomato (red) after transfection with lentiviral particles carrying the fluorophore. Scale bars, 50 µm. **d (top)**, the scheme of the experiment, where naïve ESCs and hypoblast/ExEM-like induced cells were aggregated with iCDX2 cells, induced by DOX for 48h in three different media, PALY (N2B27 supplemented with **P**D0325901, **A**83-01, h**L**IF, and **Y**-27632), TSCm[13], and BAP(J) (DMEM/F12 based medium with ALK4/5/7 inhibitor A83-01, FGF2 inhibitor **P**D0325901, and BMP4 substituted with JAK inhibitor I after 24h). **d (bottom)**, representative immunofluorescence images showing no surrounding trophoblast in aggregates with iCDX2 cells, regardless of the media conditions; epiblast (OCT4, cyan), hypoblast (GATA4, SOX17, yellow), trophoblast (CK7, SDC1, magenta), nuclei (DAPI, blue). Scale bars (from left to right), 100 µm, 50 µm, 100 µm. **e (top),** the scheme of the experiment, in which naïve ESCs and PrE/ExEM-like induced cells were aggregated with WT or iCDX2 tdTomato-labelled nESC induced towards trophectoderm (TE) in PALY media with or without DOX as indicated. **e (bottom),** representative brightfield and live fluorescence images of day 4 – 7 aggregates showing localization of the trophoblast. Scale bar, 200 µm. **f**, representative phase contrast microscopy images showing iCDX2 cells induced for 72h in different media (HENSM, BAP(J), TSCm) with or without DOX, showing reduced viability upon iCDX2 transgene overexpression. In all conditions, 750,000 iCDX2 cells were seeded in 10 cm Matrigel coated plates, and DOX induction was started 24h after seeding. Scale bars, 100 µm.

Supplementary Figure 5

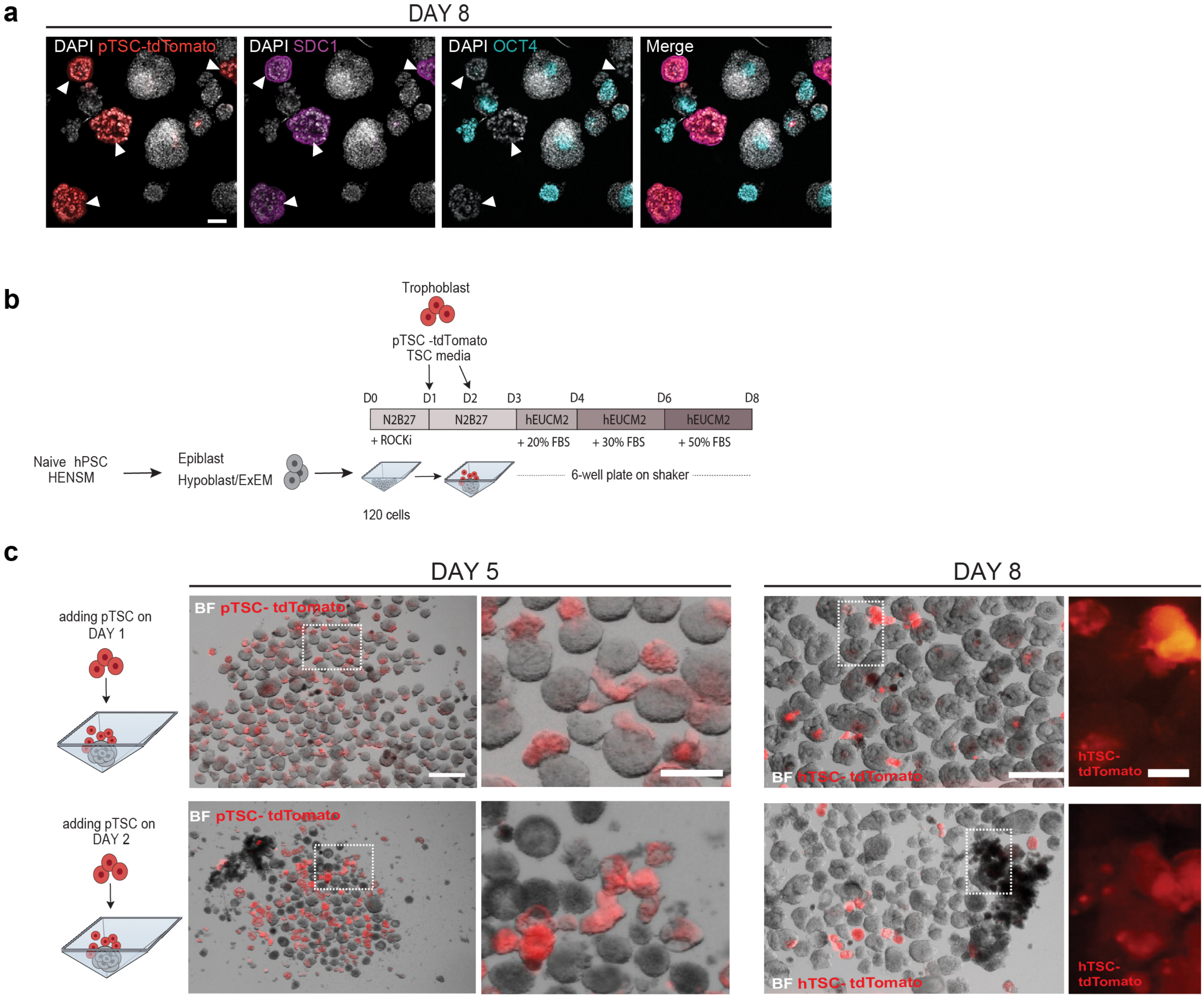

Supplementary Figure 5. Human TSC lines do not sort adequately in human SEM protocol.

a, representative immunofluorescence images of day 8 aggregates, showing OCT4 (cyan), SDC1 (magenta), and TSCs, labelled by tdTomato; nuclei (DAPI, white). Scale bars, 50 µm. White arrows highlight TSC clumps. **b**, sequential aggregation of nESCs (in HENSM) with tdTomato-expressing primed ESC derived TSC line on the first or second days of the aggregation protocol. **c**, representative brightfield images and live fluorescence of tdTomato (red) in day 5 and 8 aggregates with TSCs, added on the first (top) or second (bottom) days of aggregation; scale bar, 200 µm. Right, zoom into the several SEMs with tdTomato signal. Scale bar, 50 µm. The aggregates with TSCs do not form a uniformly surrounding Tb-like layer, but remain as isolated clumps.

Supplementary Figure 6

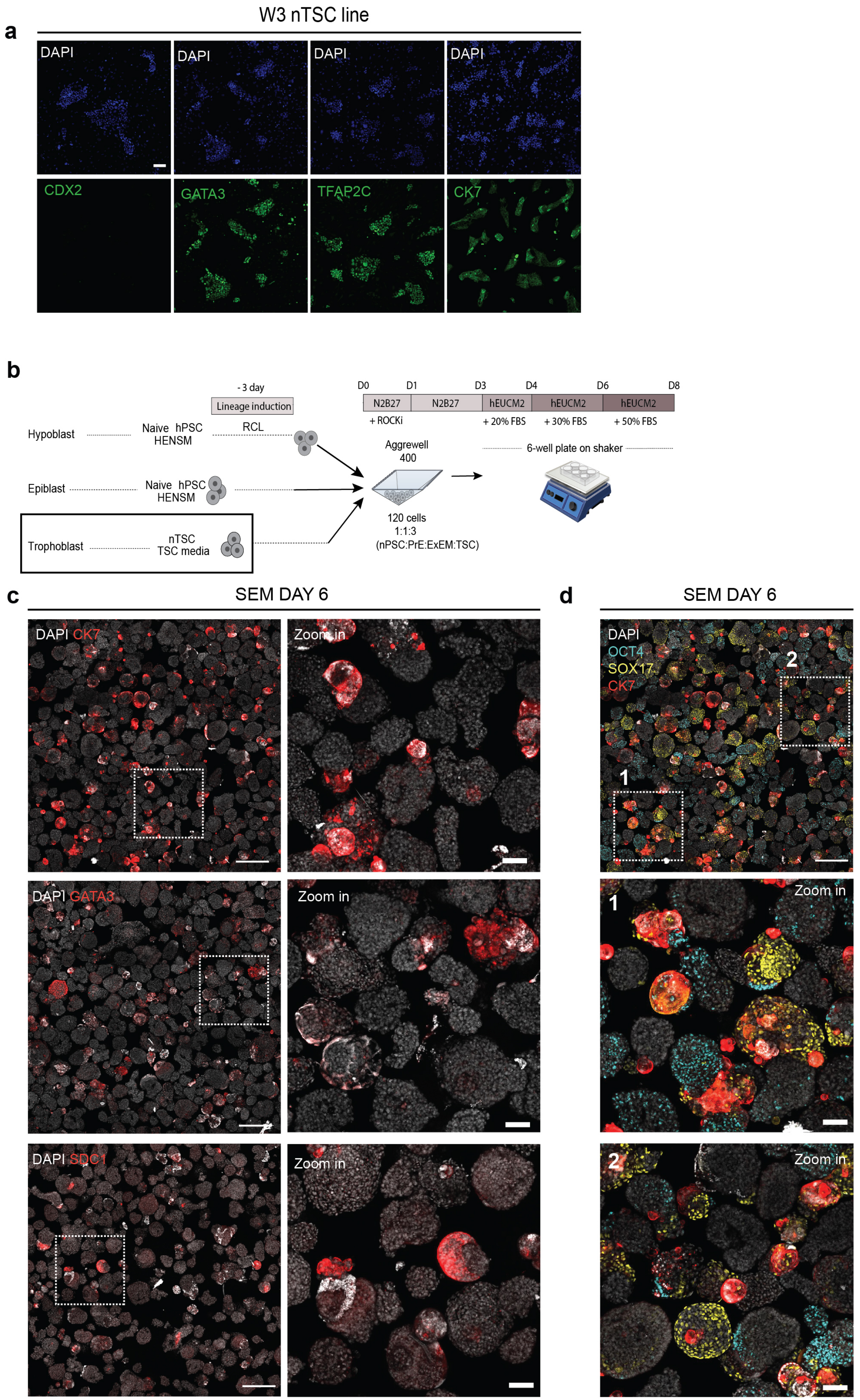

Supplementary Figure 6. Human naïve ESC derived TSC lines do not sort adequately in human SEM protocol.

a, representative immunofluorescence images validating correct expression of TSC marker genes in colonies of a WIBR3 (W3) naïve hESC-derived TSC line (termed nTSC). CDX2, GATA3, TFAP2C, CK7 (all in green); DAPI (blue). Scale bar, 100 µm. **b,** scheme for aggregation protocol of naïve pluripotent stem cells (nESCs) in HENSM media, naïve-derived trophoblast stem cells (nTSCs), and nESCs induced in RCL towards PrE/ExEM-like cells for 3 days. **c (left)**, representative immunofluorescence images showing rare expression of CK7, GATA3, and SDC1 (all in red) trophoblast markers in the aggregates. nuclei (DAPI, white); scale bar, 500 µm. **c (right)**, zoom into several aggregates with CK7 expression; scale bar, 100 µm. **d**, representative immunofluorescence image from (upper left panel in **(c)**, showing Epi-like cells (OCT4, cyan) and PrE-like cells (SOX17, yellow) with CK7 (red); nuclei (DAPI, white). Bottom, zoom images of the outlined regions are shown. Although some aggregates express lineage markers, they do not organize into embryoid-like structures and are not uniformly surrounded by the trophoblast. Scale bar, 500 µm (top); bottom, 100 µm.

Supplementary Figure 7

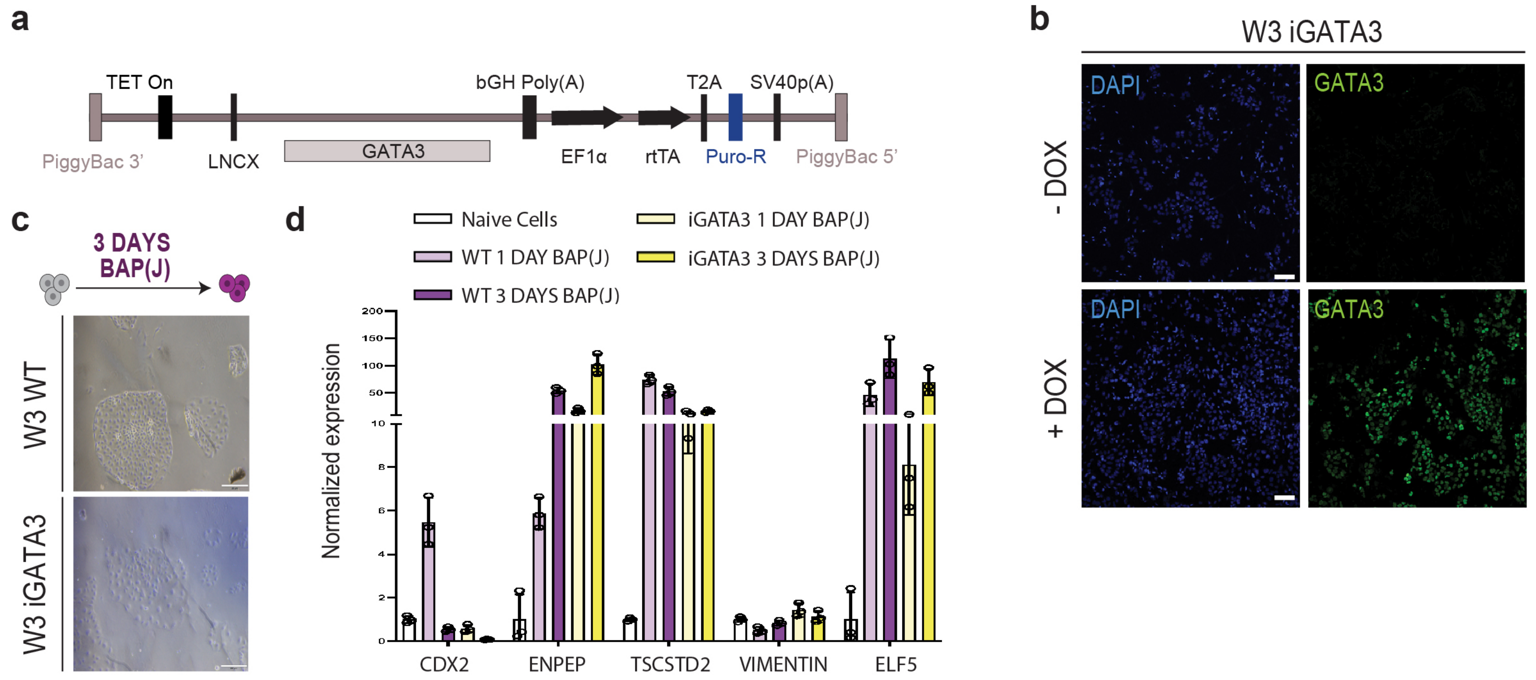

Supplementary Figure 7. Characterizing the capacity of GATA3 overexpression to generate TE/TSC-like cells from human naïve ESCs compatible with successful SEM formation.

a, scheme of the donor plasmid vector used for genomic integration of the DOX-inducible iGATA3 overexpression transgene. **b**, representative immunofluorescence images of iGATA3 cells, showing uniform GATA3 expression (green) in response to DOX; nuclei (DAPI, blue). Scale bars, 100 µm. **c**, brightfield images of WT (top) and iGATA3 (bottom) cells after incubation in BAP(J) media for three days. Scale bars, 200 µm. **d**, Representative qRT-PCR gene expression (normalized by GAPDH and ACTIN) of the trophoblast markers for WT naïve pluripotent stem cells (nESCs) in BAP(J) media (purple) and iGATA3 nESC cells induced by DOX in BAP(J) media (yellow), versus nESCs maintained in HENSM media and used as a reference control (set as 1) (white). Values of each sample represent average value of 3 technical replicates per sample, error bars indicate s.d. A single representative experiment out of N=3 biological replicates performed is shown.

Supplementary Figure 8

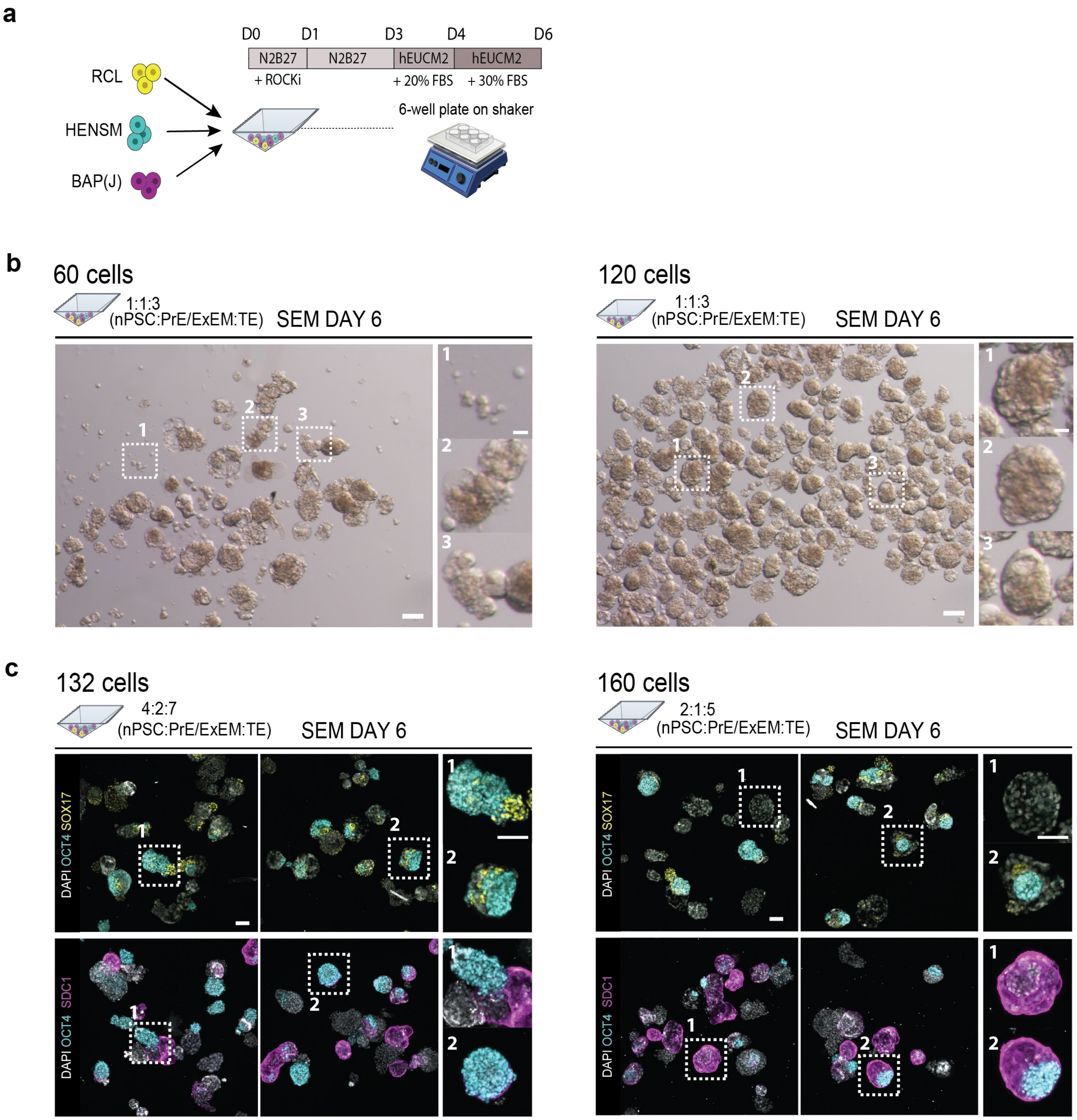

Supplementary Figure 8. Optimization of the cell numbers for co-aggregating conducive human SEMs exclusively from human naïve ESCs. a, scheme of the protocol: induced PrE/ExEM-like cells (yellow), WT naïve pluripotent stem cells (nESCs) maintained in HENSM medium (cyan), and WT nESC induced towards trophoblast-like cells with BAP(J) medium (magenta) were aggregated in Aggrewell 400 at different ratios in N2B27 and grown as indicated in the scheme until day 6. **b (left)**, representative brightfield images of day 6 SEMs, aggregated from a total 60 cells at 1:1:3 (nESC: PrE/ExEM-like: TE-like) cell ratio, showing mostly small and fragmented aggregates. **b (right)**, brightfield images of day 6 SEMs, aggregated from total 120 cells at 1:1:3 (nESC: PrE/ExEM-like: TE-like) cell ratio, showing lower fragmentation tendency and more frequent formation of bigger aggregates. This condition was chosen for SEM generation. Scale bar, 500 µm; zoom, 125 µm. **c**, representative immunofluorescence images of day 6 SEMs showing epiblast (OCT4, cyan), hypoblast (SOX17, yellow), and trophoblast (SDC1, magenta); nuclei (DAPI, white). Day 6 SEMs aggregated from total 132 cells at 4:2:7 (nESC: PrE/ExEM-like: TE-like) cell ratio **(left)**, or from total 160 cells at 2:1:5 (nESC: PrE/ExEM: TE) cell ratio **(right)** showing inadequate organization of SEMs, when compared to optimized conditions (120 cells at 1:1:3 nESC: PrE/ExEM-like: TE-like). Scale bars, 100 µm.

Supplementary Figure 9

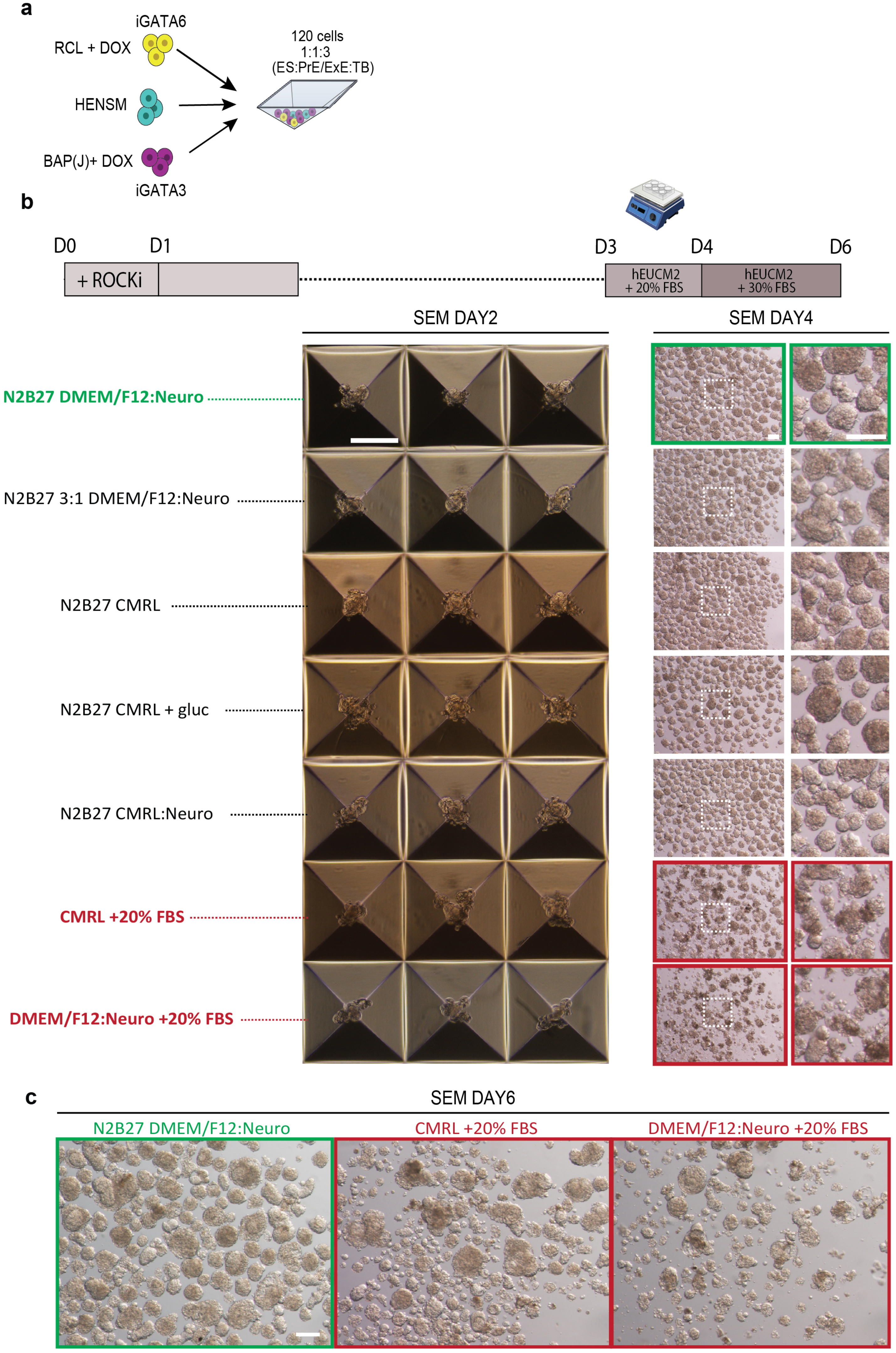

Supplementary Figure 9. Optimization of the aggregation conditions for human SEM generation from naïve ESCs. a, scheme of the protocol: PrE/ExEM-like primed/induced cells in RCL medium (yellow), WT naive pluripotent stem cells (nESCs) maintained in HENSM medium (cyan), and iGATA3 TE-like cells induced with DOX in BAP(J) medium (magenta) were aggregated in different media in Aggrewell 400 plate at 1:1:3 (nESC: PrE/ExEM-like: TE-like) ratio and cultured until day 6. **b (bottom to top)**, representative brightfield images of day 2 and day 4 SEMs, aggregated in DMEM-F12:Neurobasal (1:1) supplemented with 20% FBS or N2B27, CMRL 1066 base medium supplemented with 20% FBS or N2B27, CMRL supplemented with N2B27 and extra-added 3 mg/ml D-Glucose, and DMEM F12: Neurobasal (3:1). Although addition of FBS is beneficial for SEM growth, FBS impairs aggregation by day 4, and the best aggregation efficiency was consistently observed in DMEM-F12:Neurobasal supplemented with N2B27 without FBS. Scale bars, 200 µm. c, representative brightfield images showing SEM morphology at day 6 after aggregation in DMEM-F12:Neurobasal and CMRL supplemented with 20% FBS. Addition of FBS impaired formation of the aggregates while N2B27 conditions allowed better human ESC derived aggregate growth. Scale bars, 200 µm.

Supplementary Figure 10

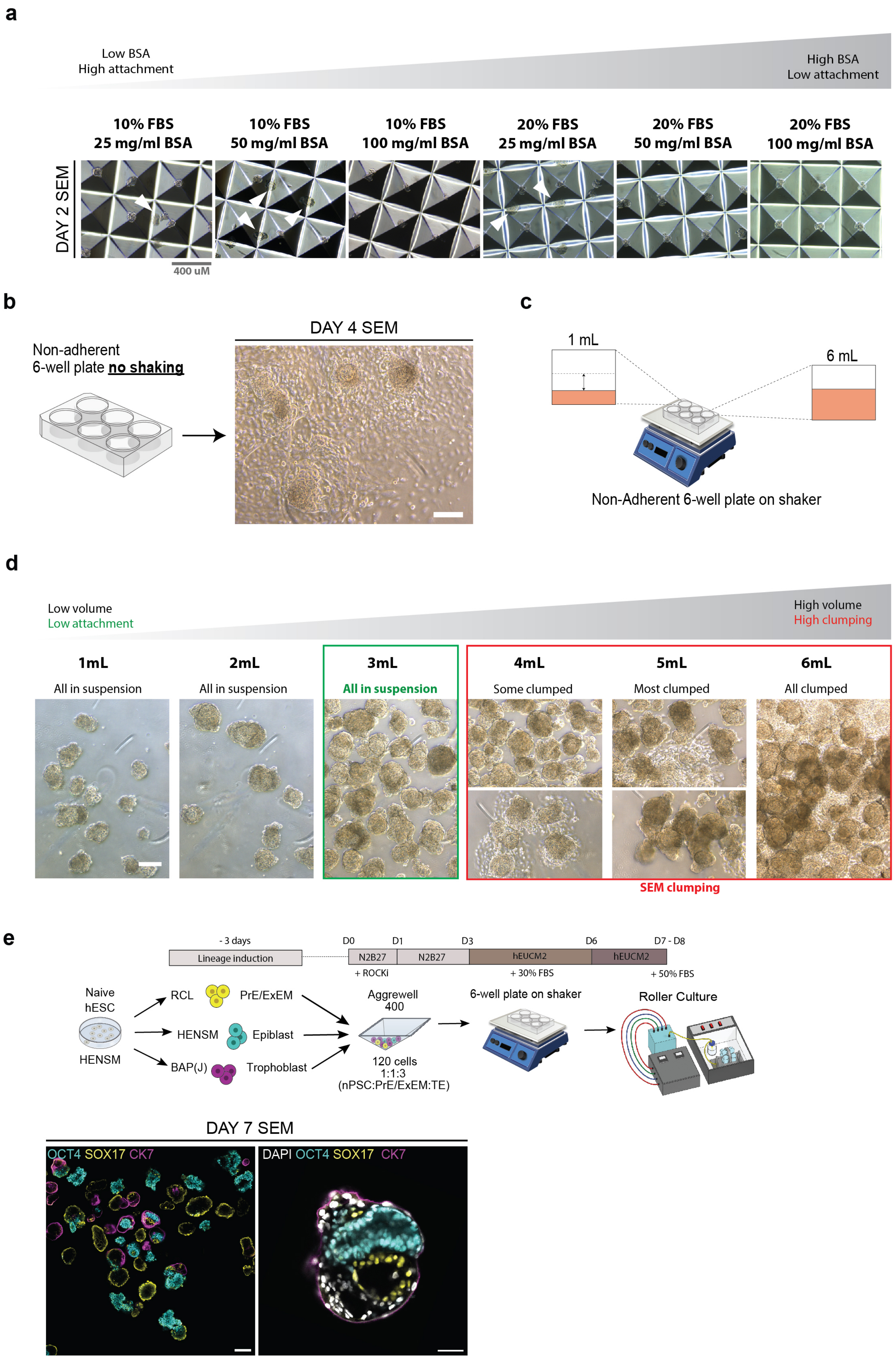

Supplementary Figure 10. Optimization of human SEM culture conditions. a, representative brightfield images of day 2 SEM aggregates inside the Aggrewell 400 plate; addition of BSA to the indicated aggregation medium prevents attachment of the aggregates to the plate edges (white arrows). Scale bar, 400 µm. Optimal BSA concentration chosen for further experimentation is highlighted in green. **b**, growth of SEMs in 6-well non-adherent plates without shaking leads to their attachment to the plate and disruption of morphology. Scale bar, 200 µm. **c**, scheme of the experiment on the non-adherent 6-well dish, using orbital shaking (day 4-8), in which the volume of the culture media was optimized. **d**, representative brightfield images of multiple SEMs showing that the rate of clumping is dependent on the media volume (1 – 6 ml). The optimal suspension condition is outlined in green (3ml). Scale bar, 200 µm. **e (top)**, the scheme of roller culture test, where aggregates were generated as described (see Methods), cultured until day 6 on a shaker, followed by roller culture in hEUCM2 50% FBS 20%O_2 5%CO2. **e (bottom)**, representative immunofluorescence images of day 7 SEMs in this regimen, showing Epi-like (OCT4, cyan), hypoblast-like (SOX17, yellow), and trophoblast-like (CK7, magenta) compartment; nuclei (DAPI, white), brightfield (BF); scale bar, 200 µm. Right, example of the SEM in which epiblast-like, hypoblast-like, and trophoblast-like compartments are adequately compartmentalized; scale bar, 50 µm.

Supplementary Figure 11

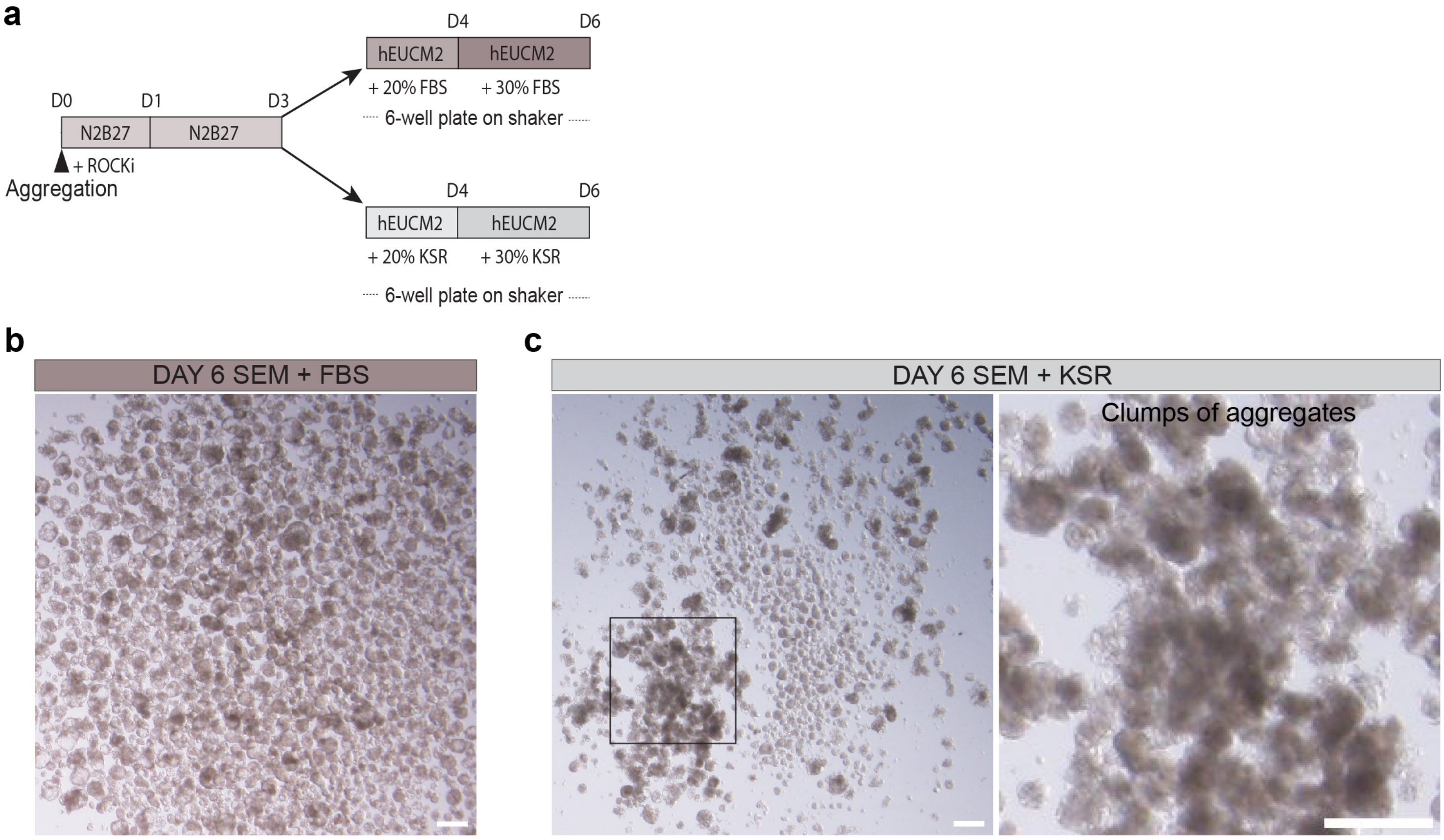

Supplementary Figure 11. KSR does not substitute FBS in hEUCM2 conditions.

a, scheme of culture protocol is shown, where aggregates were generated as described (see Methods) and cultured on a shaker until day 6 in hEUCM2 supplemented with FBS (top) or in hEUCM2 supplemented with KSR (bottom). **b,** representative brightfield image of multiple SEMs cultured in the hEUCM2+FBS condition showing no clumping. Scale bar, 500 µm. **c,** representative brightfield images of multiple SEMs cultured in the hEUCM2+KSR condition, resulting in formation of large clumps from multiple aggregates that prohibit their structure and organized further development (bottom). Scale bars, 500 µm.

Supplementary Figure 12

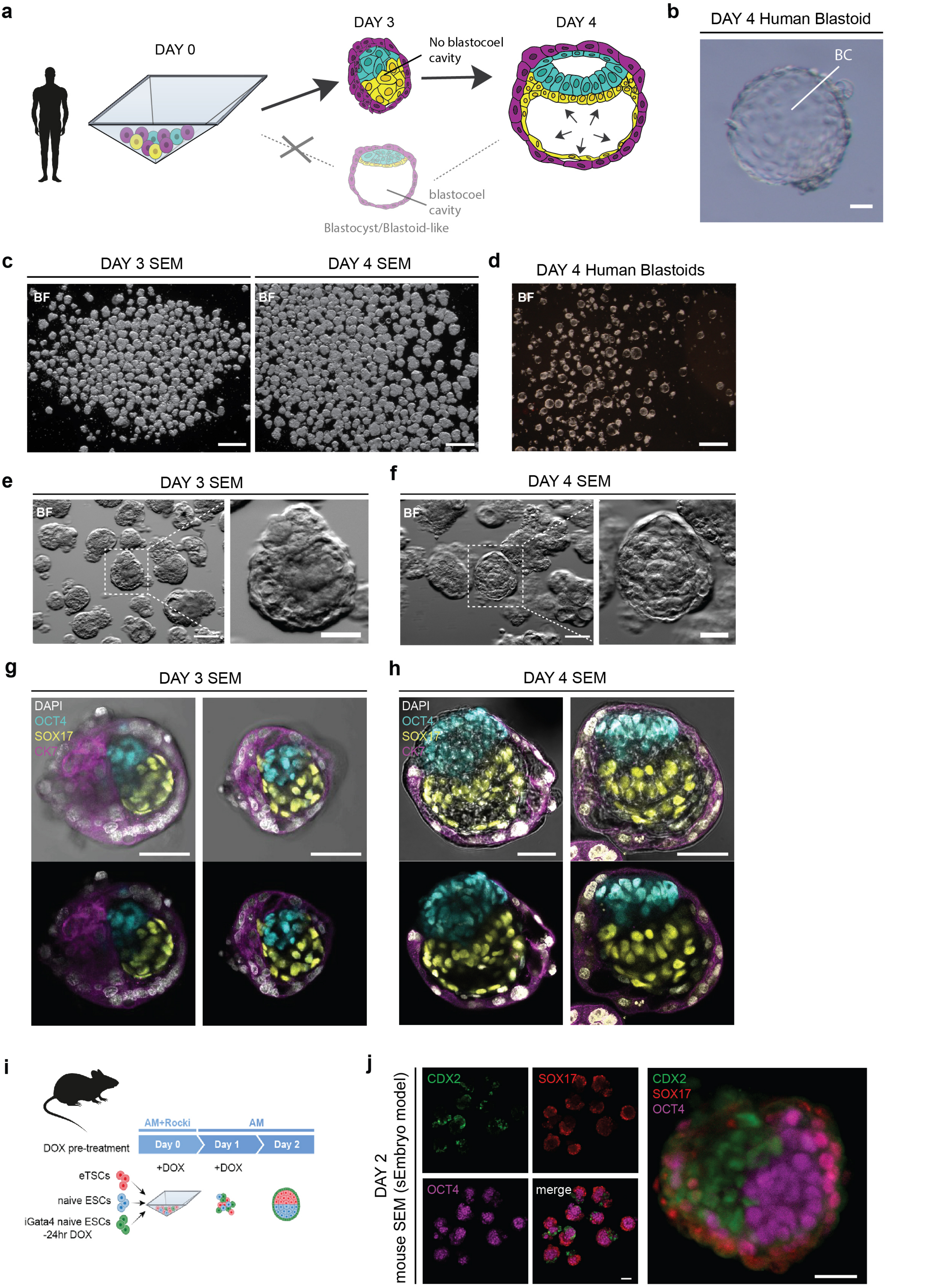

Supplementary Figure 12. Human and mouse SEMs bypass blastocyst-like stage. a, scheme showing the distinction between human SEM (top) and human blastoid (bottom) aggregation protocols: as opposed to blastoids, SEMs do not form a blastocoel cavity. Epiblast (cyan), hypoblast (yellow), trophoblast (magenta). **b**, representative brightfield image of a blastoid with blastocoel cavity (BC). Scale bar, 100 µm. **c**, representative brightfield images of day 3 and day 4 SEMs showing no blastocoel-like cavity. Scale bar, 200 µm. **d**, representative brightfield images of day 4 human blastoids with blastocoel. Scale bar, 200 µm. **e-f**, enlarged brightfield (BF) images of the SEMs showing no blastocoel-like cavity at day 3 or day 4 human SEMs **(f).** Scale bar, 100 µm; zoom in, 50 µm. **g-h**, representative brightfield and immunofluorescence images of day 3 **(g)** and day 4 **(h)** human SEMs showing developmental progression of epiblast-like (OCT4, cyan), hypoblast-like (SOX17, yellow), and trophoblast-like (CK7, magenta) compartments. Nuclei (DAPI, white). Scale bars, 50 µm. **i**, scheme showing mouse SEM (also known as sEmbryo or SWEM) aggregation with murine stem cells as previously described [3]. **j**, immunofluorescence images showing epiblast (OCT4, magenta), hypoblast (SOX17, red), and trophectoderm (CDX2, green). Like human SEMs, mouse SEMs (sEmbryo models) do not form a BC. Scale bars, 50 µm; zoom in, 20 µm.

Supplementary Figure 13

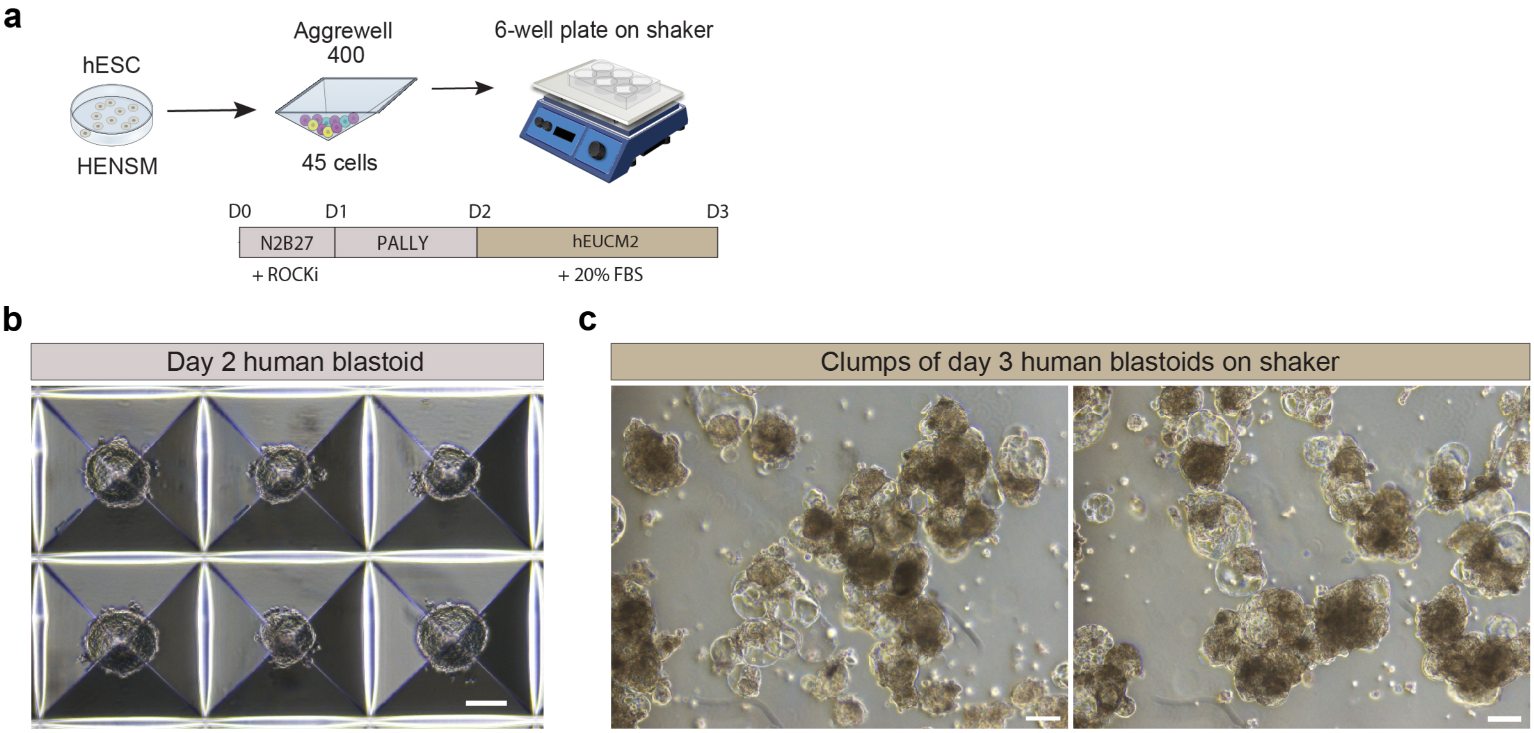

Supplementary Figure 13. Post-implantation development of human blastoids is not supported in SEM protocol described herein. a, scheme of the experiment where naïve WIBR3 hESC in HENSM were aggregated according to (Kagawa et al., 2022)[15] until day 2 when the blastoids were transferred to the EUCM2 media with 20% FBS in a 6 well plate on a shaker. **b**, representative brightfield image of day 2 blastoids in AggreWell. Scale bar, 100 µm. **c**, representative brightfield images of day 3 blastoids which uniformly form clumps when cultured in day 3 SEM conditions onwards. The experiment was terminated at day 3 because of the mis-organization of transferred blastoids. Scale bar, 100 µm.

Supplementary Figure 14

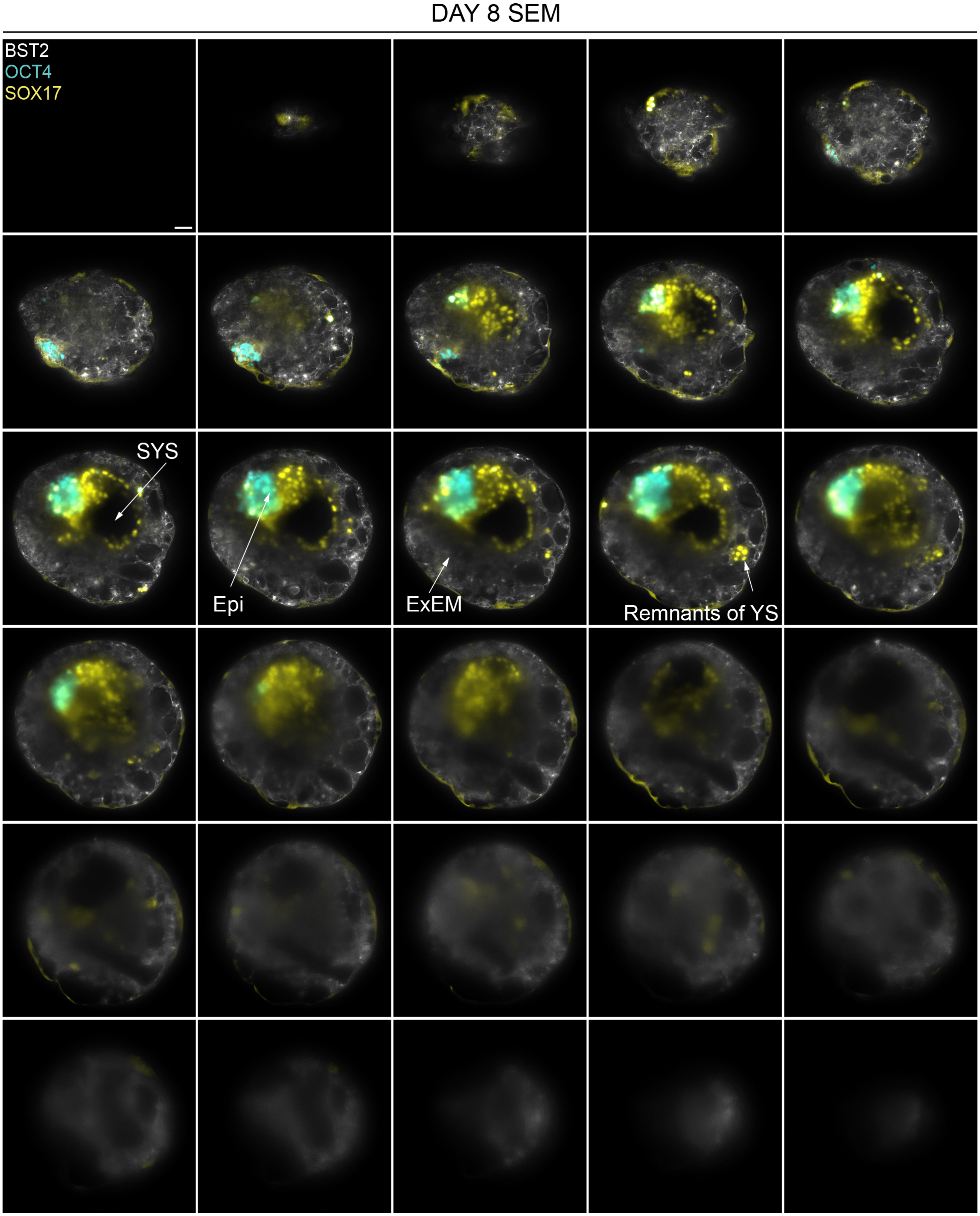

Supplementary Figure 14. 3D structure highlighting the ExEM-like cells and compartment in human SEM at day 8. Individual Z-planes of the 3D immunofluorescence image of day 8 human SEM showing OCT4 (cyan), SOX17 (yellow), and BST2 (white). Epi, epiblast-like; YS, yolk sac-like; ExEM, extraembryonic mesoderm-like; SYS, secondary yolk sac-like; Z-step, 20 µm; scale bar, 50 µm.

Supplementary Figure 15

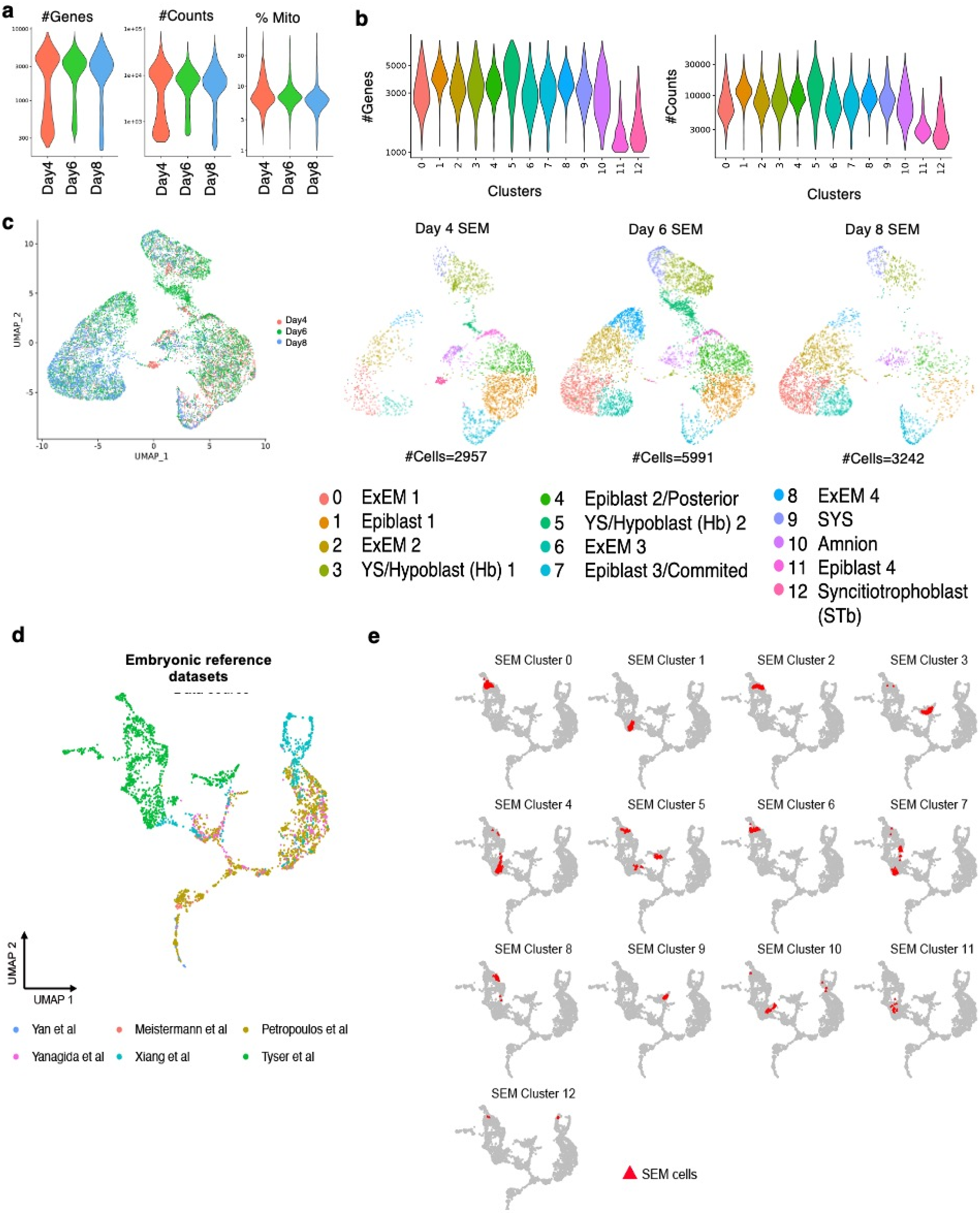

Supplementary Figure 15. Human SEM UMAP quality assessment of scRNA-seq experiment.

a, violin plots indicating the number of genes, the unique molecular identifiers (UMIs), and the percentage of mitochondrial reads obtained per sample prior to filtering. **b,** violin plots indicating the number of genes and the unique molecular identifiers (UMIs) obtained per cluster, after filtering cells with less than 1000 identified genes. **c**, UMAP plot displaying individual cells of the different SEM samples as indicated. Left, colors indicate different samples. Right, colors mark 13 identified clusters as shown in **Fig. 6a**. The number of single cells in each sample is indicated. **d**, UMAP projection of the assembled human embryonic reference dataset with colors indicating data source (related to **Fig. 6e**). **e**, projection of the SEMs cells on the embryonic reference UMAP space stratified by the corresponding raw Seurat clusters. Grey data points represent embryonic reference cells or unselected neighborhood nodes, while each red triangle corresponds to the projection of the representative neighborhood nodes from each SEM raw cluster into the UMAP space (related to **Fig. 6e**).

Supplementary Figure 16

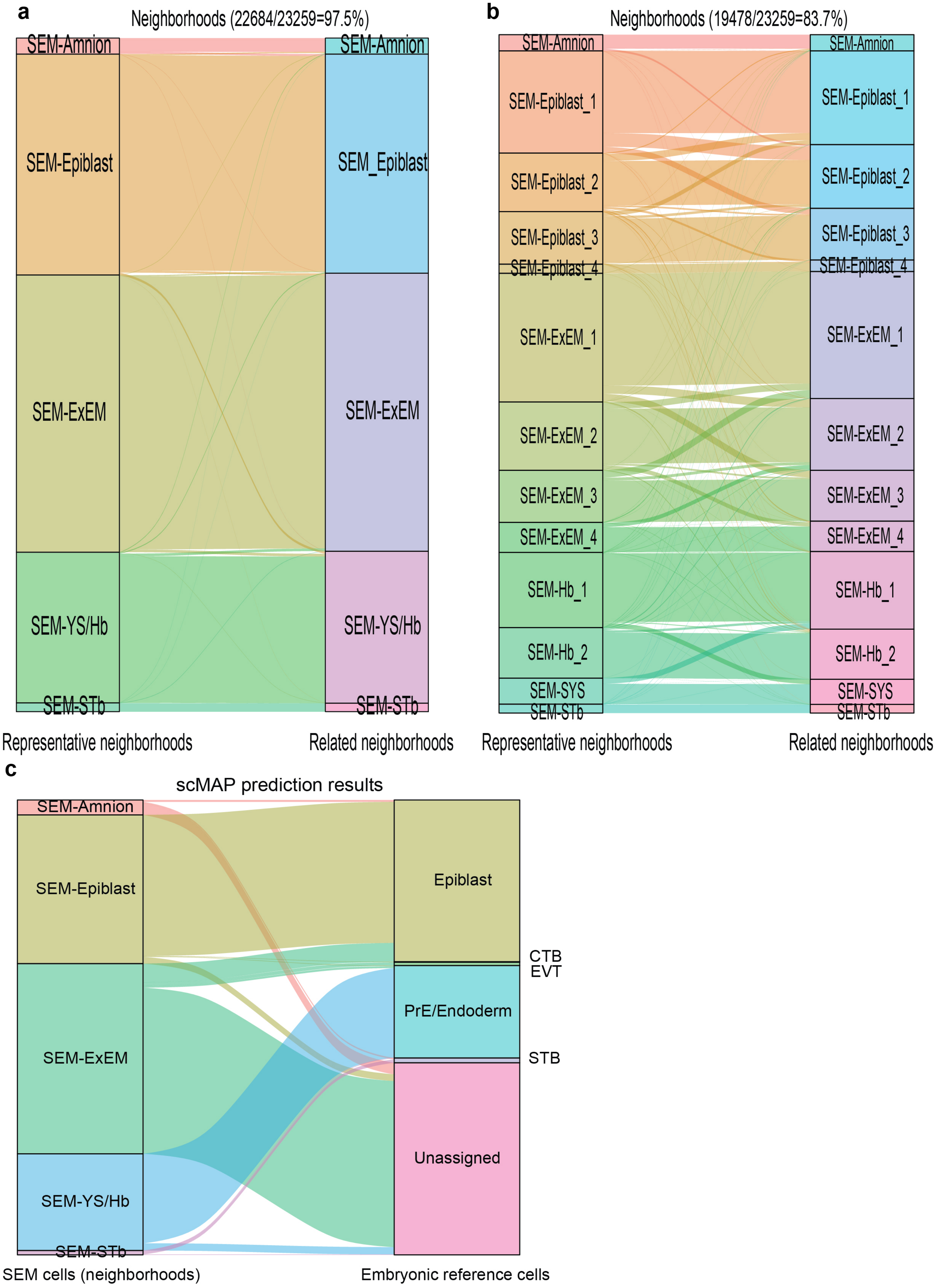

Supplementary Figure 16. SEM scRNA-seq projection on human early embryo reference map.

a – b, alluvial plots comparing the representative node SEM cells to related neighborhood SEM cells based on cell-type (**a**) and Seurat (**b**) annotation. We have checked the assigned cluster information for representative neighborhoods and related neighborhoods. 97.5% and 83.7% of the related neighborhoods have the same cell identity with representative neighborhoods based on lineage information and Seurat cluster information, respectively. Based on this, the neighborhoods are highly homogenous in their cell-type composition. **c**, alluvial plot comparing the cell-type annotations of representative node SEM cells to the predicted identities obtained from scMAP previously described in[31,49]. The prediction results for SEM cells (after aggregation using neighborhood methods) were shown here. The majority of SEM-Epiblast-like, SEM-STb-like and SEM-YS/Hb-like cells were identified. However, the SEM-Amnion-like and SEM-ExEM-like cells were determined as unassigned which is likely due to the low number of embryonic reference cells for these cell types and the relatively low sequencing depth in Tyser et al.[32]

Supplementary Figure 17

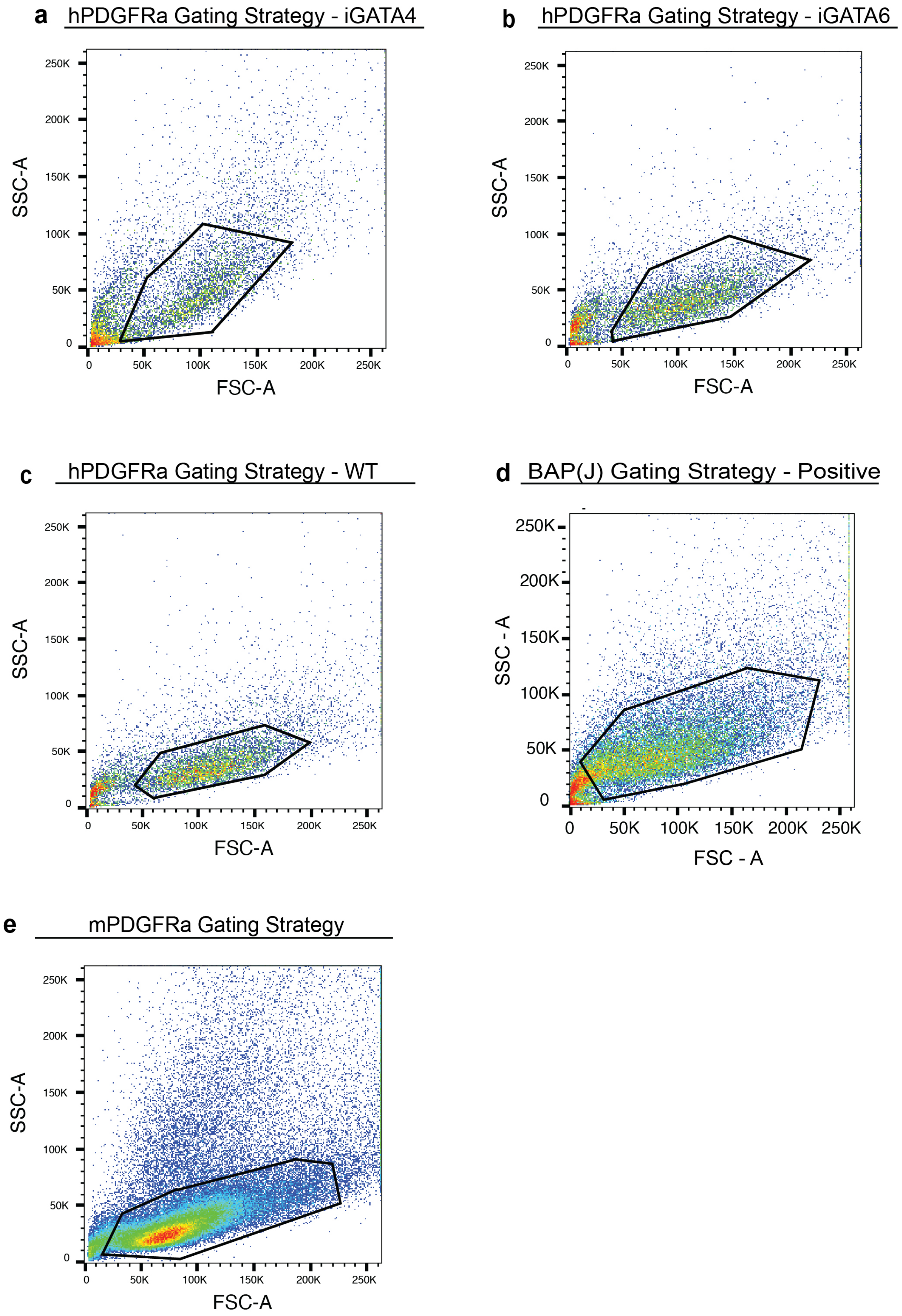

Supplementary Figure 17. FACS analysis gating strategies.

a – e, FACS gating strategies for FSC vs. SSC used in the manuscript as indicated per experimental cell sample.

Supplementary Introduction

Our understanding of human early post-implantation development has been limited due to difficulties in obtaining relevant embryo samples from these early stages of human gestation, and technical and ethical challenges for *in vitro* development of donated blastocysts towards the post-implantation stages while preserving the *in vivo* complexity and correct spatial morphogenesis of both embryonic and extra embryonic compartments. Most of our knowledge on critical early post-implantation stages of human embryo development has been gained solely from histological and anatomical descriptions in embryological collections, and the experimental models for mechanistic research are lacking. Despite the important recent advances[18] in culturing the *in vitro* attached human embryos beyond implantation, these culture systems are restricted in sample number and, overall, do not support normal development of embryos beyond the initiation of early epiblast lumenogenesis [15].

The ability to capture human pluripotent stem cells (ESCs) in different developmental states in culture[57,58] has opened the possibility of investigating early human embryogenesis using 3D stem-cell derived embryo models. By controlling cellular composition and differentiation conditions, the recently introduced elegant models such as gastruloids, blastoids, axioloids, or amniotic sac embryoids[59–70] recapitulate some aspects of early mammalian development in a modular manner. Among them, Blastocyst-like structures (blastoids) are the only currently available human integrated (i.e., comprised of all embryonic and extra-embryonic lineages) model of the human pre-implantation embryo, containing epiblast, primitive endoderm (PrE), and trophectoderm (TE), that can be made with high efficiencies nearing 70%. So far, the attempts to advance the blastoids towards post-implantation stages through *in vitro* culture did not progress beyond what has been achieved with natural human blastocysts[6]. Similarly, mouse blastoids do not develop into bona fide post-implantation embryos even when transferred *in utero*[71]. It is possible that technical improvements in the future might enable further development of blastoids through and beyond gastrulation.

Supplementary Discussion

In this study, we found conditions that allowed naïve (HENSM) human ESCs to form the three extra-embryonic lineages and embryonic epiblast that together are required to assemble a post implantation embryonic structure without transgene induction. These cells assembled into a complex structure that highly resembles the structural and sequential patterning of all embryonic and extraembryonic lineages of the post-implantation human embryo up to initiation of gastrulation. These structures are integrated complete SEMs that mimic human embryo development *ex utero* up to an equivalent of 14 dpc. The human post-implantation integrated SEM generation protocol devised herein reveals the remarkable self-organizing ability of naïve ESCs and provide a window into hereto near impossible to study period of human development.

The fact that human (and mouse) SEM formation bypasses the blastocyst-like stage and that the *ex vivo* protocols for growing natural human blastocysts into the authentic 14 dpf stage are still lacking, makes donated fetal materials from 7-14 dpf the only relevant control for benchmarking the SEM describe herein. Obviously, obtaining new 7-14 dpf human embryo samples is not a viable option for both technical and ethical reasons. However, this limitation is partially mitigated by the available Carnegie and other embryo atlas collections[20,39], as well as the ability to compare human development to NHP embryo models and datasets. However, due to the lack of perfect reference controls, we cannot and do not claim that human SEMs generated herein are identical to natural day 14 human embryos, and it is most likely they are not. We also cannot exclude that some cell types might be missing from the SEMs generated herein or that some of the extra-embryonic-like lineages being primed by protocols devised herein might contain other lineages that are normally not found in the early-post-implantation natural human embryo.

Regarding the variability in developmental stage that can be observed between day 6-8, it could reflect natural variability that could occur during natural development, such as that seen in mouse in utero development where 0.5-1 days variability can be observed. Alternatively, it is possible that this variability results from inherent heterogeneity in the starting induced cell populations made to generate the aggregates or reflect suboptimal protocol development. Developing new conditions that can maintain TE-like state and stable PrE- and ExEM-like cells might contribute to enhancing SEM formation efficiency and reducing variability.

It is possible that the high ectopic expression produced by the PiggyBac system of GATA3 or CDX2, in addition to the endogenous levels induced by the media, leads to unfavorably high levels of such factors which might derail the differentiation outcome and TE/TSC-like properties. Using roller culture after day 6 with the same media composition did not yield a better outcome than orbital shaking placed in static incubators (**Supplementary Fig. 10e**)[3,43]. The latter is consistent with our results in mouse embryos, where the roller culture platform was essential for late gastrulation and organogenesis stages.

Naïve pluripotent stem cell growth conditions typically utilize FGF/MEK signaling inhibition (including HENSM used herein), which leads to the loss of imprinting after extended passaging[72,73], perturbing the developmental potential of such cells and thus might partially underlie the low efficiency yield and SEM quality. This risk may possibly be mitigated in the future by using naïve conditions with titrating down the concentration of FGF/MEK pathway inhibitors or using alternative naïve conditions that do not target this pathway[4,72], which is of future scientific importance to explore and optimize.

It is plausible that upon further experimentation and mechanistic understanding of self-organization, the efficiency and variability in SEM formation can be improved in the future. Devising serum free defined conditions for all different stages of this protocol is also of technical importance. It is also likely that alternative *ex utero* culture platforms, aggregation strategies, or growth conditions will yield similar or enhanced results relative to the ones reported herein with human SEMs. Developing a variety of PSC lines each carrying multiple endogenous fluorescent reporters for different lineage markers is likely to be useful to assess of SEMs with live-cell imaging technologies and add great ease to select those that can be of high-quality for further expansion *ex utero* (rather than relying only on aggregate morphology). Finally, incorporating multi markers staining or FISHs technologies (like HCR - multiplexed, quantitative, high-resolution RNA fluorescence in situ hybridization (RNA-FISH)), might enable evaluating simultaneous presence of multiple compartments and cell types in each SEM.

Supplementary References

56. Lau, K. Y. C. *et al.* Mouse embryo model derived exclusively from embryonic stem cells undergoes neurulation and heart development. *Cell Stem Cell* **29**, 1445-1458.e8 (2022).
57. De Los Angeles, A. *et al.* Hallmarks of pluripotency. *Nature* **525**, 469–478 (2015).
58. Hackett, J. A. & Surani, M. A. Regulatory principles of pluripotency: from the ground state up. *Cell Stem Cell* **15**, 416–430 (2014).
59. Sanaki-Matsumiya, M. *et al.* Periodic formation of epithelial somites from human pluripotent stem cells. *Nat Commun* **13**, (2022).
60. Yamanaka, Y. *et al.* Reconstituting human somitogenesis in vitro. *Nature* **614**, 509–520 (2023).
61. Miao, Y. *et al.* Reconstruction and deconstruction of human somitogenesis in vitro. *Nature* **614**, 500–508 (2023).
62. Matsuda, M. *et al.* Recapitulating the human segmentation clock with pluripotent stem cells. *Nature* **580**, 124–129 (2020).
63. van den Brink, S. C. *et al.* Single-cell and spatial transcriptomics reveal somitogenesis in gastruloids. *Nature* **582**, 405–409 (2020).
64. Van Den Brink, S. C. *et al.* Symmetry breaking, germ layer specification and axial organisation in aggregates of mouse embryonic stem cells. *Development (Cambridge)* **141**, 4231–4242 (2014).
65. Turner, D. A. *et al.* Anteroposterior polarity and elongation in the absence of extraembryonic tissues and of spatially localised signalling in gastruloids: Mammalian embryonic organoids. *Development (Cambridge)* **144**, 3894–3906 (2017).
66. Zheng, Y. *et al.* Controlled modelling of human epiblast and amnion development using stem cells. *Nature* **573**, 421–425 (2019).
67. Shao, Y. *et al.* Self-organized amniogenesis by human pluripotent stem cells in a biomimetic implantation-like niche. *Nat Mater* **16**, 419–427 (2017).
68. Shao, Y. *et al.* A pluripotent stem cell-based model for post-implantation human amniotic sac development. *Nat Commun* **8**, 1–15 (2017).
69. Moris, N. *et al.* An in vitro model of early anteroposterior organization during human development. *Nature* **582**, 410–415 (2020).
70. Liu, X. *et al.* Modelling human blastocysts by reprogramming fibroblasts into iBlastoids. *Nature* **591**, 627 (2021).
71. Rivron, N. C. *et al.* Blastocyst-like structures generated solely from stem cells. *Nature* **557**, 106–111 (2018).
72. Choi, J. *et al.* Prolonged Mek1/2 suppression impairs the developmental potential of embryonic stem cells. *Nature* **548**, 219–223 (2017).
73. Pastor, W. A. *et al.* Naive Human Pluripotent Cells Feature a Methylation Landscape Devoid of Blastocyst or Germline Memory. *Cell Stem Cell* (2016) doi:10.1016/j.stem.2016.01.019.
