# Supplementary material for: Complete human day 14 post-implantation embryo models from naive ES cells
Source: Nature. 2023 Sep 6;622(7983):562–73. doi: 10.1038/s41586-023-06604-5 (PMC10584686; doi:10.1038/s41586-023-06604-5)
Supplement: Supplementary file 2 — Reporting Summary [file 41586_2023_6604_MOESM2_ESM.pdf]

nature portfolio

Corresponding author(s): Jacob H. Hanna

Last updated by author(s): 8/14/2023

Reporting Summary

Nature Portfolio wishes to improve the reproducibility of the work that we publish. This form provides structure for consistency and transparency in reporting. For further information on Nature Portfolio policies, see our Editorial Policies and the Editorial Policy Checklist.

Statistics

For all statistical analyses, confirm that the following items are present in the figure legend, table legend, main text, or Methods section.

n/a	Confirmed	
☐	☒	The exact sample size (*n*) for each experimental group/condition, given as a discrete number and unit of measurement
☐	☒	A statement on whether measurements were taken from distinct samples or whether the same sample was measured repeatedly
☐	☒	The statistical test(s) used AND whether they are one- or two-sided<br>*Only common tests should be described solely by name; describe more complex techniques in the Methods section.*
☐	☒	A description of all covariates tested
☐	☒	A description of any assumptions or corrections, such as tests of normality and adjustment for multiple comparisons
☐	☒	A full description of the statistical parameters including central tendency (e.g. means) or other basic estimates (e.g. regression coefficient) AND variation (e.g. standard deviation) or associated estimates of uncertainty (e.g. confidence intervals)
☐	☒	For null hypothesis testing, the test statistic (e.g. *F*, *t*, *r*) with confidence intervals, effect sizes, degrees of freedom and *P* value noted<br>*Give P values as exact values whenever suitable.*
☒	☐	For Bayesian analysis, information on the choice of priors and Markov chain Monte Carlo settings
☐	☒	For hierarchical and complex designs, identification of the appropriate level for tests and full reporting of outcomes
☒	☐	Estimates of effect sizes (e.g. Cohen's *d*, Pearson's *r*), indicating how they were calculated

Our web collection on statistics for biologists contains articles on many of the points above.

Software and code

Policy information about availability of computer code

Data collection	Single cell sequencing data were collected using Novaseq platform, Illumina. qPCR data were obtained with the Viia7 platform (Applied Biosystems) via QuantStudio software (Version 7 Pro). Microscopy images were acquired with a Zeiss LSM 700, LSM800 inverted confocal microscopes (Carl Zeiss), and the light-sheet microscope (Z7, Carl Zeiss).
Data analysis	The statistical analysis besides single cell and bulk RNA-seq, was performed using the GraphPad Prism 8 software (La Joya, California) and Python v3.8.5, scipy v1.8.0 package; GraphPad Prism 8, Python's matplotlib v3.7.0 and seaborn v0.11.0 packages were used for plotting the data. Multiview fusion and deconvolution of the light-sheet microscopy data was performed in ZEN 3.5 software.<br><br>Fiji/Image J (version 1.52p) was used for image analysis; manual cell counting and cell shape analysis were performed with Imaris v10.0.0 or v10.0.1 (Bitplane).<br><br>10X Genomics data analysis was performed with the Cell Ranger 7.1.0 software (10x Genomics) and Seurat 4.3.0, pheatmap 1.0.12, and Monocole3 R packages v1.3.1<br><br>Multiomics analysis was done also using Signac v1.6.0 and Harmony R V3 packages. Bulk ATAC-seq and RNA-seq were presented with Broad IGV software v2.16.2.<br><br>Flow cytometry data was analyzed using FlowJo v10.7.

For manuscripts utilizing custom algorithms or software that are central to the research but not yet described in published literature, software must be made available to editors and reviewers. We strongly encourage code deposition in a community repository (e.g. GitHub). See the Nature Portfolio guidelines for submitting code & software for further information.

Data

Policy information about availability of data

All manuscripts must include a data availability statement. This statement should provide the following information, where applicable:

- Accession codes, unique identifiers, or web links for publicly available datasets
- A description of any restrictions on data availability
- For clinical datasets or third party data, please ensure that the statement adheres to our policy

Data availability
All newly generated scRNA-seq and 10x Chromium Single Cell Multiome ATAC + Gene Expression data are deposited under GEO: GSE239932. GSE number and reference are indicated for all other previously published and publicly available scRNA-seq and ATAC-seq data are indicated. Any other data is available upon request. All other information required to reanalyze the data reported in this work is available upon request from the corresponding author. Source data are provided with this paper.

Code availability
The custom code generated in this study is provided at GitHub: https://github.com/hannalab/Human_SEM_scAnalysis. The custom code was not essential to the main conclusions of this study.

Human research participants

Policy information about studies involving human research participants and Sex and Gender in Research.

Reporting on sex and gender	Not applicable
Population characteristics	Not applicable
Recruitment	Not applicable
Ethics oversight	Not applicable

Note that full information on the approval of the study protocol must also be provided in the manuscript.

Field-specific reporting

Please select the one below that is the best fit for your research. If you are not sure, read the appropriate sections before making your selection.

☒ Life sciences ☐ Behavioural & social sciences ☐ Ecological, evolutionary & environmental sciences

For a reference copy of the document with all sections, see nature.com/documents/nr-reporting-summary-flat.pdf

Life sciences study design

All studies must disclose on these points even when the disclosure is negative.

Sample size	No statistical methods were used to predetermine sample size. The number of SEMs used in each experiment was used with account of the data consistency/reproducibility and the available resources. Sample size for single cell RNA-Seq was determined when the main cell lineages at each developmental stages were captured.
Data exclusions	For scRNA-seq, to filter out low expressing single cells, possible doublets produced during the 10X sample processing or single cells with extensive mitochondrial expression, we filtered out cells with under 200 expressing genes, over 4000 expressing genes or over 10% mitochondrial gene expression.
Replication	The exact numbers of aggregates and biological replicates used for calculation of SEM protocol efficiency are indicated in the respective figure legends. All data refer to biological replicates and number of samples per biological replicate are indicated in figure legends and Methods section for all relevant panels and for all experiments.
Randomization	Human SEMs were chosen randomly when placed in different culture conditions. For efficiency calculations across conditions and developmental stages, multiple fields of view were imaged from randomly selected experiments and analyzed for an adequate contribution of each lineage with the relevant immunostaining. Number of biological samples/replicates and number of samples per biological replicate are indicated in figure legends and Methods section for all relevant panels. Other experiments were not randomized.
Blinding	The investigators were not blinded to allocation during experiments and outcome assessment. We had no relevant scientific reasons to conduct blinding.

Reporting for specific materials, systems and methods

We require information from authors about some types of materials, experimental systems and methods used in many studies. Here, indicate whether each material, system or method listed is relevant to your study. If you are not sure if a list item applies to your research, read the appropriate section before selecting a response.

Materials & experimental systems

n/a	Involved in the study
☐	☒ Antibodies
☐	☒ Eukaryotic cell lines
☒	☐ Palaeontology and archaeology
☐	☒ Animals and other organisms
☒	☐ Clinical data
☒	☐ Dual use research of concern

Methods

n/a	Involved in the study
☒	☐ ChIP-seq
☐	☒ Flow cytometry
☒	☐ MRI-based neuroimaging

Antibodies

Antibodies used

Mouse monoclonal anti-Oct3/4 (clone C-10) (Santa Cruz Cat# SC-5279), 1:100;
Rabbit polyclonal anti-Oct3/4 (clone H-134) (Santa Cruz Cat# SC-9081), 1:100;
Goat polyclonal anti-Sox17 (R&D Cat# AF1924),1:100;
Rabbit monoclonal anti-Cytokeratin 7 (Abcam Cat# ab181598), 1:200;
Rabbit monoclonal anti-Cytokeratin 7 (Abcam Cat# ab68459), 1:200;
Goat polyclonal anti-Gata3 (R&D Cat# AF2605), 1:100;
Rabbit monoclonal anti-Syndecan1 (Abcam Cat# ab128936), 1:400;
Mouse monoclonal anti-Cdx2 (Biogenex Cat# MU392A-UC), 1:200;
Rabbit monoclonal anti-Phospho-Ezrin (Cell Signaling Cat# 3726), 1:400;
Rabbit monoclonal anti-Brachyury(D2Z3J) (Cell Signaling Cat# 81694), 1:100;
Goat polyclonal anti-Cer1 (R&D Cat# AF1075), 1:100;
Rabbit monoclonal Nanog (Abcam Cat# ab109250), 1:100;
Mouse monoclonal anti-PKC zeta Antibody (H-1) (Santa Cruz Cat# SC-17781), 1:200;
Mouse monoclonal anti-Podocalaxyn [clone 222328] (R&D Cat# MAB1658), 1:200;
Rabbit polyclonal anti-Gata4 (Abcam Cat# ab84593), 1:100;
Mouse monoclonal anti-Vimentin (Abcam Cat# ab8978), 1:100;
Rabbit monoclonal anti-BST2/Tetherin antibody [EPR20202-150] (Abcam Cat# ab243230), 1:100;
Rabbit monoclonal anti-hCG beta [5H4-E2] (Abcam Cat# ab9582), 1:200;
Rabbit monoclonal anti-Gata6 (clone D61E4) (Cell Signaling Cat# 5951), 1:100;
Rabbit monoclonal anti-Islet1 [EP4182] (Abcam Cat# ab109517), 1:100;
Mouse monoclonal anti- Anti-TFAP2a (AP-2α) (3B5) (Santa Cruz Cat# SC-12726), 1:100;
Goat polyclonal anti-Sox2 (R&D Cat# AF2018), 1:200;
Rabbit polyclonal anti-Dnmt3l (Imgenex/Novus Biologicals, Cat# IMG-6804A), 1:100;
Goat polyclonal anti-Otx2 (R&D Cat# AF1979), 1:200;
Mouse molonclonal anti-Stella (D-5 clone) (Santa Cruz Cat# SC-376862), 1:100;
Rabbit monoclonal anti- Blimp1/PDRI-BF1 [Clone C14A4] (Cell Signaling Cat# 9115), 1:100;
Goat polyclonal anti-FoxF1 (R&D Cat# AF4798), 1:100;
Goat polyclonal Nidogen2 (R&D Cat# AF3385), 1:100;
Rabbit monoclonal anti-Gata2 [EPR2822] (Abcam Cat# ab109241), 1:200.

FACS analysis:
Mouse monoclonal anti human TROP2-488 labeled (R&D Cat# FAB650G), 1:20;
Mouse monoclonal anti human CD249 (ENPEP)-BV421 labeled (BD Cat# 744872), 1:20;
Rat monoclonal anti mouse CD140a (PDFGR-a)-PE/Cy7 labeled (BioLegend Cat# 135912), 1:20;
Mouse monoclonal anti human CD140a (PDFGR-a)-PE/Cy7 labeled (BioLegend Cat# 323508), 1:20.
Mouse monoclonal anti human CD140a (PDFGR-a)-APC labeled (BioLegend Cat# 323512), 1:20.

Validation

All the antibodies have been validated by the companies from which they were obtained. Details of the validation statements, antibody profiles and relevant citations can be found on the manufacturer's website provided here.

Mouse monoclonal anti-Oct3/4 (clone C-10) (Santa Cruz Cat# SC-5279) has been referenced in 2450 publications: https://www.scbt.com/p/oct-3-4-antibody-c-10.

Rabbit polyclonal anti-Oct3/4 (clone H-134) (Santa Cruz Cat# SC-9081); has been referenced in 139 publications: https://www.scbt.com/p/oct-3-4-antibody-h-134.

Goat polyclonal anti-Sox17 (R&D Cat# AF1924); has been referenced in 288 publications: https://www.rndsystems.com/products/human-sox17-antibody_af1924?gclid=Cj0KCQjwoeemBhCfARIsADR2QCuGI49R7nTqXVxTxMmT2oKBdmlAHP7HGcpMELWWy1fve2cejy1VYMcaAk-YEALw_wcB&gclsrc=aw.ds

Rabbit monoclonal anti-Cytokeratin 7 (Abcam Cat# ab181598); has been referenced in 91 publications: https://www.abcam.com/products/primary-antibodies/cytokeratin-7-antibody-epr17078-cytoskeleton-marker-ab181598.html

Rabbit monoclonal anti-Cytokeratin 7 (Abcam Cat# ab68459); has been referenced in 31 publications: https://www.abcam.com/products/primary-antibodies/cytokeratin-7-antibody-epr1619y-cytoskeleton-marker-ab68459.html

Goat polyclonal anti-Gata3 (R&D Cat# AF2605); has been referenced in 19 publications: https://www.rndsystems.com/products/human-gata-3-antibody_af2605?gclid=Cj0KCQjwoeemBhCfARIsADR2QCuyed6_9X10TlOJlqu7BWtjQsRWKClUtoMxUbi_qSHy8gP4zR_PnBUaAhFaEALw_wcB&gclsrc=aw.ds

Rabbit monoclonal anti-Syndecan1 (Abcam Cat# ab128936); has been referenced in 39 publications: https://www.abcam.com/products/primary-antibodies/syndecan-1-antibody-epr6454-ab128936.html

Mouse monoclonal anti-Cdx2 (Biogenex Cat# MU392A-UC); has been referenced in 49 publications: https://www.labome.com/product/Biogenex/MU392A-UC.html

Rabbit monoclonal anti-Phospho-Ezrin (Cell Signaling Cat# 3726); has been referenced in 86 publications: https://www.cellsignal.com/products/primary-antibodies/phospho-ezrin-thr567-radixin-thr564-moesin-thr558-48g2-rabbit-mab/3726?_requestid=664905

Rabbit monoclonal anti-Brachyury(D2Z3J) (Cell Signaling Cat# 81694); has been referenced in 27 publications: https://www.cellsignal.com/products/primary-antibodies/brachyury-d2z3j-rabbit-mab/81694

Goat polyclonal anti-Cer1 (R&D Cat# AF1075); has been referenced in 3 publications: https://www.rndsystems.com/products/human-cerberus-1-antibody_af1075

Rabbit monoclonal Nanog (Abcam Cat# ab109250); has been referenced in 154 publications: https://www.abcam.com/products/primary-antibodies/nanog-antibody-epr20272-ab109250.html

Mouse monoclonal anti-PKC zeta Antibody (H-1) (Santa Cruz Cat# SC-17781); has been referenced in 134 publications: https://www.scbt.com/p/pkc-zeta-antibody-h-1

Mouse monoclonal anti-Podocalaxyn [clone 222328] (R&D Cat# MAB1658); has been referenced in 13 publications: https://www.rndsystems.com/products/human-podocalyxin-antibody-222328_mab1658

Rabbit polyclonal anti-Gata4 (Abcam Cat# ab84593); has been referenced in 63 publications: https://www.abcam.com/products/primary-antibodies/gata4-antibody-ab84593.html

Mouse monoclonal anti-Vimentin (Abcam Cat# ab8978); has been referenced in 507 publications: https://www.abcam.com/products/primary-antibodies/vimentin-antibody-rv202-cytoskeleton-marker-ab8978.html

Rabbit monoclonal anti-BST2/Tetherin antibody [EPR20202-150] (Abcam Cat# ab243230); has been referenced in 2 publications: https://www.abcam.com/products/primary-antibodies/bst2tetherin-antibody-epr20202-150-ab243230.html

Rabbit monoclonal anti-hCG beta [5H4-E2] (Abcam Cat# ab9582); has been referenced in 22 publications: https://www.abcam.com/products/primary-antibodies/hcg-beta-antibody-5h4-e2-ab9582.html

Rabbit monoclonal anti-Gata6 (clone D61E4) (Cell Signaling Cat# 5951); has been referenced in 91 publications: https://www.cellsignal.com/products/primary-antibodies/gata-6-d61e4-xp-rabbit-mab/5851

Rabbit monoclonal anti-Islet1 [EP4182] (Abcam Cat# ab109517); has been referenced in 52 publications: https://www.abcam.com/products/primary-antibodies/islet-1-antibody-ep4182-neural-stem-cell-marker-ab109517.html

Mouse monoclonal anti- Anti-TFAP2a (AP-2α) (3B5) (Santa Cruz Cat# SC-12726); has been referenced in 91 publications: https://www.scbt.com/p/ap-2alpha-antibody-3b5

Goat polyclonal anti-Sox2 (R&D Cat# AF2018); has been referenced in 195 publications: https://www.rndsystems.com/products/human-mouse-rat-sox2-antibody_af2018

Rabbit polyclonal anti-Dnmt3l (Imgenex Cat# IMG-6804A); has been referenced in 2 publications: https://www.novusbio.com/products/dnmt3l-antibody_nbp2-27098

Goat polyclonal anti-Otx2 (R&D Cat# AF1979); has been referenced in 81 publications: https://www.rndsystems.com/products/human-otx2-antibody_af1979

Mouse moloncional anti-Stella (D-5 clone) (Santa Cruz Cat# SC-376862); has been referenced in 2 publications: https://www.scbt.com/p/stella-antibody-d-5

Rabbit monoclonal anti- Blimp1/PDRI-BF1 [Clone C14A4] (Cell Signaling Cat# 9115); has been referenced in 69 publications: https://www.cellsignal.com/products/primary-antibodies/blimp-1-prdi-bf1-c14a4-rabbit-mab/9115

Goat polyclonal anti-FoxF1 (R&D Cat# AF4798); has been referenced in 10 publications: https://www.rndsystems.com/products/human-mouse-foxf1-antibody_af4798?gclid=Cj0KCQjwoeemBhCfARIsADR2QCsjgD6WVshK8vZ2JC6h6tk6e4Wn2emSIPup0TER4GhFfDXQoWac2-AaAiJkEALw_wcB&gclsrc=aw.ds

Goat polyclonal Nidogen2 (R&D Cat# AF3385); has been referenced in 3 publications: https://www.rndsystems.com/products/human-nidogen-2-antibody_af3385

Rabbit monoclonal anti-Gata2 [EPR2822] (Abcam Cat# ab109241); has been referenced in 10 publications: https://www.abcam.com/products/primary-antibodies/gata2-antibody-epr28222-ab109241.html

Antibodies for flow cytometry: All the antibodies guarantee covers the use of the antibody for flow cytometry applications.

Mouse monoclonal anti human TROP2-488 labeled (R&D Cat# FAB650G). The antibody has been pre-titrated and tested by flow cytometry analysis of PC-3 human prostate cancer cell line. The antibody has been referenced in 2 publications: https://www.rndsystems.com/products/human-trop-2-alexa-fluor-488-conjugated-antibody-77220_fab650g

Mouse monoclonal anti human CD249 (ENPEP)-BV421 labeled (BD Cat# 744872). The production process of this antibody underwent stringent testing and validation to assure that it generates a high-quality conjugate with consistent performance and specific binding activity. This antibody has been referenced in 4 publications: https://www.bdbiosciences.com/en-eu/products/reagents/flow-cytometry-reagents/research-reagents/single-color-antibodies-ruo/bv421-mouse-anti-human-cd249.744872

Rat monoclonal anti mouse CD140a (PDFGR-a)-PE/Cy7 labeled (BioLegend Cat# 135912). Each lot of this antibody is quality control tested by immunofluorescent staining with flow cytometric analysis. This antibody has been referenced in 5 publications: https://www.biolegend.com/en-us/products/pe-cyanine7-anti-mouse-cd140a-antibody-14822?GroupID=BLG8103

Mouse monoclonal anti human CD140a (PDFGR-a)-PE/Cy7 labeled (BioLegend Cat# 323508) and APC labeled (BioLegend Cat# 323512). Each lot of this antibody is quality control tested by immunofluorescent staining with flow cytometric analysis. This antibody has been referenced in 15 publications: https://www.biolegend.com/en-us/products/pe-anti-human-cd140a-pdgfralpha-antibody-3727?GroupID=BLG5119

Eukaryotic cell lines

Policy information about cell lines and Sex and Gender in Research

Cell line source(s)

WIBR1 human male, WIBR2, WIBR3 Human female embryonic stem cell lines were previously reported in Lenger et al. Cell 2010 and provided by the last author of that paper: Prof. Rudolf Jaenisch, Whitehead Institute of Science, Cambridge, MA, USA. RUES2 hESC line was previously described in Simunovich et al. Cell Stem Cell 2002 and provided by the last author, Prof. A Brivanlou, Rockefeller University, USA.

Authentication

Karyotype and sequencing data confirmed expected sex and karyotype, gene reporters and cell identity via SNPs.

Mycoplasma contamination

All cell lines tested negative for mycoplasma contamination by using the MycoAlert plasma Detection Kit (Lonza, Cat# LT07-318) and were routinely screened every 1 month.

Commonly misidentified lines
(See ICLAC register)

HEK293T cells were used for lentivirus generation only that was used to permanently labeled some cell lines as indicated in the paper. HEK293T cells were cultured in a dedicated tissue culture room that is separate from where ESC culture and SEM generation were performed.

Animals and other research organisms

Policy information about studies involving animals; ARRIVE guidelines recommended for reporting animal research, and Sex and Gender in Research

Laboratory animals

Mus Musculus (mouse) ICR strain derived embryo samples were used as reference controls for mouse SEM related experiments. 4-10 week old male and female ICR mice were used for timed matings for natural embryo dissection.

Wild animals

The study did not involve wild animals

Reporting on sex

Sex of ES and iPS lines used (male and female) is indicated for all lines used and we do not report any sex bias of difference in result outcome. WIBR1 is a male hESC lines, WIBR2 and WIBR3 are female hESC line. JH22 and JH33 are male human iPSC lines. V6.5 and BVSC arei male mouse ESC line.

Field-collected samples

The study did not involve samples collected from the field

Ethics oversight

Mouse animal experiments pertained only to mouse SEM and comparing them to mouse embryos, and were performed according to the Animal Protection Guidelines of Weizmann Institute of Science and approved by the following Weizmann Institute IACUC (#01390120-1, 01330120-2, 33520117-2).

Note that full information on the approval of the study protocol must also be provided in the manuscript.

Flow Cytometry

Plots

Confirm that:

☒ The axis labels state the marker and fluorochrome used (e.g. CD4-FITC).

☒ The axis scales are clearly visible. Include numbers along axes only for bottom left plot of group (a 'group' is an analysis of identical markers).

☒ All plots are contour plots with outliers or pseudocolor plots.

☒ A numerical value for number of cells or percentage (with statistics) is provided.

Methodology

Sample preparation	Cells were incubated for half an hour with fluorophore-conjugated antibodies (1:50) in PBS/0.5% BSA.
Instrument	BD FACS-Aria III
Software	FlowJo v10.7
Cell population abundance	Only one cell population was analyzed post-sorting, and the purity was verified by resampling.
Gating strategy	FSC and SSC singlets were gated to remove debris and aggregated cells, and only single cells were considering for all analyses. To determine the gating for positive or negative populations, an unstained control and naive PSCswere employed, making sure that approximately 100% of the unstained population was allocated on the negative area of the histogram/dot plot. Gating strategies are included in the last Supplementary Figure S17.

☒ Tick this box to confirm that a figure exemplifying the gating strategy is provided in the Supplementary Information.
